# Virus-neutralizing monoclonal antibodies against bovine viral diarrhea virus and classical swine fever virus target conformational and linear epitopes on E2 glycoprotein subdomains

**DOI:** 10.1128/spectrum.02041-24

**Published:** 2025-02-25

**Authors:** Gleyder Roman-Sosa, Denise Meyer, Mariano Dellarole, Doris à Wengen, Susanne Lerch, Alexander Postel, Paul Becher

**Affiliations:** 1 Institute of Virology, University of Veterinary Medicine, Hannover, Germany; 2 EU & WOAH Reference Laboratory for Classical Swine Fever, University of Veterinary Medicine, Hannover, Germany; 3 CIBION, CONICET 62873 , Buenos Aires, Argentina; Changchun Veterinary Research Institute, Changchun, China

**Keywords:** classical swine fever virus, bovine viral diarrhea virus, glycoprotein E2, epitope mapping, conformational epitope, virus neutralization, pestiviruses, monoclonal antibodies

## Abstract

The envelope glycoprotein E2 of pestiviruses plays a crucial role in viral entry and elicits a virus-neutralizing humoral immune response. Consequently, the epitopes recognized by monoclonal antibodies (mAbs) on E2 are a significant focus in pestivirus research and diagnostics. In this study, we characterized a panel of murine mAbs against the E2 protein of classical swine fever virus (CSFV) and bovine viral diarrhea virus (BVDV), two major pathogens for swine and cattle, respectively. The majority of mAbs neutralized the virus *in vitro* and recognized conformational epitopes, which were also detected by sera from infected animals. Notably, binding to these epitopes was retained after low-pH treatment, although conformational epitopes were disrupted upon disulfide bond reduction. The epitopes of the anti-CSFV mAbs were located in various domains of E2, including the interdomain linker sequences. Conversely, all but one of the anti-BVDV mAb epitopes were located in domain A. Moreover, the reactivity of one mAb suggests a conformational interdependence among the linker sequences of pestivirus E2. The panel of mAbs characterized in this study holds potential to support basic research on the mechanism of early pestivirus invasion and to assist in the design of E2-based diagnostic tools and vaccines.

**IMPORTANCE:**

Classical swine fever virus (CSFV) and bovine viral diarrhea virus (BVDV), which belong to the family Flaviviridae, cause economically significant diseases in pigs and cattle. The pestivirus glycoprotein E2 is located on the viral surface and is targeted by antibodies that neutralize virus infection. Due to its variability, E2 is a useful antigen for the development of diagnostic tests to differentiate between infections caused by different pestiviruses. In the present study, two panels of monoclonal antibodies (mAbs) specifically reactive with either CSFV or BVDV E2 were characterized. Interestingly, the majority of mAbs neutralized the respective virus *in vitro*. Epitope mapping revealed that the mAbs recognized low-pH-resistant epitopes of conformational nature located in different domains of CSFV E2 (anti-CSFV mAbs) or in domain A of BVDV E2 (anti-BVDV mAbs). The recombinant proteins along with the characterized mAbs have the potential to develop improved pestivirus-specific diagnostic tests and vaccines.

## INTRODUCTION

Pestiviruses are classified within the genus *Pestivirus* of the family *Flaviviridae*, which also includes the genera *Flavi-*, *Hepaci*-, and *Pegivirus* (reviewed in reference [1]). Currently, the International Committee on Taxonomy of Viruses recognizes 19 pestivirus species (2). Significant pathogens within this genus include classical swine fever virus (CSFV, species *Pestivirus suis*), bovine viral diarrhea virus (BVDV-1, species *Pestivirus bovis* and BVDV-2, species *Pestivirus tauri*), and border disease virus (BDV, species *Pestivirus ovis*). CSFV primarily affects swine and is the causative agent of a highly relevant re-emerging disease (3), whereas BVDV mainly affects cattle. BDV predominantly infects small ruminants (4, 5) and can also infect swine and cattle (6–9). Infection of pigs with BDV and other ruminant pestiviruses can lead to cross-reactions, interfering with the serological diagnosis of CSFV due to the close antigenic relationship among these viruses (10).

The pestiviral genome is a single-stranded monocistronic, positive-sense RNA molecule consisting of ~12.3 kilobases that encodes one large polyprotein (11–13). This polyprotein is proteolytically processed into four mature structural proteins (C, Erns, E1, and E2) (14, 15), which are found in the viral particle (16), and eight non-structural (NS) proteins (Npro, p7, NS2, NS3, NS4A, NS4B, NS5A, and NS5B) (17–19) (reviewed in reference [20]). The structural glycoprotein E2, initially referred to as E1, plays a crucial role during viral pathogenesis and elicits a virus-neutralizing humoral immune response (21, 22). E2, along with E1, was shown to be sufficient to mediate infection *in vitro* by pseudotyped vesicular stomatitis virus (VSV) particles carrying them on their surface (23, 24).

Studies with CSFV full-length infectious clones demonstrated that introducing the E2 sequence of the vaccine lapinized C-strain (CS) into the backbone of the virulent strain Brescia led to attenuation *in vivo* (25). Moreover, mutating the TAVSPTTLR epitope or the N-glycosylation sites of CSFV E2 resulted in a less virulent phenotype (26, 27). Additionally, the E2 protein of CSFV has been shown to interact with the porcine attachment factor ADAM17 (28), as well as several other cellular factors such as SERTAD1 (29), Torsin-1A (30), and ACADM (31). An antigenic map based on the reactivity of the E2 protein of CSFV strain Brescia with monoclonal antibodies (mAbs) identified domain A (amino acids 766 to 866), domains B (amino acids 691 to 773) and C (amino acids 691 to 800), often referred to as domain B/C due to overlap, and domain D (amino acids 766 to 800) (32).

The structure of the BVDV E2 has been solved, revealing that the protein is organized into four domains: domain A, domain B, domain C, and domain D (33, 34). In this study, we will refer to this structure-guided nomenclature of the domains. For clarity, we provide the following information: the antigenic domain B and most of domain C (35) are within the structural domain A (33, 34), while antigenic domain A (35) encompasses the last five residues of structural domain A, the interdomain A-B linker, the structural domain B, the interdomain B-C linker, and the first eight residues of domain C. mAbs against the E2 protein have been extensively used in serological diagnosis and antigen detection of pestiviruses (36–39), and have been crucial for specifically detecting CSFV infections (40). Monoclonal antibodies have facilitated the identification of E2 as an important target for virus neutralization (41, 42), as well as the detection of molecular determinants crucial for virus infection (43, 44).

Frequently, mAbs are produced against recombinant ectodomains of the glycoprotein E2 (44, 45). While this approach is convenient, certain structural elements, such as quaternary epitopes, are lost when the protein is not expressed in the context of the viral particle (46, 47).

In the present study, we aimed to characterize anti-E2 mAbs generated against pestiviral virions and used them to probe structural changes and to determine the regions mediating virus neutralization. The obtained results demonstrate that these mAbs hold potential for diagnostics and vaccine development. They primarily recognized conformational epitopes that were resistant to low-pH treatment, suggesting an interdependence of the conformation among interdomain linker regions of E2.

## MATERIALS AND METHODS

### Monoclonal antibodies

The previously described mAb BM40 (directed against Brucella melitensis 16M) was applied as a negative control (48). The hybridoma cell lines producing mAbs against the E2 proteins of CSFV and BVDV were generated between 1985 and 1990 at the Institute of Virology, University of Veterinary Medicine. The protocol for the generation of the hybridoma cell lines has been described previously (38, 49, 50). Briefly, the pestivirus strains used for immunization (Table 1) of mice were propagated on porcine or bovine cells, harvested, and concentrated by ultracentrifugation. The virus preparations were used to immunize Balb/c mice or mice with the Robertsonian chromosome translocation 8.12 (RB 8.12). The spleen of the mice was aseptically isolated, the cells were separated from the connective tissue and suspended in phosphate-buffered saline (PBS). Subsequently, these cells were mixed with the mouse myeloma cell line FOX-NY in a ratio of 2:1 (spleen cells:myeloma cells) and were centrifuged. For cell fusion, approximately 0.7 mL Polyethylenglycol 1500 (PEG) was added to the cell pellet, carefully stirred with a pipette, and incubated in a 37°C water bath for 1 min. PEG was diluted by slowly adding 20 mL of selective AAT medium (Dulbecco’s medium with 7.5 × 10^−5^ M adenine, 1 × 10^−4^ M hypoxanthine, 4 × 10^−7^ M aminopterin, and 1.6 × 10^−5^ M thymidine supplemented with 15% horse serum [HS]). Afterward, the fused cells were mixed with myeloma feeder cells (~0.5 × 10^8^ feeder cells per 96-well plate), and the volume was adjusted with the AAT medium and distributed in 16 96-well flat-bottomed plates (applying 180 µL/well). The plates were incubated at 37°C and 4%–5% CO_2_. The screening of the hybridoma supernatants for the presence of anti-pestivirus antibodies was mainly applied by an indirect immune peroxidase or fluorescence assay using cells infected with the homologous pestivirus. The monoclonality of the hybridomas was achieved either by diluting the cell suspensions or by isolating the colonies grown in soft agar.

**TABLE 1:** Monoclonal antibodies detecting the E2 protein of CSFV or BVDV

Antigen		Name	Ig-isotype	Reference
Virus	Strain			
CSFV	Alfort/187	HC/TC3	IgG2b	(49)
		HC/TC16	IgG1	(49)
		HC/TC18	IgG2b	(49)
		HC/34	IgG1	(49)
		HC/36	IgG1	(49)
		HC/37	IgG1	(49)
		HC/43	IgG1	(49)
		HC/TC50	IgG2b	This study
		HC/TC54	IgG1	This study
		HC/TC59	IgG2a	This study
		HC/TC62	IgG1	This study
		HC/TC63	IgG1	This study
		HC/TC64	IgG2b	This study
		HC/TC65	IgG3	This study
		HC/TC68	IgG1	This study
BVDV	0712/80	BVD/PX1	IgG1	This study
	NADL	BVD/PX8	IgG2a	This study
	NADL	BVD/PX14	IgG2b	This study
	NADL	BVD/PX18	IgG2a	This study
	NADL	BVD/CA1	IgG1	(38)
	NADL	BVD/CA3	IgG2b	(38)
	7443	BVD/CA34	IgG2a	(38)
	Singer	BVD/CA72	IgM	(38)
	Singer	BVD/CA73	IgG1	(38)
	Singer	BVD/CA80	IgM	(38)
	Singer	BVD/CA82	IgG1	(38)
	1138 FRG	BVD/CT3	IgG2a	(50)
	1138 FRG	BVD/CT6	IgG2a	(50)

### Production and purification of mAbs

Murine hybridomas were propagated according to cell culture standard procedures in Dulbecco’s Modified Eagle’s Medium (DMEM) containing 8%–10% horse serum (49), and the supernatants were collected and kept sterile at −20°C until use. The material was deprived of serum immunoglobulins before the mAbs were purified by affinity chromatography with protein G NAB Spin columns (Thermo Fisher Scientific, Dreieich, Germany) or 1 mL HP protein G columns (Cytiva, Freiburg, Germany) according to the manufacturer’s instructions, respectively. The fractions containing the mAb were dialyzed against PBS and were stored at −80°C until use. The respective hybridomas of the anti-BVDV mAbs BVD/CA72 and BVD/CA80 produced very low amounts of antibody, and the yields after purification were not amenable for further experiments.

### Cells and viruses

Porcine kidney-15 (PK-15) and Madin–Darby bovine kidney (MDBK) cells were maintained in minimum essential medium (MEM) supplemented with 10% fetal bovine serum (FBS), which was tested free for pestivirus genomes and antibodies, and were incubated at 37°C in 5% CO_2_.

A panel of pestiviruses including nine CSFV strains, two BVDV-1 strains, and two BVDV-2 strains were propagated at the EU and WOAH Reference Laboratory for CSF, Institute of Virology, University of Veterinary Medicine, Hannover, Germany (Table 4).

### Virus neutralization test

The virus-neutralizing activity of the anti-CSFV and anti-BVDV mAbs was assessed by testing defined concentrations of affinity-purified antibodies with 30–300 tissue culture infectious dosis 50 (TCID_50_) of CSFV Alfort/187 or BVDV NADL, respectively, following the procedures of the manual of diagnostic tests for detection of classical swine fever (51) and the Laboratory of Viral Diagnostics of the Institute of Virology of the University of Veterinary Medicine Hannover, Foundation (52). The amounts (µg/well) of anti-CSFV mAbs tested were 5, 2.5, 1.25, 0.625, 0.312, 0.156, 0.078, 0.039, 0.0195, 0.00975, 0.00487, and 0.00244. The following amounts (µg/well) of anti-BVDV mAbs were tested: 50, 25, 12.5, 6.25, 3.12, and 1.56. The BVDV mAbs BVD/CA72 and BVD/CA80 were not tested as not enough amounts could be purified. The results were expressed as the nanomolar concentration of mAb that neutralizes virus infection in 50% of the wells, considering a 150 kDa molecular weight of the mouse immunoglobulins.

### Antibody reactivity with different CSFV, BVDV-1, and BVDV-2 strains

To investigate the reaction spectrum of the E2 mAbs with different CSFV, BVDV-1, and BVDV-2 strains, cells were simultaneously infected with the viruses listed in Table 4 and were seeded in 96-well plates to obtain an infection rate of 70%–90% of the cell layer. This infection rate was chosen to evaluate possible background on non-infected cells in direct comparison to infected cells in one well.

PK-15 cells were used for the infection with CSFV, and MDBK cells for the infections with BVDV-1 and BVDV-2, respectively. At 72 h postinfection, the cells were fixed by heat treatment at 80°C for 3 h (PK-15 cells) or 5 h (MDBK cells), and an indirect immune-peroxidase assay was performed as previously described (53). Together with the E2 mAbs, a pestivirus NS3-specific antibody (mAb BVD/C16) was used to confirm the cellular infection rate of 70%–90%.

### Generation of plasmids for protein expression

The sequences encoding for all proteins were cloned into a plasmid optimized for protein expression in mammalian cells (54, 55). The vector pHL-Sec-AviTag-Thr-dStrepTag (pHL-StrepTag) with an AviTag, thrombin cleavage site, and Twin StrepTag-encoding sequences at the carboxyl terminus (G. Roman-Sosa, unpublished data) was used as a backbone for all constructs. Construction of the expression plasmids encoding CSFV E2, BVDV E2, and chimeric E2 proteins was based on previously published data and constructs (56). The sequences of all plasmids were controlled by Sanger sequencing (LGC Genomics, Berlin, Germany). The full sequences of all constructs are available as Supplemental material. Further details of cDNA cloning can be obtained upon request. A schematic representation of the individual proteins is provided in Figure 7A. The first part of the name of the chimeric constructs refers to the main backbone protein and is followed by “p,” indicative of partial, and the sequence that was inserted. After the name of each construct, an exact description of the encoded protein is given. The following plasmids were constructed: (i) pCSFV E2: encoding for E2 protein ectodomain of classical swine fever virus strain Alfort/187 (Gln 690-Ser1025); (ii) pCSFV E2-pBVDV-B-C: encoding for CSFV E2 protein ectodomain with part of BVDV domains B and C (CSFV Gln 690-Val 799/BVDV Val 804-Gly 867/CSFV Glu 864-Ser 1025); (iii) pCSFV E2-pBVDV-B: encoding for CSFV E2 protein ectodomain with part of BVDV domain B (CSFV Gln 690-Val 799/BVDV Val 804-Ser 831/CSFV Cys 828-Ser 1025); (iv) pBVDV E2-pCSFV-B-C: encoding for BVDV E2 protein ectodomain with part of CSFV domains B and C (BVDV His 693-Ile 803/CSFV Val 800-Asn 863/BVDV Glu 1029-Arg 1029); (v) pCSFV E2-pBVDV-A-B: encoding for CSFV E2 protein ectodomain with part of BVDV domains A and B (CSFV Glu 690-Ala 746/BVDV Arg 750-Ile 863/CSFV Val 800-Ser 1025); (vi) pCSFV E2-pBVDV-A: encoding for CSFV E2 protein ectodomain with part of BVDV domain A (CSFV Gln 690-Val 749/BVDV Arg 753-Gln 781/CSFV Ser 779-Ser 1025); (vii) pCSFV E2-pBVDV-B-1: encoding for CSFV E2 protein ectodomain with part of amino terminus of BVDV domain B (CSFV Glu 690-Pro 778/BVDV Asp 783-Ile 803/CSFV Val 800-Ser 1025); (viii) pCSFV-A: encoding for CSFV E2 protein domain A (CSFV Gln 690-Thr 776); (ix) pCSFV-A-pBVDV-A: encoding for CSFV E2 protein domain A with amino terminal part of BVDV domain A (BVDV His 693-Glu 716/CSFV Gly 714-Thr 776); (x) pBVDV E2: encoding for E2 protein ectodomain of BVDV strain NADL (His 693-Arg 1029); and (xi) pBVDV A: encoding for BVDV E2 protein domain A (His 693-Arg 779).

### Protein expression and purification

The purification of the recombinant proteins was carried out essentially as described (54, 57). Briefly, HEK293T (*h*uman *e*mbryo *k*idney) cells were transiently transfected with a mixture of plasmid DNA encoding for the complete BVDV- or CSFV E2 antigen or for the chimeric E2 constructs and polyethylenimine (PEI; Sigma-Aldrich, Taufkirchen, Germany) at a ratio of 3 µg PEI:1 µg DNA in serum-free medium. The PEI:DNA complexes were allowed to form during 15 min of incubation at room temperature before being added to the cells in serum-free medium. The cells were then incubated with the transection mixture at 37°C in a 0.5% CO_2_ atmosphere for 3 h, after which the medium was exchanged for a complete medium containing 10% fetal calf serum (FCS). Three days after transfection, the supernatants were collected. The supernatants were clarified by centrifugation (5,000 × *g*/25 min/4°C) and concentrated by tangential filtration using a Vivaflow cassette (Vivaflow system, cassette with membrane of molecular mass cutoff [MWCO] 5,000 Da; Sartorius Stedim Biotech; further details available at https://www.sartorius.com/en/products/lab-filtration-purification/ultrafiltration-devices/tangential-crossflow). The biotin in the starting material was neutralized with 500 µL BioLock (IBA-Biotech, Göttingen, Germany), and the proteins were affinity purified with a StrepTrapXT column (IBA-Biotech, Göttingen, Germany) in an Äkta pure chromatographic unit (GE Healthcare, Freiburg, Germany). The column was pre-equilibrated with buffer W (100 mM Tris/HCl [pH 8.0], 150 mM NaCl, 1 mM EDTA) before the material was applied at a flow rate of 1 mL/min. The column was then washed with 15 mL buffer W, and the proteins were eluted with buffer E (buffer W plus 2.5 mM desthiobiotin). Ten fractions each of 1 mL were collected. The protein concentration was measured with a spectrophotometer (NanoDrop 2000, Thermo Fisher Scientific, Dreieich, Germany), and the proteins of the collected fractions were separated by sodium dodecyl sulfate-polyacrylamide gel electrophoresis (SDS-PAGE) under reducing conditions and stained with Protein Gel Stain (German Research Products, Haag a.d. Amper, Germany). The full-length ectodomains and the A subdomains were analyzed in gels with 10% and 15% polyacrylamide, respectively. A prestained protein ladder (PageRuler Plus, Thermo Fisher Scientific, Dreieich, Germany) was used to estimate the molecular weight of the stained protein bands. The fractions were then pooled and concentrated as required (10,000 MWCO PES concentrators, Sartorius), snap frozen with liquid nitrogen, and stored at −80°C until use.

### Enzyme-linked immunosorbent assays (ELISAs)

#### Indirect ELISA to test the reactivity of the mAbs with the recombinant CSFV E2 and BVDV E2 proteins

The mAbs were tested in duplicate with each protein. ELISA plates with a medium binding capacity (Nunc plates, Thermo Fisher Scientific, Dreieich, Germany) were coated with 50 ng of the respective protein per well in 100 µL 0.1 M carbonate coating buffer. The control wells were coated with 3% bovine serum albumin (BSA) (Serva, Heidelberg, Germany) in PBS/0.05% Tween 20, overnight at 4°C. The plates were then washed three times with wash buffer (PBS/0.05% Tween 20), and the wells’ surface was saturated with 100 µL of blocking solution (3% BSA in PBS/0.05% Tween 20). The hybridoma supernatants of the anti-CSFV mAbs (Table 1) were tested undiluted, whereas the anti-BVDV mAbs (Table 1) were diluted 1:5 in wash buffer. However, for BVD/PX8, a 1:2 dilution was applied. As a negative control, the supernatant of the mAb BM40 was used. The anti-StrepTag mAb (StrepMAB Classic) (IBA Biotech, Göttingen, Germany) was used 1:1,000 (anti-CSFV mAbs) or 1:2,000 (anti-BVDV mAbs) diluted in wash buffer as a positive control. After 1 h of incubation at 37°C in a humid chamber, the plates were washed as mentioned above and incubated with 100 µL/well of rabbit anti-mouse conjugated to horseradish peroxidase (HRP) (former DAKO, Agilent, Waldbronn, Germany) diluted 1:1,000 in wash buffer. The plates were incubated at 37°C for 1 h in a humid chamber and were washed as mentioned above. Each well was incubated for 3 min in the dark with 100 µL TMB (3,3′,5,5′-Tetramethylbenzidine) (Sigma-Aldrich, Taufkirchen, Germany) solution per well. The reaction was stopped by adding 100 µL of 1 M HCl per well, and the optical density was measured at 450 nm in an ELISA reader (Tecan). The results were expressed by subtracting the background optical density (OD) at 450 nm for each mAb tested with the control wells from the ones obtained with the E2 antigen. Three independent experiments were performed, and the standard deviation was calculated.

#### Indirect ELISA to test the reactivity of the mAbs with the chimeric recombinant CSFV E2 and BVDV E2 proteins

The ELISA to test the reaction of the mAbs with the corresponding chimeric proteins was essentially performed as described above with 50 ng per well of the respective recombinant protein. Three independent experiments were performed, and the standard deviation was calculated.

The results are expressed as a ratio to non-mutagenized E2 (wild-type; wt) using the following formula:


$$OD\;ratio\;to\;wt\;E2=\frac{OD\;mAb\;with\;chimeric\;E2}{OD\;mAb\;with\;wt\;E2}$$

#### Indirect ELISA to test the effect of low-pH treatment on the reactivity of the mAbs

The ELISA plates were prepared with 50 ng protein/well as described in (I). Before the incubation with the mAbs, the proteins were treated at low pH on the ELISA plate. This step was performed by washing the corresponding wells with 200 µL of citrate buffer pH 3, pH 4, or pH 5, then 250 µL of the corresponding low-pH buffer was applied, and the wells were incubated for 10 min at room temperature. The buffer was then discarded, and the wells were washed with wash buffer. Each mAb was tested in parallel with non-treated wells on the same plate. Three independent experiments were performed, and the standard deviation was calculated.

The results are expressed as a ratio to E2 without low-pH treatment using the following formula:


$$OD\;ratio\;to\;non\text{-}treated\;E2=\frac{OD\;mAb\;with\;low\text{-}pH\text{-}treated\;E2}{OD\;mAb\;with\;non\text{-}treated\;E2}$$

#### Indirect ELISA to test the effect of disulfide bridge reduction on the reactivity of the mAbs

The test was performed essentially as previously described (52). Briefly, 50 ng of E2 protein was treated with 10 mM dithiothreitol (DTT) (Sigma-Aldrich, Taufkirchen, Germany) at 95°C for 5 min, and the cysteines were alkylated by subsequent treatment with 10 mg/mL iodoacetamide (Sigma-Aldrich, Taufkirchen, Germany) for 15 min at 37°C. The mAbs were tested with the reduced and the non-treated proteins as described in (I). The hybridoma supernatants of the anti-CSFV mAbs were tested undiluted, whereas the supernatants of anti-BVDV mAbs were diluted 1:5 except the mAb BVD/PX 8 that was diluted 1:2 because of the much lower signal compared to the other antibodies. Three independent experiments were performed, and the standard deviation was calculated.

The results are expressed as a ratio to non-treated E2 using the following formula:


$$OD\;ratio\;to\;non\text{-}treated\;E2=\frac{OD\;mAb\;with\;reduced\;E2}{OD\;mAb\;with\;non\text{-}treated\;E2}$$

#### Indirect ELISA to test the binding strength of purified mAbs

The ELISA was performed as described in (I) using 50 ng protein/well but with different amounts of purified mAb per well (40, 20, 10, 5, 2.5, 1.25, and 0.625 ng). The mAbs were tested in triplicate (i.e., three wells per mAb with each antigen concentration).

### Blocking of the reactivity of the mAbs by sera from infected animals

The ELISA plates were prepared as described in the ELISA section (I). The blocking effect on the anti-CSFV mAbs was analyzed using a homologous serum (serum ID: 2006/07/0057/065) generated by infecting a pig with CSFV Alfort/187. This serum is a reference serum that is routinely applied in diagnostic assays to detect antibodies against CSFV. It is characterized by a positive result in commercial CSF antibody ELISAs and a high titer of neutralizing antibodies against the CSFV strain Alfort/187 (ND_50_ = 1,600). This serum was obtained from the serum sample collection of the EU and WOAH Reference Laboratory for CSF (51). The bovine serum 21/227/1 raised against BVDV strain NADL (Laboratory for Diagnostic Virology, Institute of Virology, University of Veterinary Medicine Hannover) was used for the anti-BVDV mAbs. This serum is characterized by a high titer of neutralizing antibodies against the BVDV-1 strain NADL (ND_50_ = 1,280).

The sera were tested diluted 1:2 in dilution buffer (PBS/0.05% Tween 20 containing 2% BSA) and incubated on the plate for 1 h at 37°C in a humid chamber. The wells were washed three times with wash buffer, and the reaction of the mAbs with the protein was performed as described in the ELISA section (I). In the case of the anti-CSFV mAbs, the anti-mouse HRP conjugate was used in a dilution of 1:3,000 with 10% of swine serum (Kraeber & Co GmbH, Ellerbek, Germany) in the conjugate dilution buffer. The anti-BVDV mAbs were detected with the anti-mouse HRP conjugate diluted 1:2,000 in the conjugate dilution buffer with 10% of fetal calf serum, negative for neutralizing antibodies against BVDV (Capricorn Scientific GmbH, Ebsdorfergrund, Germany).

The anti-CSFV mAbs were tested in duplicate in three independent experiments. The anti-BVDV mAbs were tested in triplicate in one experiment. A graph with the mean OD values and the standard deviations was generated.

The percentages of inhibition, expressed referring to the OD value obtained from the wells with the negative serum as 100%, were presented in Tables 5 and 6.

### Generation of models of the pestivirus E2 proteins

The models of the E2 proteins of CSFV strain Alfort/187 and BVDV strain NADL were prepared with AlphaFold2" of accessible use (58). The figures were prepared using the PyMOL Molecular Graphics System, version 2.5.7 (Schrödinger LLC), and the electrostatic potential was calculated using the APBS electrostatic plugin.

### Preparation of graphs

The calculations of mean values and standard deviations as well as the preparation of the graphs were performed in Excel.

## RESULTS

### Virus-neutralizing activity of monoclonal antibodies

The virus-neutralizing effect of the mAbs was evaluated using a virus neutralization assay with varying concentrations of purified antibodies. The results, presented in Tables 2 and 3, are expressed as the molar concentration of mAb required to neutralize between 30 and 300 TCID_50_ per 50 µL of the corresponding virus. All anti-CSFV mAbs neutralized the CSFV strain Alfort/187 *in vitro*, with antibody concentrations ranging from 1.16 nM (mAb HC/TC63) to 111.11 nM (HC/TC68) (Table 2). The anti-BVDV mAbs BVD/PX18 and BVD/CA82 did not neutralize the BVDV strain NADL (Table 3). In contrast, the remaining antibodies showed virus neutralization at concentrations as low as 69.33 nM for mAbs BVD/CA1, BVD/CA3, and BVD/CA73, and as high as 1,670 nM for mAb BVD/PX14 (Table 3).

**TABLE 2:** Molar concentration of anti-CSFV mAbs that neutralized between 30 and 300 TCID_50_ of CSFV Alfort/187

mAb	Conc. (nM)
HC/TC3	2.31
HC/TC16	27.78
HC/TC18	55.56
HC34	27.78
HC36	2.31
HC37	27.78
HC43	55.56
HC/TC50	1.73
HC/TC54	2.31
HC/TC59	27.78
HC/TC62	9.24
HC/TC63	1.16
HC/TC64	2.31
HC/TC65	13.87
HC/TC68	111.11

**TABLE 3:** Molar concentration of anti-BVDV mAbs that neutralized between 30 and 300 TCID_50_ of BVDV NADL

mAb	Conc.^*a*^ (nM)
BVD/PX101	208
BVD/PX8	1,110
BVD/PX14	1,670
BVD/PX18	Negative^*b*^
BVD/CA1	<69.33
BVD/CA3	<69.33
BVD/CA34	555.56
BVD/CA73	<69.33
BVD/CA82	Negative^*b*^
BVD/CT3	208
BVD/CT6	833.33

^*a*^ Conc. (concentration).

^*b*^ Negative indicates lack of virus-neutralizing activity.

### Reactivity of the E2-specific mAbs with different CSFV, BVDV-1, and BVDV-2 strains

The reaction pattern of the E2-specific mAbs with various CSFV genotypes (gt), BVDV-1, and BVDV-2 strains was analyzed after infection of cells with the corresponding pestiviruses (Table 4). The mAbs (named HC/TC and HC/C), which were generated against the CSFV gt 1.1. strain Alfort/187 (CSF0902), were all tested positive on cells infected with this virus and showed no reactivity with the applied BVDV-1 (NADL and CP7) or BVDV-2 strains (CS8644 and 134/102). Seven mAbs (HC/TC18, HC/TC50, HC/TC59, HC/TC62, HC/C34, HC/C37, and HC/C43) detected all tested CSFV genotypes. In comparison, the mAbs HC/TC3, HC/TC16, HC/TC63, and HC/TC68 recognized most, but not all, of those isolates. The mAbs HC/TC54, HC/TC64, HC/TC65, and HC/C36 are specific for the CSFV strain Alfort/187, which was used for the generation of the corresponding hybridoma cell lines (Table 4).

**TABLE 4:** Reaction pattern of the E2-specific antibodies with various CSFV and BVDV isolates^*a*^

	HC/TC				HC/C			HC/TC							HC/C	BVD/CA					BVD/PX				BVD/CT	
	18	50	59	62	34	37	43	3	16	68	63	54	64	65	36	34	3	73	80	82	101	8	14	18	3	6
CSF0902 (gt 1.1)	+	+	+	+	+	+	+	+	+	+	+	+	+	+	+	-	-	-	-	-	-	-	-	-	-	-
CSF0929 (gt 1.2)	+	+	+	+	+	+	+	+	+	+	+	-	-	-	-	-	-	-	-	-	-	-	-	-	-	-
CSF0650 (gt 1.3)	+	+	+	+	+	+	+	+	+	+	-	-	-	-	-	-	-	-	-	-	-	-	-	-	-	-
CSF0705 (gt 1.4)	+	+	+	+	+	+	+	+	+	-	+	-	-	-	-	-	-	-	-	-	-	-	-	-	-	-
CSF1048 (gt 2.1)	+	+	+	+	+	+	+	+	-	-	-	-	-	-	-	-	-	-	-	-	-	-	-	-	-	-
CSF0573 (gt 2.2)	+	+	+	+	+	+	+	+	+	+	-	-	-	-	-	-	-	-	-	-	-	-	-	-	-	-
CSF0104 (gt 2.3)	+	+	+	+	+	+	+	-	+	+	-	-	-	-	-	-	-	-	-	-	-	-	-	-	-	-
CSF0410 (gt 3.1)	+	+	+	+	+	+	+	+	+	-	-	-	-	-	-	-	-	-	-	-	-	-	-	-	-	-
CSF0309 (gt 3.4)	+	+	+	+	+	+	+	+	+	+	-	-	-	-	-	-	-	-	-	-	-	-	-	-	-	-
BVDV-1 NADL	-	-	-	-	-	-	-	-	-	-	-	-	-	-	-	+	+	+	+	+	+	+	+	+	+	+
BVDV-1 CP7	-	-	-	-	-	-	-	-	-	-	-	-	-	-	-	+	+	+	+	+	+	-	-	-	-	-
BVDV-2 CS8644	-	-	-	-	-	-	-	-	-	-	-	-	-	-	-	+	-	-	-	-	-	-	-	-	-	-
BVDV-2 134/102	-	-	-	-	-	-	-	-	-	-	-	-	-	-	-	+	-	-	-	-	-	-	-	-	-	-

^*a*^ (+) or (-) indicate a positive or negative reaction with the corresponding virus.

The mAbs, which were generated against the BVDV-1a strain NADL, do not cross-react with the tested CSFV strains. With the exception of the mAbs BVD/PX8, BVD/PX14, BVD/PX18, BVD/CT3, and BVD/CT6, the mAbs detect BVDV-1a NADL and BVDV-1b CP7. Only the mAb BVD/CA34 recognized the BVDV-2 strains in addition to the BVDV-1 strains (Table 4).

### Expression and purification of the recombinant CSFV and BVDV glycoprotein E2 in mammalian cells

The expression of glycoproteins in mammalian cells ensures their correct folding, with crucial posttranslational modifications, such as disulfide bond formation and glycosylations in the endoplasmic reticulum. Misfolded proteins are retained intracellularly (59) and degraded. In this study, high yields of highly pure ectodomains of CSFV and BVDV E2, as well as chimeric proteins, were produced using a vector optimized for mammalian cell expression (55). This cost-efficient methodology has previously been used to express envelope glycoproteins of arenaviruses (54), coronaviruses (60), and orthobunyaviruses (57).

**Fig 1:**
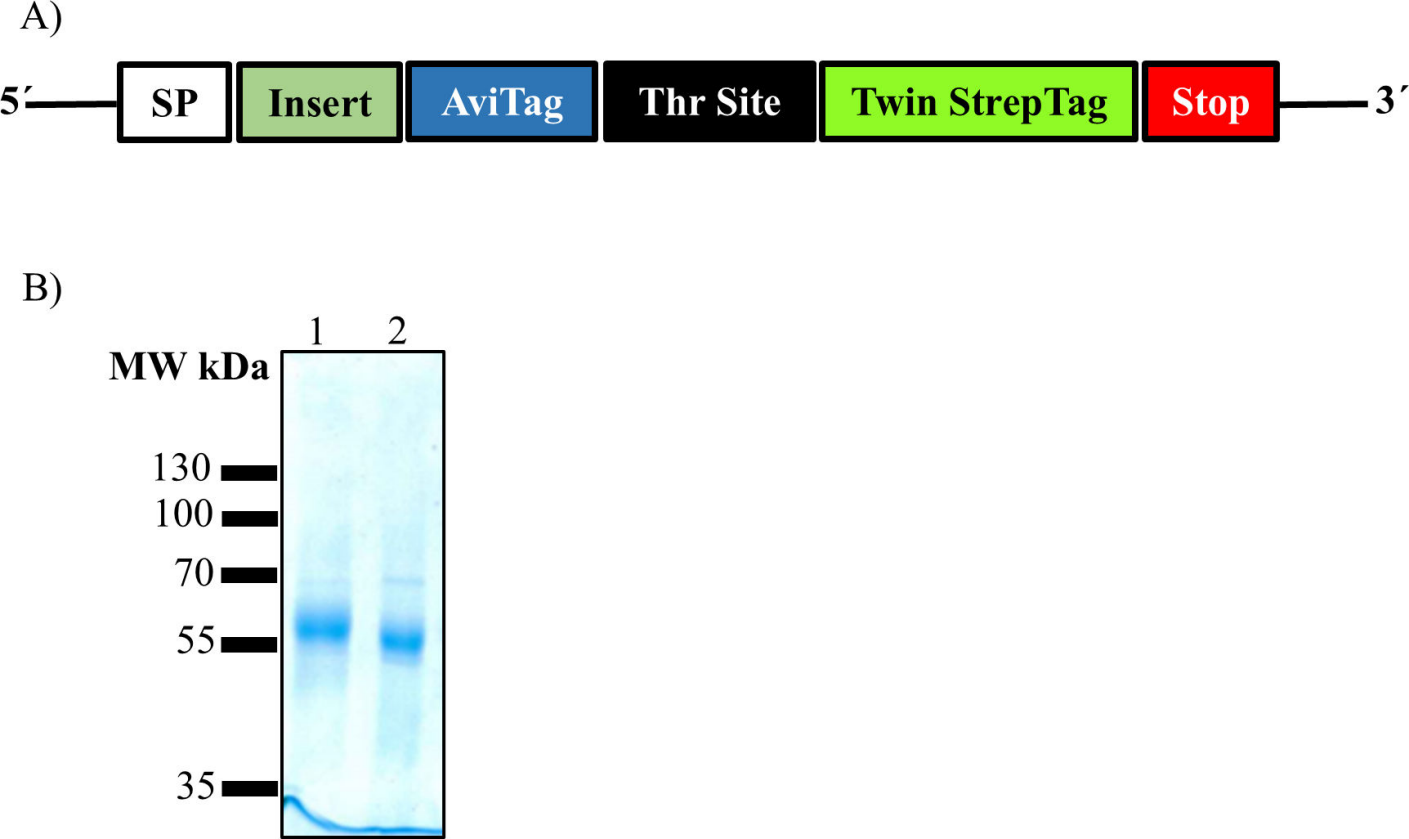
Schematic representation of the construct for recombinant protein production and analysis of the purified CSFV and BVDV E2 proteins by SDS-PAGE. (**A**) The ectodomain of the corresponding E2 protein (insert) was expressed downstream of a signal peptide (SP) followed by a biotinylation tag (AviTag), a thrombin cleavage site (Thr Site), and a StrepTag (Twin StrepTag). The protein translation was terminated by a stop codon (Stop). (**B**) The purified proteins CSFV E2 (lane 1) and BVDV E2 (lane 2) were analyzed by SDS-PAGE under reducing conditions and were stained.

The proteins were flanked by a signal peptide at the amino terminus to direct them to the secretory pathway and by a twin StrepTag at the carboxyl terminus for affinity chromatography purification (Fig. 1A). The ectodomains of CSFV and BVDV E2 glycoproteins were isolated from the supernatant of transiently transfected HEK293T cells, and the quality of the purified material was evaluated by SDS-PAGE and Coomassie staining (Fig. 1B). Although the calculated molecular weight of both proteins is approximately 45 kDa, they migrated at around ~55 kDa (Fig. 1B). The purity of both protein preparations was above 90% with no degradation products observed.

### Reactivity of the monoclonal antibodies with CSFV and BVDV E2 glycoproteins

**Fig 2:**
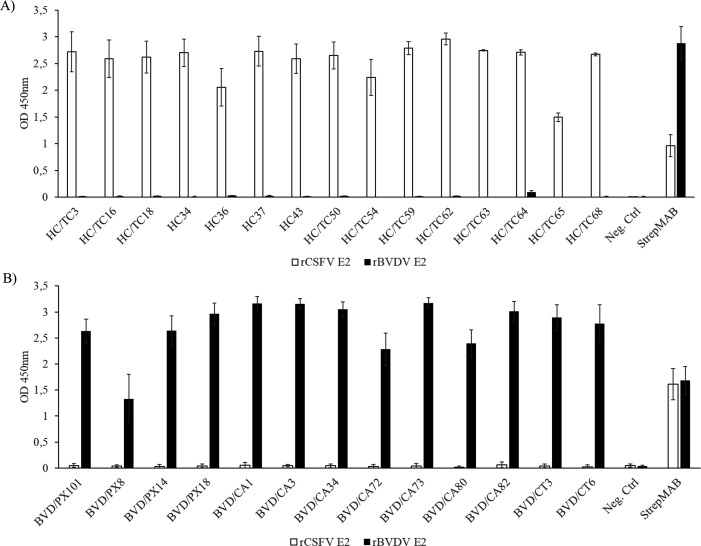
Reactivity of the mAbs with the recombinant CSFV and BVDV E2 glycoproteins. Each panel of mAbs, CSFV mAbs (**A**), and BVDV mAbs (**B**) was tested in an indirect ELISA with both glycoproteins. The results are the mean values of three independent experiments, and the errors bars represent the standard deviations.

The reactivity of the corresponding mAbs was assessed using an indirect ELISA with affinity-purified E2 glycoproteins. All antibodies showed strong reactivity with the recombinant E2, with OD values above 1. The anti-CSFV mAbs (Fig. 2A) and anti-BVDV mAbs (Fig. 2B) did not exhibit evident cross-reactivity with BVDV NADL E2 and CSFV Alfort/187 E2, respectively. An increased OD value was observed in the reaction of the anti-StrepTag control antibody with the BVDV E2 antigen when the anti-CSFV mAbs were tested.

### Blocking of binding of monoclonal antibodies by sera from seropositive animals

**Fig 3:**
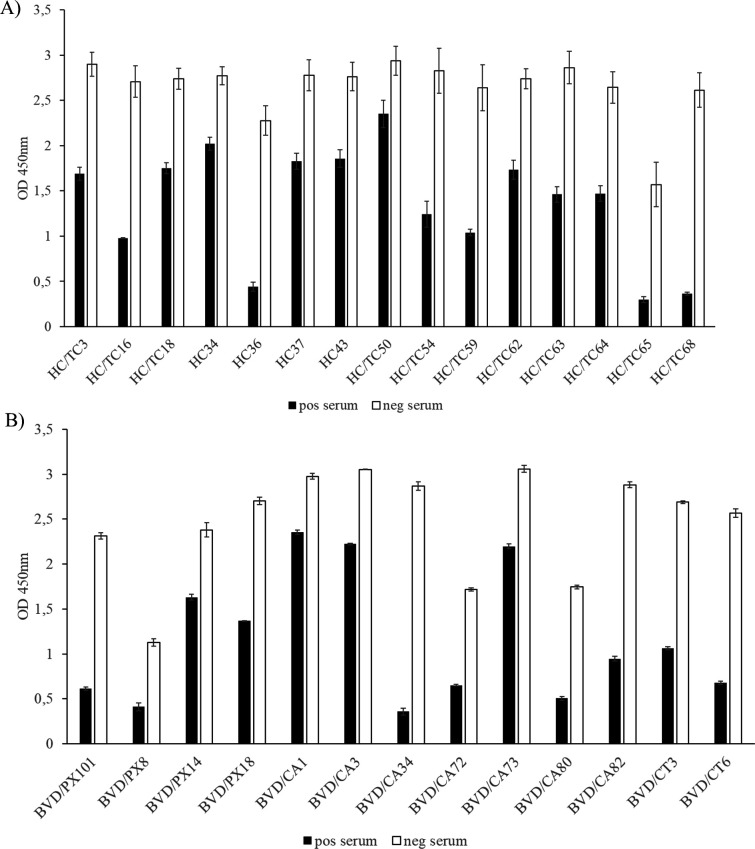
Blocking of binding of mAbs by sera containing antibodies against CSFV or BVDV. The blocking effect of a positive serum on the reaction of CSFV mAbs (**A**) and BVDV mAbs (**B**) with their respective proteins was tested in blocking ELISA. The anti-CSFV mAbs were evaluated with a pig serum containing antibodies against CSFV Alfort/187 and a commercial pig serum, which was tested negative for antibodies against CSFV. The anti-BVDV mAbs were tested with a bovine serum containing antibodies against BVDV NADL and fetal calf serum that was tested negative for antibodies against BVDV. The results are the mean values of three independent experiments for the anti-CSFV mAbs and three wells of one experiment for the anti-BVDV mAbs. The error bars represent the standard deviation.

To determine if the epitopes recognized by the panel of murine mAbs are shared by antibodies generated during the course of an infection in the natural host, a blocking ELISA was performed using a serum from a pig after infection with CSFV Alfort/187 (Fig. 3A; Table 5) or a serum from a cattle after infection with BVDV NADL (Fig. 3B; Table 6).

**TABLE 5:** Percent of inhibition of reactivity of anti-CSFV mAbs by a CSFV antibody-positive swine serum

Group	mAb	% inhibition
1	HC/TC68	86.04
	HC/TC65	81.04
	HC36	80.59
2	HC/TC16	63.9
	HC/TC59	60.54
	HC/TC54	56.02
3	HC/TC63	48.97
	HC/TC64	44.39
	HC/TC3	41.7
4	HC/TC62	36.62
	HC/TC18	36.09
	HC37	34.13
	HC43	32.78
5	HC34	27.11
	HC/TC50	19.96

**TABLE 6:** Percent of inhibition of reactivity of anti-BVDV mAbs by a BVDV antibody-positive bovine serum

Group	mAb	% inhibition
1	BVD/CA34	87.51
2	BVD/PX101	73.5
	BVD/CT6	73.66
	BVD/CA80	71.04
	BVD/CA82	67.14
3	BVD/PX8	63.51
	BVD/CA72	62.13
	BVD/CT3	60.40
4	BVD/PX18	49.44
	BVD/PX14	31.48
	BVD/CA73	28.14
	BVD/CA3	27.18
	BVD/CA1	20.89

The degree of inhibition of the anti-CSFV E2 mAbs by the positive porcine serum ranged from 86.4% to 20.0% (Fig. 3A; Table 5). The reactivity of mAbs HC/TC68, HC/TC65, and HC36 was inhibited from 86.0% to 80.6%, followed by mAbs HC/TC16, HC/TC59, and HC/TC54 (63.9% to 56.0%); HC/TC63, HC/TC64, and HC/TC3 (49.0% to 41.7 %); HC/TC62, HC/TC18, HC37, and HC43 (36.6% to 32.8%); and HC34 and HC/TC50 with 27.1% and 19.96% inhibition, respectively.

Based on the degree of inhibition by BVDV antibody-positive bovine serum, the anti-BVDV E2-specific mAbs were categorized into four groups (Fig. 3B; Table 6). The strongest inhibitory effect was observed for mAb BVD/CA34 (87.5%). The mAbs BVD/PX101, BVD/CT6, BVD/CA80, and BVD/CA82 were inhibited from 73.7% to 67.1%, whereas mAbs BVD/CA72, BVD/CT3, and BVD/PX8, and mAbs BVD/PX18, BVD/PX14, BVD/CA73, BVD/CA3, and BVD/CA1 were inhibited from 63.5% to 60.4% and 49.4% to 20.9%, respectively.

### Effect of low-pH treatment of glycoprotein E2 on the reactivity of monoclonal antibodies

**Fig 4:**
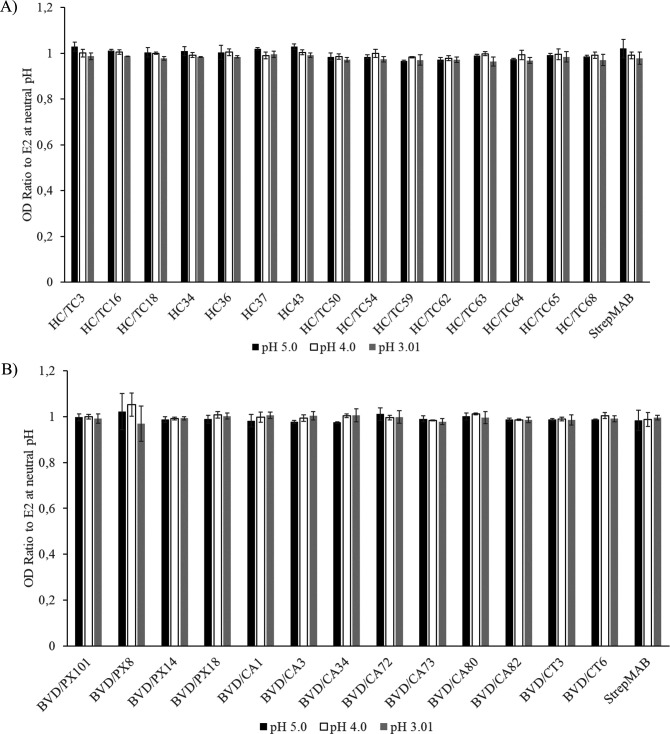
Reactivity of mAbs with the recombinant E2 upon low-pH treatment. The reactions of CSFV E2- (**A**) or BVDV E2-specific mAbs (**B**) were tested in an indirect ELISA with the corresponding protein that was previously treated at the indicated pH values. The results were expressed as a ratio to the reactions of the mAbs at neutral pH. The solid bars represent the mean values of three independent experiments, and the error bars correspond to the standard deviations.

Given that pH can induce conformational changes in proteins, we assessed whether the epitopes recognized by this panel of mAbs would be sensitive to treatment at low-pH values. The E2 protein immobilized on the ELISA plate was treated with low pH before being tested with the mAbs in an indirect ELISA. The reaction of the control anti-StrepTag antibody was not affected, indicating that the applied method did not significantly impact protein concentrations (Fig. 4). The low-pH treatment did not influence the reactivity of either the anti-CSFV or the anti-BVDV mAbs (Fig. 4A and B).

### Reactivity of monoclonal antibodies with denatured glycoprotein E2

**Fig 5:**
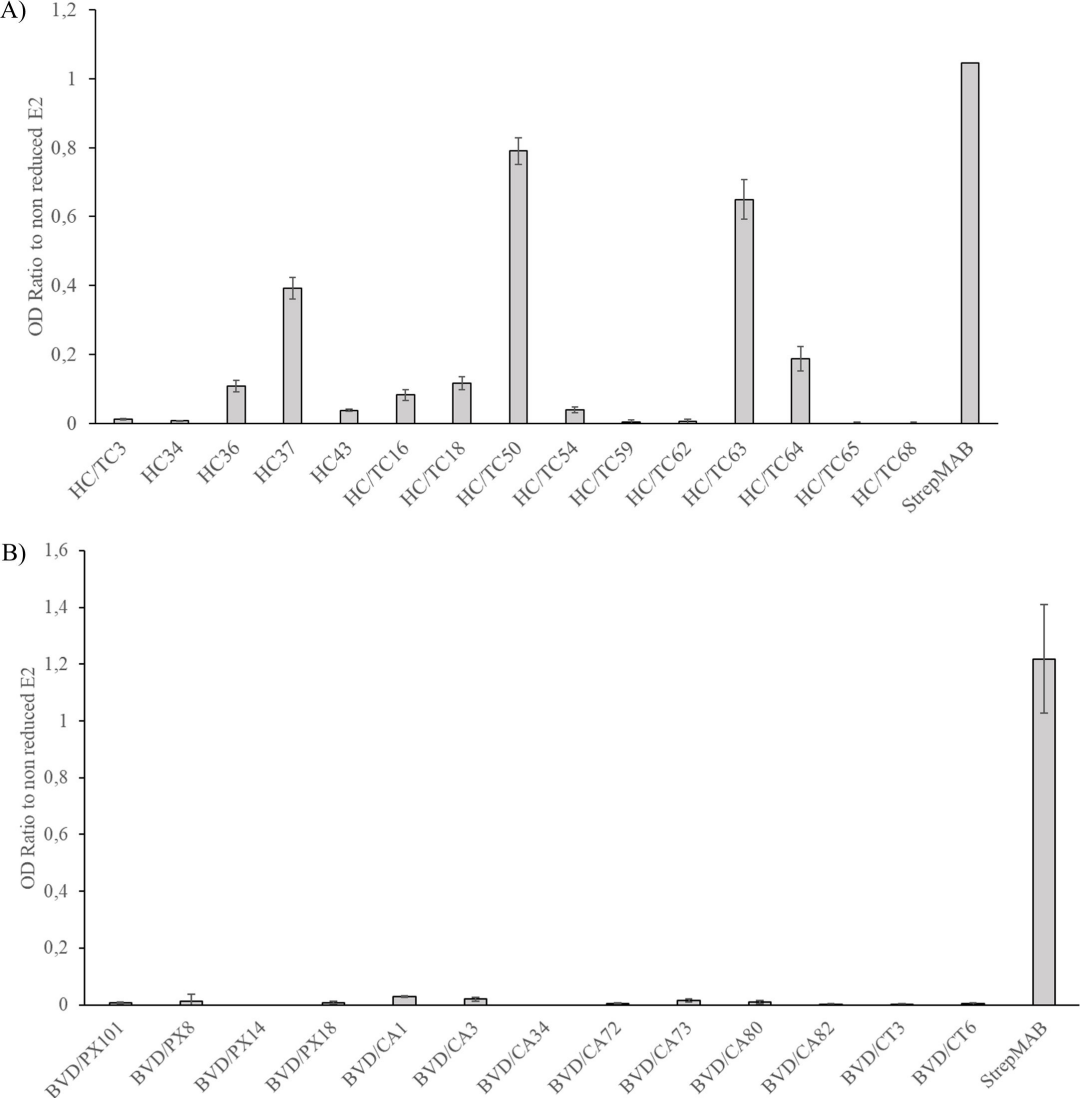
Reactivity of the mAbs after protein denaturation. The recombinant CSFV E2 (**A**) or BVDV E2 (**B**) proteins were denatured under reducing conditions, and the reactions of the corresponding mAbs with the treated and untreated protein were assessed in an indirect ELISA. The ratios of the reactions of the individual mAbs with the denatured protein to the reactions with the untreated protein were calculated. The results represent the mean value of three independent experiments, and the error bars correspond to the standard deviations.

Disulfide bonds are essential for the tertiary structure of proteins; thus, we evaluated their role in forming the epitopes recognized by the mAbs characterized in this study. To achieve this, the E2 glycoprotein was treated with a disulfide bond-reducing agent before being coated onto the ELISA plate. The cysteines were then alkylated to prevent the reshuffling of disulfide bonds. Finally, the reactivity of the mAbs was evaluated using an indirect ELISA (Fig. 5).

The anti-CSFV mAb HC/TC50 retained 80% reactivity with the denatured E2 compared to the non-treated protein, while mAbs HC/TC63 and HC37 reacted with the denatured protein at approximately 60% and 40% of the reaction with the non-treated protein, respectively. The other CSFV-specific mAbs showed a negligible reactivity with the denatured CSFV E2 protein (Fig. 5A). In contrast, the reactivity of all anti-BVDV mAbs with the BVDV E2 protein was completely abolished upon protein denaturation (Fig. 5B).

### Assessment of the binding strength of the mAbs

**Fig 6:**
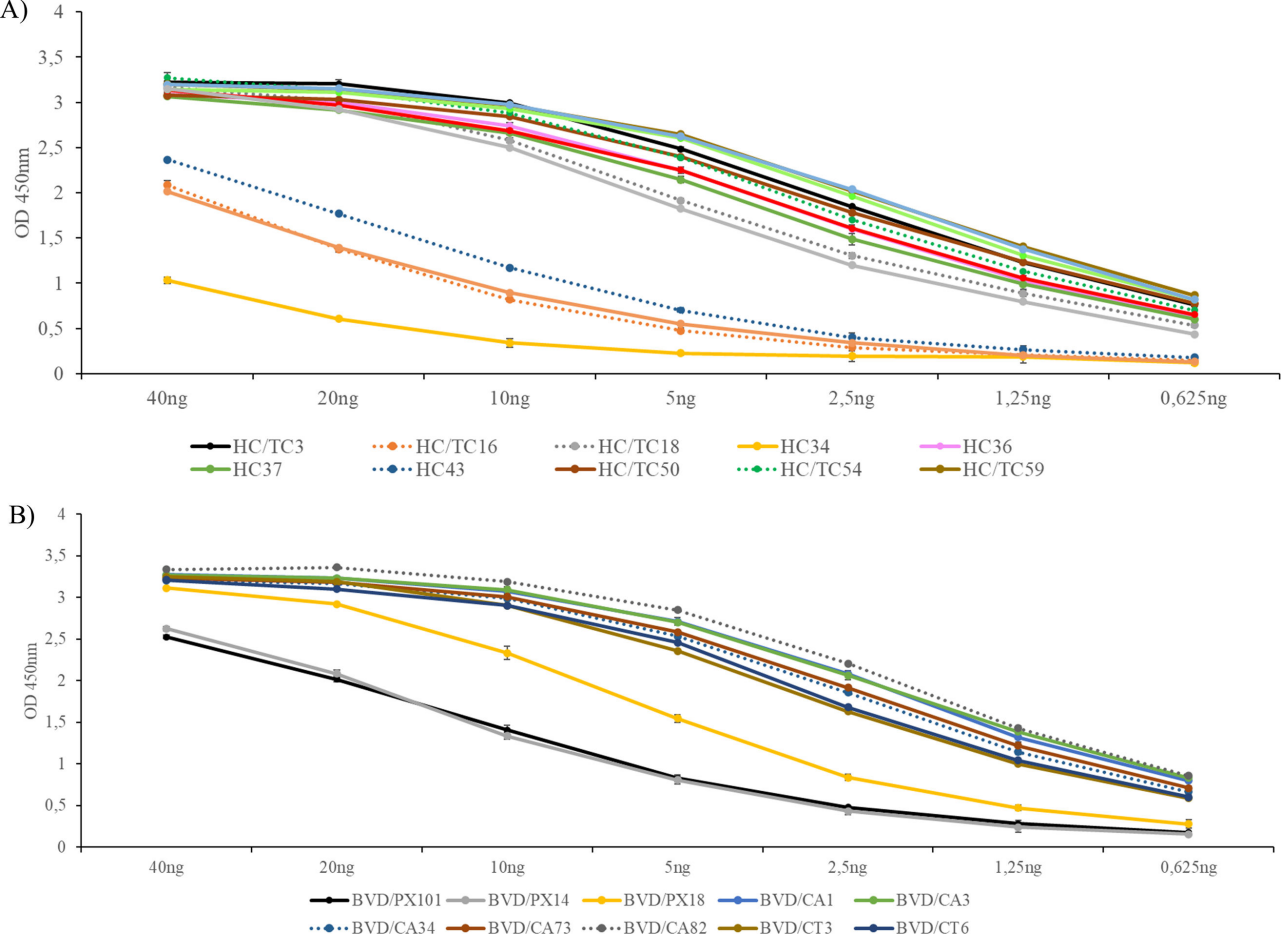
Assessment of the binding strength of the mAbs to the E2 protein of CSFV and BVDV. The reaction of anti-CSFV (**A**) or anti-BVDV (**B**) mAbs with the corresponding CSFV E2 (**A**) and BVDV E2 (**B**) proteins was tested at the indicated amounts in an indirect ELISA. The results represent the mean of the OD 450 nm values of three wells determined in one experiment.

The binding strength of the mAbs to either CSFV E2 or BVDV E2 was semi-quantitatively estimated using an indirect ELISA with defined concentrations of purified mAbs (Fig. 6). All the CSFV mAbs showed high OD 450 nm values, ranging from 1 for HC34 to 3.3 for HC/TC54 when 40 ng of the antibody was tested (Fig. 6A). The strongest reactions were observed for the mAbs HC/TC3, HC/TC18, HC36, HC37, HC/TC50, HC/TC54, HC/TC59, HC/TC62, HC/TC63, HC/TC64, and HC/TC68 with OD 450 nm values above 2.5 using 10 ng mAb. These antibodies produced OD 450 nm signals between 0.5 and 1 when 0.652 ng antibody was tested, except mAb HC/TC68, which showed slightly lower values (0.4). A second group of antibodies (mAbs HC/TC16, HC43, HC/TC65) reacted more weakly compared to the aforementioned mAbs. The lowest OD 450 nm signals were observed with mAb HC34.

The anti-BVDV mAbs (Fig. 6B), except for mAbs BVD/PX101 and BVD/PX14, showed OD 450 nm values above 2.5 when 20 ng antibody was tested. The mAbs BVD/CA1, BVD/CA3, BVD/CA34, BVD/CA73, BVD/CA82, BVD/CT3, and BVD/CT6 exhibited the highest OD 450 nm signals, which remained between 0.5 and 1 even at the lowest antibody concentration tested (0.652 ng). The mAb BVD/PX18 reacted similarly to the abovementioned mAbs at 40 ng, but at lower amounts, its signals declined below those of that group. The lowest signals among the BVDV mAbs were detected with mAbs BVD/PX101 and BVD/PX14. Nevertheless, 5 ng of these mAbs still reacted strongly, with OD 450 nm values between 0.5 and 1.

### Generation of the recombinant chimeric E2 glycoproteins from mammalian cells

**Fig 7:**
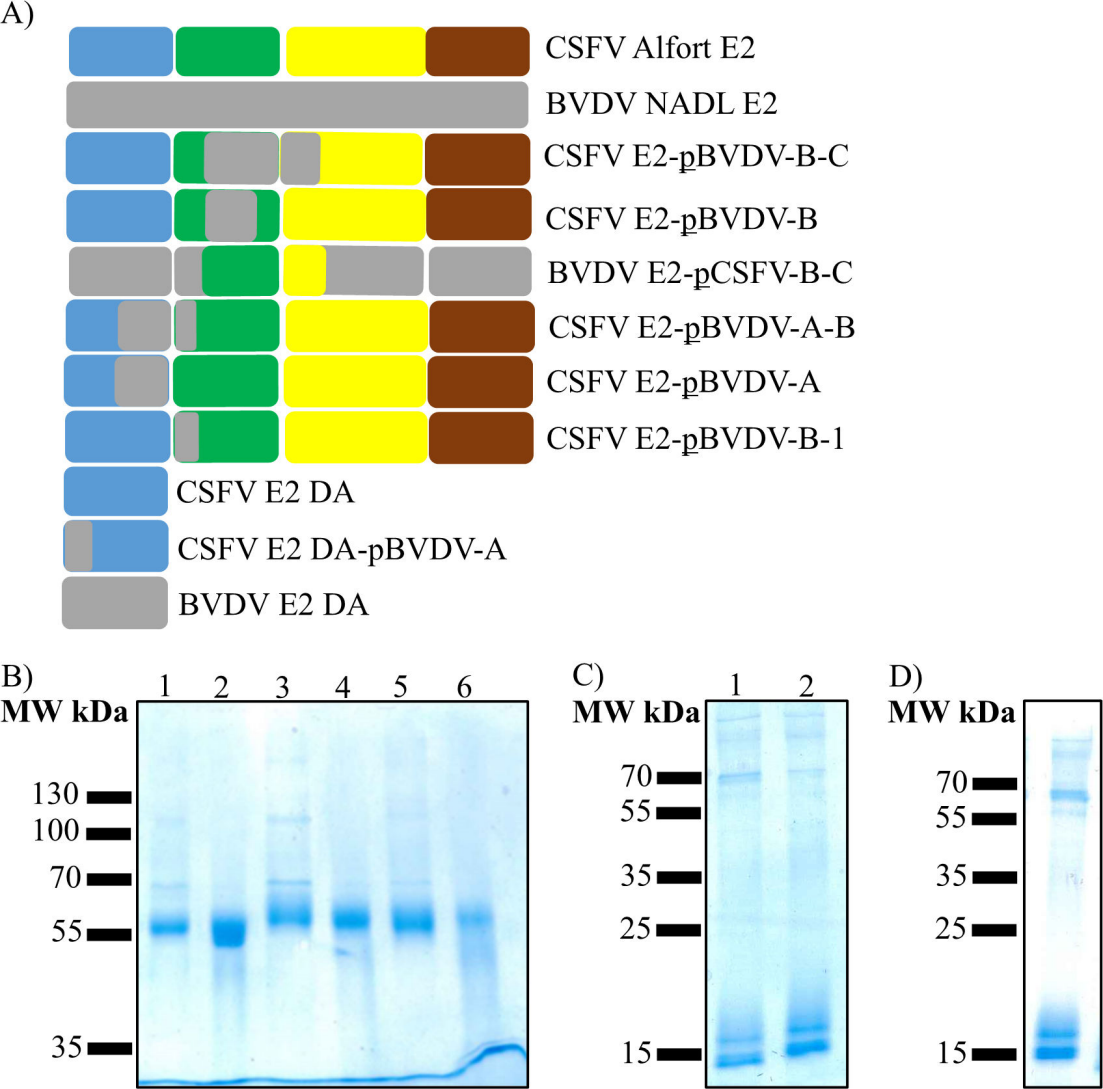
Schematic representation and analysis of the recombinant proteins generated to perform the epitope mapping of the mAbs. In the schematics (**A**), the blocks representing BVDV sequences (NADL) are shown in gray, whereas the CSFV-specific blocks (Alfort/187) are in blue, green, yellow, and brown, corresponding to the domain A (DA), domain B (DB), domain C (DC), and domain (DD), respectively (34). The “p” included in the constructs indicates “partial.” Hence, the constructs are explained as follows: CSFV E2-pBVDV-B-C (ectodomain of CSFV E2 with partial sequence of BVDV in domains B and C); CSFV E2-pBVDV-B (ectodomain of CSFV E2 with partial sequence of BVDV in domain B); BVDV E2-pCSFV-B-C (ectodomain of BVDV E2 with partial sequence of CSFV in domains B and C); CSFV E2-pBVDV-A-B (ectodomain of CSFV E2 with partial sequence of BVDV in domains A and B); CSFV E2-pBVDV-A (ectodomain of CSFV E2 with partial sequence of BVDV in domain A); CSFV E2-pBVDV-B-1 (ectodomain of CSFV E2 with partial sequence of BVDV in domain B); CSFV E2 DA (domain A of CSFV E2); CSFV E2 DA-pBVDV-A (domain A of CSFV E2 with partial sequence of BVDV in domain A); BVDV E2 DA (domain A of BVDV E2). (**B–D**) The purified proteins of constructs CSFV E2-pBVDV-B-C; CSFV E2-pBVDV-B; BVDV E2-pCSFV-B-C; CSFV E2-pBVDV-A-B; CSFV E2-pBVDV-A; CSFV E2-pBVDV-B-1 (B, lanes 1 to 6, respectively); CSFV E2 DA; CSFV E2 DA-pBVDV-A (C, lanes 1 and 2, respectively); and BVDV E2 DA (**D**) were analyzed on SDS-PAGE under reducing conditions and were stained with Coomassie blue, respectively.

The lack of cross-reactivity of the anti-CSFV and anti-BVDV mAbs against the E2 protein of BVDV NADL and the E2 of CSFV Alfort/187, respectively, as well as the conformational nature of most of the epitopes, suggested the use of chimeric recombinant E2 proteins as a plausible strategy for epitope mapping. The use of chimeric proteins preserves the overall conformation, ensuring that a lack of reaction does not result from misfolding of the protein used. As shown in Figure 7A, besides the constructs expressing the full length of CSFV E2 and BVDV E2 ectodomains, six chimeric recombinant proteins of the full-length E2 protein ectodomain, the CSFV domain A, the BVDV domain A, and one chimeric domain A were designed and purified by affinity chromatography. The quality of the preparations was evaluated by SDS-PAGE and Coomassie staining under reducing conditions (Fig. 7B through D). The purity of the preparations was above 90%, and all full-length chimeric proteins migrated at around 55 kDa, similar to the CSFV E2 and BVDV E2 ectodomains, with slight differences among them. The signal observed in the lane of the CSFV E2-pBVDV-B-1 protein was slightly weaker compared to the signals of the other proteins. The preparations of the CSFV E2 subdomain A (CSFV E2 DA) and its chimera (CSFV E2 DA-pBVDV-A) (Fig. 7C), as well as the BVDV E2 subdomain A (BVDV E2 DA) (Fig. 7D), migrated as a double band of approximately the expected molecular weight of 15 kDa.

### Reactivity of the monoclonal antibodies with chimeric E2 glycoproteins

**Fig 8:**
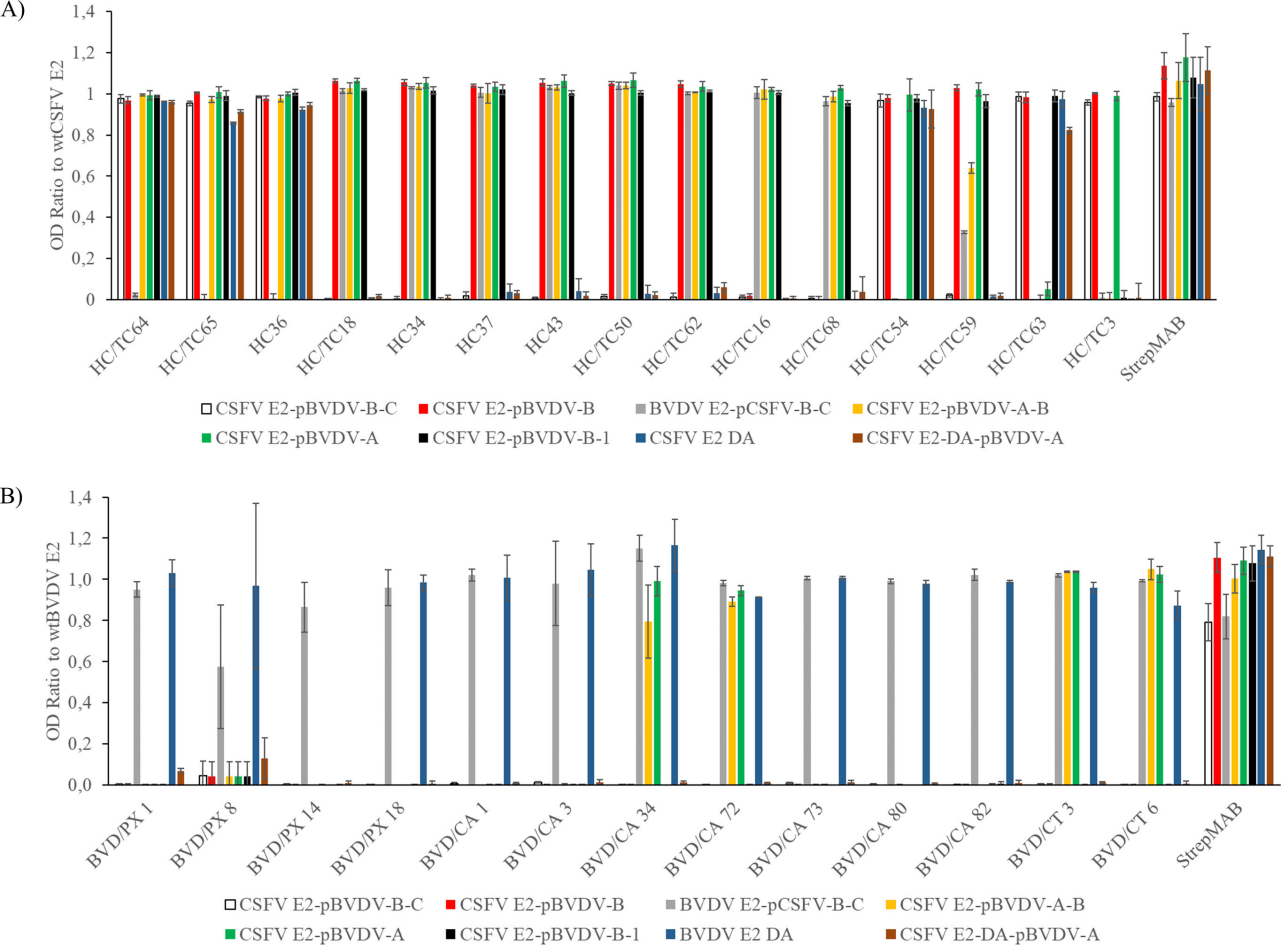
Reaction of the mAbs with the recombinant chimeric E2 proteins. The anti-CSFV (**A**) and anti-BVDV (**B**) mAbs were tested with the chimeric E2 proteins (described in Fig. 7) in an indirect ELISA. The results are expressed as the OD 450 nm ratio to wt CSFV E2 (**A**) or wt BVDV E2 (**B**), and represent the mean values of three independent experiments. The error bars correspond to the standard deviation.

**Fig 9:**
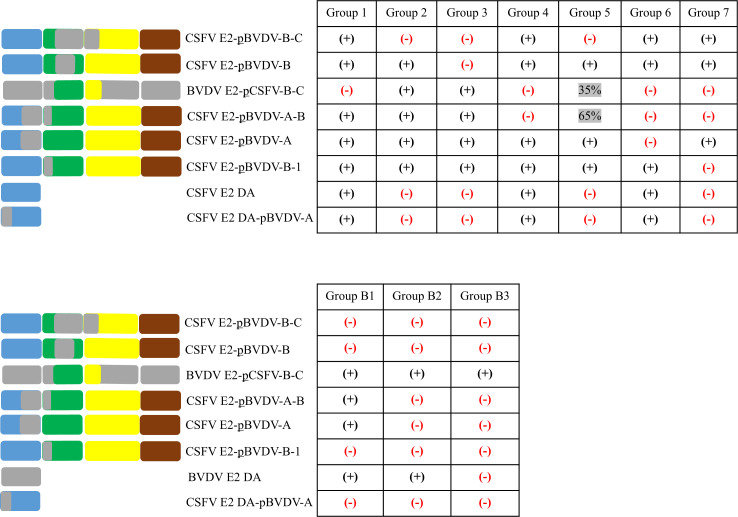
Summary of the results of the reactivity of the mAbs with the chimeric proteins shown in Figure 8. The mAbs are grouped according to their reactivity pattern. The groups of the anti-CSFV mAbs are designated group 1 to group 7 (top), whereas the groups of anti-BVDV mAbs are named group B1 to group B3 (bottom). The BVDV E2 domains are displayed in gray, whereas the CSFV E2 domains DA, DB, DC, and DD are depicted in blue, green, yellow, and brown, respectively. (+), positive reaction; (–), negative reaction.

The effect of mutations was estimated quantitatively using an indirect ELISA and was expressed as the OD 450 nm ratio of the mutated/chimeric E2 protein to the OD 450 nm of the wt CSFV Alfort E2 (Fig. 8A) or wt BVDV NADL E2 (Fig. 8B). The anti-CSFV mAbs showed varied reactivity with the chimeric proteins (Fig. 8A) and were categorized into seven distinct groups based on their reactivity patterns (Fig. 9, upper panel): group 1 (three mAbs: HCTC64, HCTC65, and HC36); group 2 (six mAbs: HC/TC18, HC34, HC37, HC43, HC/TC50, and HC/TC62); group 3 (two mAbs: HC/TC16 and HC/TC68); group 4 (HC/TC54); group 5 (HC/TC59); group 6 (HC/TC63); and group 7 (HC/TC3). Similarly, the panel of anti-BVDV mAbs was tested. The OD ratios were calculated relative to the BVDV E2 protein, with the construct encoding the CSFV E2 domain A (CSFV E2 DA) substituted by the recombinant BVDV E2 domain A (BVDV E2 DA) (Fig. 8B). All anti-BVDV mAbs, except BVD/PX14, reacted with the recombinant BVDV E2 domain A (BVDV E2 DA) and, without exception, with BVDV E2-pCSFV-B-C (Fig. 9, lower panel). These mAbs showed three reactivity patterns, resulting in their categorization into the three groups B1 (BVD/CA34, BVD/CA72, BVD/CT3, and BVD/CT6); B2 (BVD/PX1, BVD/PX8, BVD/PX18, BVD/CA1, BVD/CA3, BVD/CA73, BVD/CA80, and BVD/CA82); and B3 (BVD/PX14).

### Localization of the epitopes on the structure of the glycoprotein E2

**Fig 10:**
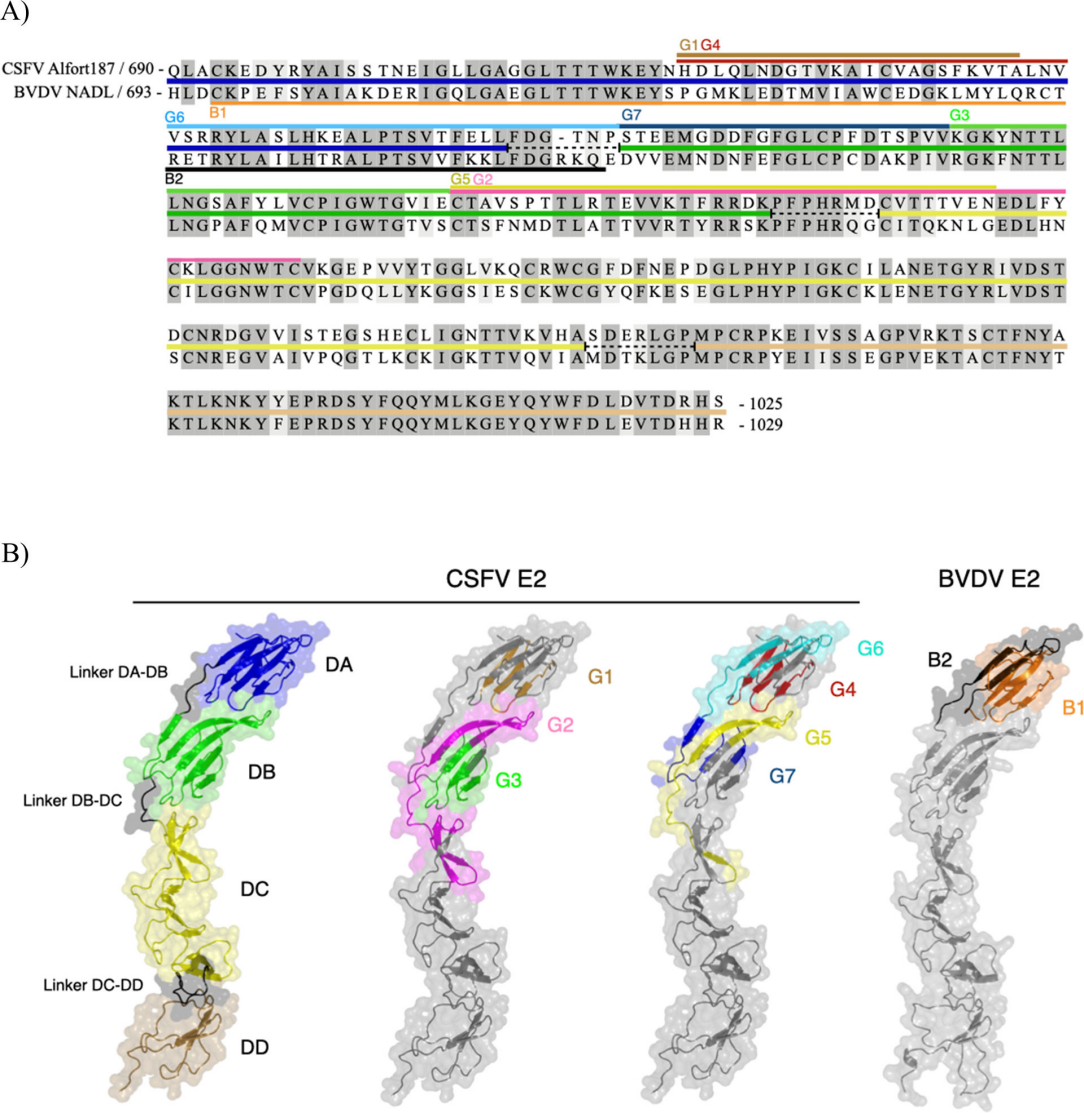
Structural location of E2 epitopes. (**A**) Pair-to-pair alignment of the amino acid sequences of CSFV Alfort/187 E2 and BVDV NADL E2. Domains DA (blue), DB (green), DC (yellow), and DD (brown) are displayed as colored lines between the two sequences in the alignment. Interdomain linkers are depicted as black dashed lines. Epitopes G1 to G7 recognized by the CSFV-specific mAbs and epitopes B1 and B2 recognized by the BVDV-specific mAbs are indicated by straight lines above and below the aligned sequences, and colored as in the cartoon representation shown in panel B, respectively. (**B**) Cartoon representation and translucent molecular surface of the AlphaFold-generated structure models of CSFV Alfort/187 E2 and BVDV NADL E2 proteins. From left to right, CSFV domain A (DA), domain B (DB), domain C (DC), and domain D (DD), as well as the three interdomain linkers, are colored blue, green, yellow, brown, and black, respectively. CSFV epitopes G1, G2, G3 are colored sand, fuchsia, and green, respectively. CSFV epitopes G4, G5, G6, and G7 are colored red, yellow, turquoise, and blue, respectively. BVDV epitopes B1 and B2 are colored black and orange, respectively.

Following the identification of epitope-reactive regions with the different mAbs, our next objective was to visualize these epitopes in the 3D structure of glycoprotein E2. Because the structures of CSFV Alfort/187 E2 and BVDV NADL E2 are not available, the published structure of BVDV strain PE515 E2 (61) was utilized as a control reference, and high-fidelity AlphaFold models of the ectodomains for both CSFV E2 and BVDV E2 proteins were generated (Fig. 10B).

The epitopes are highlighted in different colors assigned to the corresponding groups in the amino acid sequences (Fig. 10A) as well as in the models (Fig. 10B). Anti-CSFV E2 mAbs from groups 1, 4, and 6 targeted epitopes within domain A. The epitopes of groups G1 and G4 are located in the middle of DA, while the epitope of group G6 is located in the carboxyl-terminal half of DA. The epitope of the group 4 mAb requires three additional residues (LNV sequence) at the carboxyl terminus of the sequence. The epitope recognized by the group 7 mAb is located in a region encompassing a part of the DA-DB linker and the amino-terminal part of domain B, while the epitopes targeted by group 3 mAbs are centrally located within DB. The target sequences for mAbs of groups 2 and 5 are positioned at the carboxyl-terminal region of domain B, including the DB-DC linker. The group 5 mAb epitope is found in a shorter segment.

Anti-BVDV mAbs reacting with DA were subdivided into groups B1 and B2, according to their ability to bind to the carboxyl- and amino-terminal halves of this domain, respectively (Fig. 10B). The binding site of mAb BVD/PX14, which exclusively reacts with the construct BVDV E2-pCSFV-B/C, could not be precisely determined based on the reaction pattern. Further constructs are needed to ascertain its binding site.

## DISCUSSION

Monoclonal antibodies targeting envelope glycoproteins have been pivotal in developing assays for differential pestivirus diagnosis (39, 40, 62, 63) and detecting vaccine-induced immune responses (64). The panel of mAbs characterized in our study holds potential use in diagnostics and vaccine development, given their ability to neutralize viruses and their susceptibility to interference by sera from infected animals. A prerequisite for the use of mAbs in diagnostic tests is that they react species specific regardless of the different genotypes within one pestivirus species. The mAbs HC/TC18, HC/TC50, HC/TC59, HC/TC62, HC/C34, HC/C37, and HC/C43 represent a CSFV-specific antibody panel, which detects all tested CSFV genotypes and can therefore be applied for the detection of CSF in diagnostic methods. In comparison to this, only one anti-BVDV mAb (BVD/CA34) was able to detect both BVDV-1 and BVDV-2.

Porcine (65, 66) and murine mAbs that neutralize pestiviruses *in vitro* (53, 67) primarily target the E2 protein. While most mAbs characterized here effectively neutralized CSFV or BVDV, they exhibited varying efficiency. Anti-CSFV mAbs neutralized the virus at concentrations as low as 1.7 nM, whereas anti-BVDV mAbs generally required higher concentrations for neutralization. This discrepancy does not result from reduced mAb activity post-purification or lower affinity, as anti-BVDV mAbs showed stronger signals in indirect ELISA compared to anti-CSFV mAbs at lower concentrations.

It can be speculated that the domain A, to which almost all anti-BVDV mAbs bind, plays a less critical role for the entry of this virus in MDBK cells than it does for CSFV to infect PK15 cells. Epitopes targeted by CSFV-neutralizing mAbs and located in domains A (67), B (53, 67), and C (67) have been reported. These domains are referred to according to the antigenic mapping of E2 (32, 35) as domains D/A and B/C, and they are included in structural domains B and A, respectively. The use of virions for the production of hybridoma cell lines has the great advantage that the viral antigens are presented in a way that certain structural elements, such as quaternary epitopes, are retained. This explains that all anti-BVDV mAbs and the majority of the anti-CSFV mAbs detected conformational epitopes. Similar to previously reported mAbs, their epitopes are located in structural domains A (10) and B (53, 68) of the E2 model. The results of the present study represent an excellent basis for the determination of the key amino acid residues responsible for antibody binding in a future study. Based on the reaction patterns of the mAbs with the different pestiviruses (Table 4) and alignments of the corresponding pestiviral E2 amino acid sequences, site-directed mutagenesis will allow narrowing down the key residues in the antigenic domains of the E2 protein and provide further insights into the E2 antigenic structure of pestiviruses.

The anti-CSFV mAbs HC/TC50, HC/TC63, and HC37, which have strong virus-neutralizing activity, reacted with linear epitopes located at the carboxyl-terminal part of domain B and part of the domain B-domain C linker, or at a sequence encompassing the carboxyl terminus of domain A and the domain A-domain B linker. Linear epitopes of CSFV E2 targeted by mAbs that neutralize virus infection *in vitro* (53) have been identified in domain B (53, 69–71), domain A (67), and the linker between domains A and B (72).

The fact that the majority of the anti-CSFV mAbs detect conformational epitopes makes them potentially useful tools to assess changes in the structure of the E2 glycoprotein during entry, such as those caused by low pH exposure of the viral particles in endosomes (73). The structure of the BVDV E2 protein at neutral (33) and low pH (34) has been solved, revealing that the structural domain A (nomenclature according to reference [34]), located at the amino terminal part of the antigenic domain B (35), became disordered at pH 5 (34). Low-pH treatment of glycoprotein E2 did not affect the reactivity of the mAbs (Fig. 4), which was especially surprising for the domain A-specific mAbs. The observation that the epitopes recognized by these mAbs remained unaffected suggests that the E2 protein can regain, at least to some extent, its original conformation once the pH is restored to neutral. These results might partially explain the observed resistance of pestiviruses to low-pH treatment (74), as the virus-neutralizing activity of these mAbs suggests that their epitopes are relevant sequences for virus–cell interaction.

**Fig 11:**
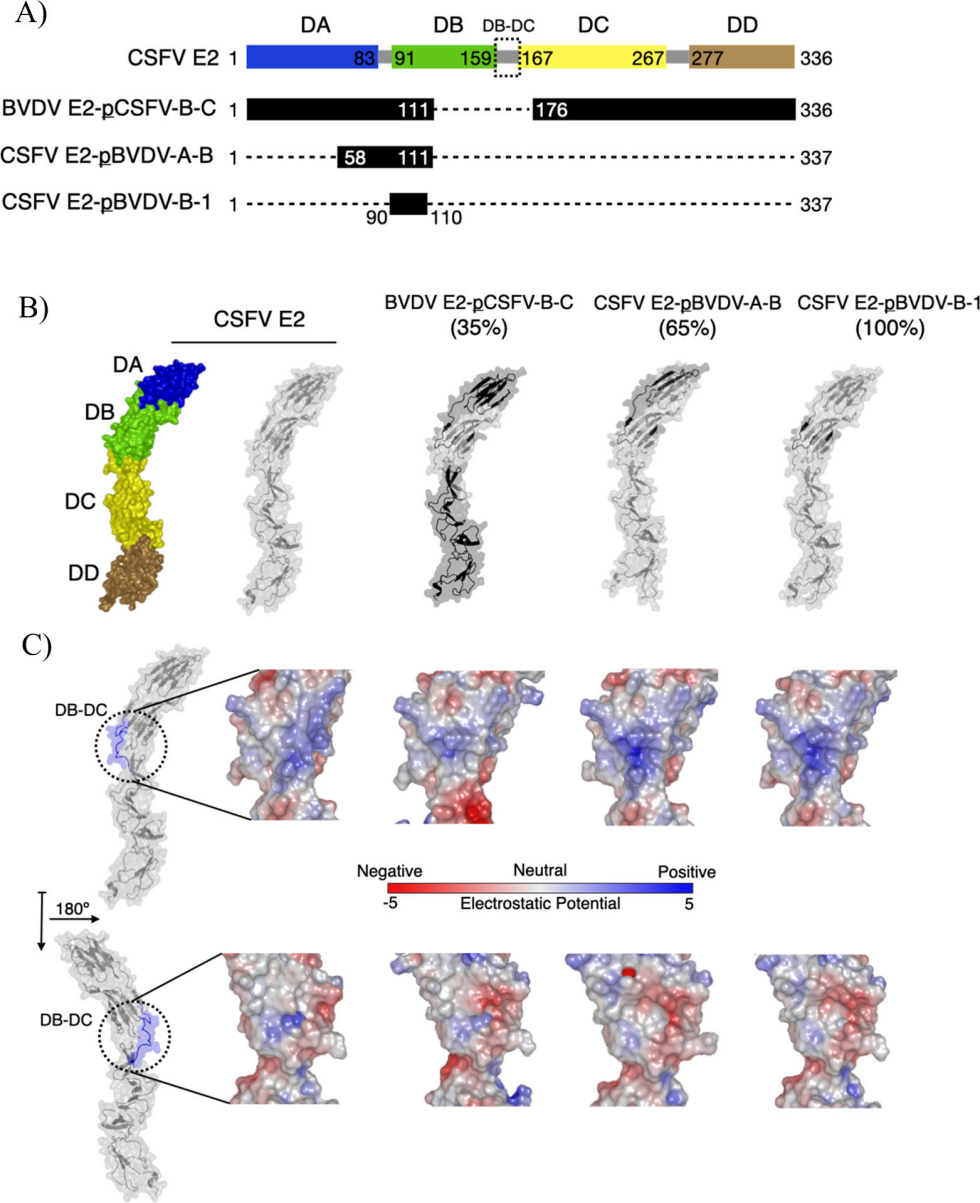
Predicted structural alterations of chimeric E2 proteins on the molecular surface. (**A**) Sequence scheme of the ectodomain of CSFV E2 and the CSFV-BVDV E2 chimeras, highlighting the positions of BVDV E2 insertions as black boxes. The interdomain linkers are displayed in gray, and the DB-BC linker is highlighted with a dotted box. (**B**) AlphaFold-generated models of the ectodomain of CSFV E2 and the chimeric E2 proteins. From left to right: the CSFV E2 molecular surface with domains DA, DB, DC, and DD colored in blue, green, yellow, and brown, respectively, followed by the translucent molecular surface and cartoon representation of CSFV E2 and CSFV-BVDV E2 chimeras. CSFV E2 is shown in gray, and BVDV E2 insertions are shown as black boxes. The percentage of inhibition effect with respect to the wt is shown in parentheses. (**C**) Electrostatic potential of the AlphaFold-generated models displayed on the molecular surfaces as shown in panel B, zoomed at the DB-DC linker region, in two different 180° orientations.

The anti-CSFV and anti-BVDV mAbs were assigned to seven and two groups, respectively, which are highlighted in the models of the corresponding E2 proteins (Fig. 10). The anti-CSFV mAb HC/TC59 exhibited intriguing interactions with the chimeric proteins. The binding site of HC/TC59 (group 5) was pinpointed to the domain B-domain C linker sequence. This was evidenced by the absence of reactivity with the CSFV E2-pBVDV-B-C construct. However, there was a pronounced reduction in reactivity with the BVDV E2-pCSFV-B-C (35%) and CSFV E2-pBVDV-A-B (65%) constructs, despite the absence of amino acid changes in the domain B-domain C linker. These alterations raised the possibility of modifications in the epitope of HC/TC59. Upon comparing the structural model of the chimeric proteins with that of CSFV E2 (see Fig. 11), discernible alterations were identified in the electrostatic surface exposed on the domain B-domain C linker.

This intriguing discovery implies that changes in the linkers connecting domains A and B, or connecting domains C and D, could potentially induce allosteric effects, resulting in a conformational shift in the domain B-domain C linker. The phenomenon of surface rearrangements in viral glycoproteins is not unprecedented. Similar observations have been documented for chikungunya virus (75) and other alphaviruses (76). In these cases, rearrangements were induced either by viral binding to the cell surface or through antiviral-escape mutations. Considering the presence of three interdomain linkers in the pestiviral E2, our findings suggest a plausible mechanism for the virus to alter its surface without resorting to genetic mutations. This insight contributes to our understanding of the dynamic interactions between viral glycoproteins and antibodies, paving the way for potential antiviral interventions.

In summary, (i) the mAbs and the recombinant proteins generated in this study could be used for pestiviral diagnostics development, (ii) the characterization revealed sequences that remained reactive upon low-pH treatment and could be used in structure-assisted design of vaccines, and (iii) this study provides the first indication of conformation interdependence among the linker sequences of the pestivirus E2.

## SUPPLEMENTAL MATERIAL

Supplemental material spectrum.02041-24-s0001.docx Nucleotide sequences of plasmids.

ASM does not own the copyrights to Supplemental Material that may be linked to, or accessed through, an article. The authors have granted ASM a non-exclusive, world-wide license to publish the Supplemental Material files. Please contact the corresponding author directly for reuse.

## References

[1] Ganges L, Crooke HR, Bohórquez JA, Postel A, Sakoda Y, Becher P, Ruggli N. 2020. Classical swine fever virus: the past, present and future. Virus Res 289:198151. doi:10.1016/j.virusres.2020.198151 32898613

[2] Postel A, Smith DB, Becher P. 2021. Proposed update to the taxonomy of pestiviruses: eight additional species within the genus Pestivirus, family Flaviviridae. Viruses 13:1542. doi:10.3390/v13081542 34452407 PMC8402895

[3] Postel A, Nishi T, Kameyama K-I, Meyer D, Suckstorff O, Fukai K, Becher P. 2019. Reemergence of classical swine fever, Japan, 2018. Emerg Infect Dis 25:1228–1231. doi:10.3201/eid2506.181578 30870139 PMC6537743

[4] Becher P, Orlich M, Thiel HJ. 1998. Complete genomic sequence of border disease virus, a pestivirus from sheep. J Virol 72:5165–5173. doi:10.1128/JVI.72.6.5165-5173.1998 9573288 PMC110089

[5] Vantsis JT, Barlow RM, Gardiner AC, Linklater KA. 1980. The effects of challenge with homologous and heterologous strains of Border disease virus on ewes with previous experience of the disease. J Comp Pathol 90:39–45. doi:10.1016/0021-9975(80)90026-2 6248575

[6] Meyer D, Postel A, Wiedemann A, Cagatay GN, Ciulli S, Guercio A, Becher P. 2021. Comparative analysis of Tunisian sheep-like virus, Bungowannah virus and border disease virus infection in the porcine host. Viruses 13:1539. doi:10.3390/v13081539 34452404 PMC8402848

[7] Vilcek S, Belák S. 1996. Genetic identification of pestivirus strain Frijters as a border disease virus from pigs. J Virol Methods 60:103–108. doi:10.1016/0166-0934(96)02031-9 8795011

[8] Becher P, Orlich M, Shannon AD, Horner G, König M, Thiel HJ. 1997. Phylogenetic analysis of pestiviruses from domestic and wild ruminants. J Gen Virol 78:1357–1366. doi:10.1099/0022-1317-78-6-1357 9191930

[9] Roehe PM, Woodward MJ, Edwards S. 1992. Characterisation of p20 gene sequences from a border disease-like pestivirus isolated from pigs. Vet Microbiol 33:231–238. doi:10.1016/0378-1135(92)90051-t 1336241

[10] Huang Y-L, Meyer D, Postel A, Tsai K-J, Liu H-M, Yang C-H, Huang Y-C, Berkley N, Deng M-C, Wang F-I, Becher P, Crooke H, Chang C-Y. 2021. Identification of a common conformational epitope on the glycoprotein E2 of classical swine fever virus and border disease virus. Viruses 13:1655. doi:10.3390/v13081655 34452520 PMC8402670

[11] Collett MS, Larson R, Gold C, Strick D, Anderson DK, Purchio AF. 1988. Molecular cloning and nucleotide sequence of the pestivirus bovine viral diarrhea virus. Virology (Auckl) 165:191–199. doi:10.1016/0042-6822(88)90672-1 2838957

[12] Meyers G, Rümenapf T, Thiel HJ. 1989. Molecular cloning and nucleotide sequence of the genome of hog cholera virus. Virology (Auckl) 171:555–567. doi:10.1016/0042-6822(89)90625-9 2763466

[13] Moormann RJM, Warmerdam PAM, van der Meer B, Schaaper WMM, Wensvoort G, Hulst MM. 1990. Molecular cloning and nucleotide sequence of hog cholera virus strain brescia and mapping of the genomic region encoding envelope protein E1. Virology (Auckl) 177:184–198. doi:10.1016/0042-6822(90)90472-4 2162104

[14] Rümenapf T, Unger G, Strauss JH, Thiel HJ. 1993. Processing of the envelope glycoproteins of pestiviruses. J Virol 67:3288–3294. doi:10.1128/JVI.67.6.3288-3294.1993 8388499 PMC237670

[15] Heimann M, Roman-Sosa G, Martoglio B, Thiel H-J, Rümenapf T. 2006. Core protein of pestiviruses is processed at the C terminus by signal peptide peptidase. J Virol 80:1915–1921. doi:10.1128/JVI.80.4.1915-1921.2006 16439547 PMC1367156

[16] Thiel HJ, Stark R, Weiland E, Rümenapf T, Meyers G. 1991. Hog cholera virus: molecular composition of virions from a pestivirus. J Virol 65:4705–4712. doi:10.1128/JVI.65.9.4705-4712.1991 1870198 PMC248926

[17] Tautz N, Elbers K, Stoll D, Meyers G, Thiel HJ. 1997. Serine protease of pestiviruses: determination of cleavage sites. J Virol 71:5415–5422. doi:10.1128/JVI.71.7.5415-5422.1997 9188613 PMC191781

[18] Harada T, Tautz N, Thiel HJ. 2000. E2-p7 region of the bovine viral diarrhea virus polyprotein: processing and functional studies. J Virol 74:9498–9506. doi:10.1128/jvi.74.20.9498-9506.2000 11000219 PMC112379

[19] Lamp B, Riedel C, Roman-Sosa G, Heimann M, Jacobi S, Becher P, Thiel H-J, Rümenapf T. 2011. Biosynthesis of classical swine fever virus nonstructural proteins. J Virol 85:3607–3620. doi:10.1128/JVI.02206-10 21270154 PMC3067844

[20] Tautz N, Tews BA, Meyers G. 2015. The molecular biology of pestiviruses. Adv Virus Res 93:47–160. doi:10.1016/bs.aivir.2015.03.002 26111586

[21] Hulst MM, Westra DF, Wensvoort G, Moormann RJ. 1993. Glycoprotein E1 of hog cholera virus expressed in insect cells protects swine from hog cholera. J Virol 67:5435–5442. doi:10.1128/JVI.67.9.5435-5442.1993 8350404 PMC237945

[22] König M, Lengsfeld T, Pauly T, Stark R, Thiel HJ. 1995. Classical swine fever virus: independent induction of protective immunity by two structural glycoproteins. J Virol 69:6479–6486. doi:10.1128/JVI.69.10.6479-6486.1995 7666549 PMC189549

[23] Wang Z, Nie Y, Wang P, Ding M, Deng H. 2004. Characterization of classical swine fever virus entry by using pseudotyped viruses: E1 and E2 are sufficient to mediate viral entry. Virology (Auckl) 330:332–341. doi:10.1016/j.virol.2004.09.023 15527858

[24] Ronecker S, Zimmer G, Herrler G, Greiser-Wilke I, Grummer B. 2008. Formation of bovine viral diarrhea virus E1-E2 heterodimers is essential for virus entry and depends on charged residues in the transmembrane domains. J Gen Virol 89:2114–2121. doi:10.1099/vir.0.2008/001792-0 18753220

[25] Risatti GR, Borca MV, Kutish GF, Lu Z, Holinka LG, French RA, Tulman ER, Rock DL. 2005. The E2 glycoprotein of classical swine fever virus is a virulence determinant in swine. J Virol 79:3787–3796. doi:10.1128/JVI.79.6.3787-3796.2005 15731272 PMC1075681

[26] Risatti GR, Holinka LG, Carrillo C, Kutish GF, Lu Z, Tulman ER, Sainz IF, Borca MV. 2006. Identification of a novel virulence determinant within the E2 structural glycoprotein of classical swine fever virus. Virology (Auckl) 355:94–101. doi:10.1016/j.virol.2006.07.005 16908042

[27] Risatti GR, Holinka LG, Fernandez Sainz I, Carrillo C, Lu Z, Borca MV. 2007. N-linked glycosylation status of classical swine fever virus strain Brescia E2 glycoprotein influences virulence in swine. J Virol 81:924–933. doi:10.1128/JVI.01824-06 17108025 PMC1797485

[28] Yuan F, Li D, Li C, Zhang Y, Song H, Li S, Deng H, Gao GF, Zheng A. 2021. ADAM17 is an essential attachment factor for classical swine fever virus. PLoS Pathog 17:e1009393. doi:10.1371/journal.ppat.1009393 33684175 PMC7971878

[29] Vuono EA, Ramirez-Medina E, Azzinaro P, Berggren KA, Rai A, Pruitt S, Silva E, Velazquez-Salinas L, Borca MV, Gladue DP. 2020. SERTA domain containing protein 1 (SERTAD1) interacts with classical swine fever virus structural glycoprotein E2, which is involved in virus virulence in swine. Viruses 12:421. doi:10.3390/v12040421 32283651 PMC7232485

[30] Vuono EA, Ramirez-Medina E, Velazquez-Salinas L, Berggren K, Rai A, Pruitt S, Espinoza N, Gladue DP, Borca MV. 2021. Structural glycoprotein E2 of classical swine fever virus critically interacts with host protein torsin-1A during the virus infectious cycle. J Virol 95:e00314-21. doi:10.1128/JVI.00314-21 33827941 PMC8316129

[31] Vuono E, Ramirez-Medina E, Silva E, Berggren K, Rai A, Espinoza N, Gladue DP, Borca MV. 2023. Classical swine fever virus structural glycoprotein E2 interacts with host protein ACADM during the virus infectious cycle. Viruses 15:1036. doi:10.3390/v15051036 37243123 PMC10223252

[32] van Rijn PA, van Gennip HG, de Meijer EJ, Moormann RJ. 1993. Epitope mapping of envelope glycoprotein E1 of hog cholera virus strain Brescia. J Gen Virol 74:2053–2060. doi:10.1099/0022-1317-74-10-2053 7691986

[33] Li Y, Wang J, Kanai R, Modis Y. 2013. Crystal structure of glycoprotein E2 from bovine viral diarrhea virus. Proc Natl Acad Sci U S A 110:6805–6810. doi:10.1073/pnas.1300524110 23569276 PMC3637714

[34] El Omari K, Iourin O, Harlos K, Grimes JM, Stuart DI. 2013. Structure of a pestivirus envelope glycoprotein E2 clarifies its role in cell entry. Cell Rep 3:30–35. doi:10.1016/j.celrep.2012.12.001 23273918 PMC3607223

[35] van Rijn PA, Miedema GK, Wensvoort G, van Gennip HG, Moormann RJ. 1994. Antigenic structure of envelope glycoprotein E1 of hog cholera virus. J Virol 68:3934–3942. doi:10.1128/JVI.68.6.3934-3942.1994 7514680 PMC236899

[36] Wensvoort G, Terpstra C, Boonstra J, Bloemraad M, Van Zaane D. 1986. Production of monoclonal antibodies against swine fever virus and their use in laboratory diagnosis. Vet Microbiol 12:101–108. doi:10.1016/0378-1135(86)90072-6 2428160

[37] Peters W, Greiser-Wilke I, Moennig V, Liess B. 1986. Preliminary serological characterization of bovine viral diarrhoea virus strains using monoclonal antibodies. Vet Microbiol 12:195–200. doi:10.1016/0378-1135(86)90048-9 3022462

[38] Bolin S, Moennig V, Kelso Gourley NE, Ridpath J. 1988. Monoclonal antibodies with neutralizing activity segregate isolates of bovine viral diarrhea virus into groups. Brief report. Arch Virol 99:117–123. doi:10.1007/BF01311029 2833200

[39] Wensvoort G, Bloemraad M, Terpstra C. 1988. An enzyme immunoassay employing monoclonal antibodies and detecting specifically antibodies to classical swine fever virus. Vet Microbiol 17:129–140. doi:10.1016/0378-1135(88)90004-1 2459837

[40] Edwards S, Moennig V, Wensvoort G. 1991. The development of an international reference panel of monoclonal antibodies for the differentiation of hog cholera virus from other pestiviruses. Vet Microbiol 29:101–108. doi:10.1016/0378-1135(91)90118-y 1660637

[41] Donis RO, Corapi W, Dubovi EJ. 1988. Neutralizing monoclonal antibodies to bovine viral diarrhoea virus bind to the 56K to 58K glycoprotein. J Gen Virol 69:77–86. doi:10.1099/0022-1317-69-1-77 2447228

[42] Weiland E, Stark R, Haas B, Rümenapf T, Meyers G, Thiel HJ. 1990. Pestivirus glycoprotein which induces neutralizing antibodies forms part of a disulfide-linked heterodimer. J Virol 64:3563–3569. doi:10.1128/JVI.64.8.3563-3569.1990 2370675 PMC249648

[43] van Rijn PA. 2007. A common neutralizing epitope on envelope glycoprotein E2 of different pestiviruses: implications for improvement of vaccines and diagnostics for classical swine fever (CSF)? Vet Microbiol 125:150–156. doi:10.1016/j.vetmic.2007.05.001 17561359

[44] Xu Q, Guo J, Ma F, Liu L, Wang Y, Zhang S, Niu X, Li X, Jiang M, Wang Y, Wang L, Liu Y, Li Q, Chai S, Wang R, Ma Q, Zhang E, Zhang G. 2021. A novel linear epitope at the C-terminal region of the classical swine fever virus E2 protein elicits neutralizing activity. Int J Biol Macromol 189:837–846. doi:10.1016/j.ijbiomac.2021.08.088 34403672

[45] Ma Z, Zhao Y, Lv J, Pan L. 2023. Development and application of classical swine fever virus monoclonal antibodies derived from single B cells. Vet Res 54:90. doi:10.1186/s13567-023-01229-y 37845739 PMC10580647

[46] Kaufmann B, Vogt MR, Goudsmit J, Holdaway HA, Aksyuk AA, Chipman PR, Kuhn RJ, Diamond MS, Rossmann MG. 2010. Neutralization of West Nile virus by cross-linking of its surface proteins with Fab fragments of the human monoclonal antibody CR4354. Proc Natl Acad Sci U S A 107:18950–18955. doi:10.1073/pnas.1011036107 20956322 PMC2973864

[47] Mittler E, Wec AZ, Tynell J, Guardado-Calvo P, Wigren-Byström J, Polanco LC, O’Brien CM, Slough MM, Abelson DM, Serris A, et al.. 2022. Human antibody recognizing a quaternary epitope in the Puumala virus glycoprotein provides broad protection against orthohantaviruses. Sci Transl Med 14:eabl5399. doi:10.1126/scitranslmed.abl5399 35294259 PMC9805701

[48] Greiser‐Wilke I, Moennig V, Thon D, Rauter K. 1985. Characterization of monoclonal antibodies against Brucella melitensis. Zentralblatt für Veterinärmedizin Reihe B 32:616–627. doi:10.1111/j.1439-0450.1985.tb02002.x 3907209

[49] Greiser-Wilke I, Moennig V, Coulibaly CO, Dahle J, Leder L, Liess B. 1990. Identification of conserved epitopes on a hog cholera virus protein. Arch Virol 111:213–225. doi:10.1007/BF01311055 1693844

[50] Koerke J. 1989. Herstellung und Charakterisierung von monoklonalen Antikörpern gegen den Stamm A 1138/69 ds Virus der Bovinen Virusdiarrhoe. Hannover Tieraerztliche Hochschule Hannover

[51] EU and WOAH. Reference laboratory for classical swine fever. Available from: https://www.tiho-hannover.de/kliniken-institute/institute/institut-fuer-virologie/eu-and-woah-reference-laboratory. Retrieved 05 Apr 2024.

[52] Institut für Virologie. Available from: https://www.tiho-hannover.de/kliniken-institute/institute/institut-fuer-virologie. Retrieved 05 Apr 2024.

[53] Huang Y-L, Meyer D, Postel A, Tsai K-J, Liu H-M, Yang C-H, Huang Y-C, Chang H-W, Deng M-C, Wang F-I, Becher P, Crooke H, Chang C-Y. 2023. Identification of neutralizing epitopes on the D/A domain of the E2 glycoprotein of classical swine fever virus. Virus Res 336:199209. doi:10.1016/j.virusres.2023.199209 37633596 PMC10485151

[54] Roman-Sosa G, Leske A, Ficht X, Dau TH, Holzerland J, Hoenen T, Beer M, Kammerer R, Schirmbeck R, Rey FA, Cordo SM, Groseth A. 2022. Immunization with GP1 but not core-like particles displaying isolated receptor-binding epitopes elicits virus-neutralizing antibodies against Junín virus. Vaccines (Basel) 10:173. doi:10.3390/vaccines10020173 35214632 PMC8874384

[55] Aricescu AR, Lu W, Jones EY. 2006. A time- and cost-efficient system for high-level protein production in mammalian cells. Acta Crystallogr D Biol Crystallogr 62:1243–1250. doi:10.1107/S0907444906029799 17001101

[56] Lerch SB. 2006. Epitope mapping of the classical swine fever virus glycoprotein E2. Hannover University of Veterinary Medicine Hannover, Foundation

[57] Roman-Sosa G, Brocchi E, Schirrmeier H, Wernike K, Schelp C, Beer M. 2016. Analysis of the humoral immune response against the envelope glycoprotein Gc of Schmallenberg virus reveals a domain located at the amino terminus targeted by mAbs with neutralizing activity. J Gen Virol 97:571–580. doi:10.1099/jgv.0.000377 26684324

[58] Mirdita M, Schütze K, Moriwaki Y, Heo L, Ovchinnikov S, Steinegger M. 2022. ColabFold: making protein folding accessible to all. Nat Methods 19:679–682. doi:10.1038/s41592-022-01488-1 35637307 PMC9184281

[59] Wang Q, Groenendyk J, Michalak M. 2015. Glycoprotein quality control and endoplasmic reticulum stress. Molecules 20:13689–13704. doi:10.3390/molecules200813689 26225950 PMC6331979

[60] Bold D, Roman-Sosa G, Gaudreault NN, Zayat B, Pogranichniy RM, Richt JA. 2022. Development of an indirect ELISA for the detection of SARS-CoV-2 antibodies in cats. Front Vet Sci 9:864884. doi:10.3389/fvets.2022.864884 35754530 PMC9226769

[61] Iourin O, Harlos K, El Omari K, Lu W, Kadlec J, Iqbal M, Meier C, Palmer A, Jones I, Thomas C, Brownlie J, Grimes JM, Stuart DI. 2013. Expression, purification and crystallization of the ectodomain of the envelope glycoprotein E2 from Bovine viral diarrhoea virus. Acta Crystallogr Sect F Struct Biol Cryst Commun 69:35–38. doi:10.1107/S1744309112049184 PMC3539699 23295482

[62] Pannhorst K, Fröhlich A, Staubach C, Meyer D, Blome S, Becher P. 2015. Evaluation of an E^rns^-based enzyme-linked immunosorbent assay to distinguish Classical swine fever virus–infected pigs from pigs vaccinated with CP7_E2alf. J Vet Diagn Invest 27:449–460. doi:10.1177/1040638715592446 26179095

[63] Zhou Y, Moennig V, Coulibaly CO, Dahle J, Liess B. 1989. Differentiation of hog cholera and bovine virus diarrhoea viruses in pigs using monoclonal antibodies. Zentralbl Veterinarmed B 36:76–80. doi:10.1111/j.1439-0450.1989.tb00573.x 2538980

[64] Wang L, Mi S, Madera R, Ganges L, Borca MV, Ren J, Cunningham C, Cino-Ozuna AG, Li H, Tu C, Gong W, Shi J. 2020. A neutralizing monoclonal antibody-based competitive ELISA for classical swine fever C-strain post-vaccination monitoring. BMC Vet Res 16:14. doi:10.1186/s12917-020-2237-6 31937302 PMC6958719

[65] Dong H, Su A, Lv D, Ma L, Dong J, Guo N, Ren L, Jiao H, Pang D, Ouyan H. 2019. Development of whole-porcine monoclonal antibodies with potent neutralization activity against classical swine fever virus from single B cells. ACS Synth Biol 8:989–1000. doi:10.1021/acssynbio.8b00365 30935202

[66] Wang L, Madera R, Li Y, Gladue DP, Borca MV, McIntosh MT, Shi J. 2023. Development of porcine monoclonal antibodies with in vitro neutralizing activity against classical swine fever virus from C-strain E2-specific single B cells. Viruses 15:863. doi:10.3390/v15040863 37112845 PMC10145741

[67] Xu H, Han G, Lu Y, Liu Z, Tao L, He F. 2021. Broad neutralization of CSFV with novel monoclonal antibodies in vivo. Int J Biol Macromol 173:513–523. doi:10.1016/j.ijbiomac.2021.01.142 33493566

[68] Chang C-Y, Huang C-C, Deng M-C, Huang Y-L, Lin Y-J, Liu H-M, Lin Y-L, Wang F-I. 2012. Identification of conformational epitopes and antigen-specific residues at the D/A domains and the extramembrane C-terminal region of E2 glycoprotein of classical swine fever virus. Virus Res 168:56–63. doi:10.1016/j.virusres.2012.06.013 22727685

[69] Lin M, Lin F, Mallory M, Clavijo A. 2000. Deletions of structural glycoprotein E2 of classical swine fever virus strain alfort/187 resolve a linear epitope of monoclonal antibody WH303 and the minimal N-terminal domain essential for binding immunoglobulin G antibodies of a pig hyperimmune serum. J Virol 74:11619–11625. doi:10.1128/jvi.74.24.11619-11625.2000 11090160 PMC112443

[70] Tong C, Chen N, Liao X, Xie W, Li D, Li X, Fang W. 2015. The epitope recognized by monoclonal antibody 2B6 in the B/C domains of classical swine fever virus glycoprotein E2 affects viral binding to hyperimmune sera and replication. J Microbiol Biotechnol 25:537–546. doi:10.4014/jmb.1407.07073 25370727

[71] Mi S, Wang L, Li H, Bao F, Madera R, Shi X, Zhang L, Mao Y, Yan R, Xia X, Gong W, Shi J, Tu C. 2022. Characterization of monoclonal antibodies that specifically differentiate field isolates from vaccine strains of classical swine fever virus. Front Immunol 13:930631. doi:10.3389/fimmu.2022.930631 35958565 PMC9361847

[72] Peng W-P, Hou Q, Xia Z-H, Chen D, Li N, Sun Y, Qiu H-J. 2008. Identification of a conserved linear B-cell epitope at the N-terminus of the E2 glycoprotein of Classical swine fever virus by phage-displayed random peptide library. Virus Res 135:267–272. doi:10.1016/j.virusres.2008.04.003 18485511

[73] Zhang Y-N, Liu Y-Y, Xiao F-C, Liu C-C, Liang X-D, Chen J, Zhou J, Baloch AS, Kan L, Zhou B, Qiu H-J. 2018. Rab5, Rab7, and Rab11 are required for caveola-dependent endocytosis of classical swine fever virus in porcine alveolar macrophages. J Virol 92:e00797-18. doi:10.1128/JVI.00797-18 29769350 PMC6052321

[74] Krey T, Thiel H-J, Rümenapf T. 2005. Acid-resistant bovine pestivirus requires activation for pH-triggered fusion during entry. J Virol 79:4191–4200. doi:10.1128/JVI.79.7.4191-4200.2005 15767420 PMC1061521

[75] Battini L, Fidalgo DM, Álvarez DE, Bollini M. 2021. Discovery of a potent and selective chikungunya virus envelope protein inhibitor through computer-aided drug design. ACS Infect Dis 7:1503–1518. doi:10.1021/acsinfecdis.0c00915 34048233

[76] Meyer WJ, Johnston RE. 1993. Structural rearrangement of infecting Sindbis virions at the cell surface: mapping of newly accessible epitopes. J Virol 67:5117–5125. doi:10.1128/JVI.67.9.5117-5125.1993 7688818 PMC237909

