# Erratum for Roman-Sosa et al., “Virus-neutralizing monoclonal antibodies against bovine viral diarrhea virus and classical swine fever virus target conformational and linear epitopes on E2 glycoprotein subdomains”

**DOI:** 10.1128/spectrum.01869-25

**Published:** 2025-07-21

**Authors:** Gleyder Roman-Sosa, Denise Meyer, Mariano Dellarole, Doris à Wengen, Susanne Lerch, Alexander Postel, Paul Becher

## ERRATUM

Volume 13, no. 4, e02041-24, 2025, https://doi.org/10.1128/spectrum.02041-24. Page 3, Table 1: The IgG isotype assigned to mAb BVD/CA3 as “IgG2b” should read “IgG1.”

