# Supplementary material for: Virus-neutralizing monoclonal antibodies against bovine viral diarrhea virus and classical swine fever virus target conformational and linear epitopes on E2 glycoprotein subdomains
Source: Microbiol Spectr. 2025 Feb 25;13(4):e02041-24. doi: 10.1128/spectrum.02041-24 (PMC11960116; doi:10.1128/spectrum.02041-24)
Supplement: Supplemental material — Nucleotide sequences of plasmids. [file spectrum.02041-24-s0001.docx]

**Supplementary material and methods.**

Sequence of plasmids.

The sequence of the sites for the restriction enzymes are highlighted in bold letters with a grey background.

**pCSFV E2.**

gtcgacattgattattgactagttattaatagtaatcaattacggggtcattagttcatagcccatatatggagttccgcgttacataacttacggtaaatggcccgcctggctgaccgcccaacgacccccgcccattgacgtcaataatgacgtatgttcccatagtaacgccaatagggactttccattgacgtcaatgggtggagtatttacggtaaactgcccacttggcagtacatcaagtgtatcatatgccaagtacgccccctattgacgtcaatgacggtaaatggcccgcctggcattatgcccagtacatgaccttatgggactttcctacttggcagtacatctacgtattagtcatcgctattaccatggtcgaggtgagccccacgttctgcttcactctccccatctcccccccctccccacccccaattttgtatttatttattttttaattattttgtgcagcgatgggggcggggggggggggggggcgcgcgccaggcggggcggggcggggcgaggggcggggcggggcgaggcggagaggtgcggcggcagccaatcagagcggcgcgctccgaaagtttccttttatggcgaggcggcggcggcggcggccctataaaaagcgaagcgcgcggcgggcgggagtcgctgcgcgctgccttcgccccgtgccccgctccgccgccgcctcgcgccgcccgccccggctctgactgaccgcgttactcccacaggtgagcgggcgggacggcccttctcctccgggctgtaattagcgcttggtttaatgacggcttgtttcttttctgtggctgcgtgaaagccttgaggggctccgggagggccctttgtgcggggggagcggctcggggggtgcgtgcgtgtgtgtgtgcgtggggagcgccgcgtgcggctccgcgctgcccggcggctgtgagcgctgcgggcgcggcgcggggctttgtgcgctccgcagtgtgcgcgaggggagcgcggccgggggcggtgccccgcggtgcggggggggctgcgaggggaacaaaggctgcgtgcggggtgtgtgcgtgggggggtgagcagggggtgtgggcgcgtcggtcgggctgcaaccccccctgcacccccctccccgagttgctgagcacggcccggcttcgggtgcggggctccgtacggggcgtggcgcggggctcgccgtgccgggcggggggtggcggcaggtgggggtgccgggcggggcggggccgcctcgggccggggagggctcgggggaggggcgcggcggcccccggagcgccggcggctgtcgaggcgcggcgagccgcagccattgccttttatggtaatcgtgcgagagggcgcagggacttcctttgtcccaaatctgtgcggagccgaaatctgggaggcgccgccgcaccccctctagcgggcgcggggcgaagcggtgcggcgccggcaggaaggaaatgggcggggagggccttcgtgcgtcgccgcgccgccgtccccttctccctctccagcctcggggctgtccgcggggggacggctgccttcgggggggacggggcagggcggggttcggcttctggcgtgtgaccggcggctctagagcctctgctaaccatgttcatgccttcttctttttcctacagctcctgggcaacgtgctggttattgtgctgtctcatcattttggcaaagaattgcggccgtctcaggccgaattcaagcttgccaccatggggatccttcccagccctgggatgcctgcgctgctctccctcgtgagccttctctccgtgctgctgatgggttgcgtagctgaa**ACCGGT**cagctagcctgcaaggaagattacaggtacgcaatatcatcaaccaatgagatagggctactcggggccggaggtctcaccaccacctggaaagaatacaaccacgatttgcaactgaatgacgggaccgttaaggccatttgcgtggcaggttcctttaaagtcacagcacttaatgtggtcagtaggaggtatttggcatcattgcataaggaggctttacccacttccgtgacattcgagctcctgttcgacgggaccaacccatcaactgaggaaatgggagatgacttcgggttcgggctgtgcccgtttgatacgagtcctgttgtcaagggaaagtacaatacaaccttgttgaacggtagtgctttctatcttgtctgtccaatagggtggacgggtgttatagagtgcacagcagtgagcccaacaactctgagaacagaagtggtaaagaccttcaggagggacaagccctttccgcacagaatggattgtgtgaccacaacagtggaaaatgaagatttattctactgtaagttggggggcaactggacatgtgtgaaaggtgaaccagtggtctacacgggggggctagtaaaacaatgcagatggtgtggctttgacttcaatgagcctgacggactcccacactaccccataggtaagtgcattttggcaaatgagacaggttacagaatagtggattcaacagactgtaacagagacggtgttgtaatcagcacagaggggagtcatgagtgcttgatcggtaacacaactgtcaaggtgcatgcatcagatgaaagactgggccccatgccatgcagacctaaagagatcgtctctagtgcaggacctgtaaggaaaacttcctgtacattcaactacgcaaaaactttgaagaacaagtactatgagcccagggacagctacttccagcaatatatgcttaagggcgagtatcagtactggtttgacctggacgtgactgaccgccactca**GGTACC**ggctcctccggactgaatgacatctttgaggcccagaagatcgagtggcacgagtccggcggcggatctcttgtaccgcgaggtagttggtcccaccctcagttcgagaagggcggaggctccggcggcggatctggaggaggctcctggtcccacccccagttcgagaaatgataactcgagactagtatcgcgataattcactcctcaggtgcaggctgcctatcagaaggtggtggctggtgtggccaatgccctggctcacaaataccactgagatctttttccctctgccaaaaattatggggacatcatgaagccccttgagcatctgacttctggctaataaaggaaatttattttcattgcaatagtgtgttggaattttttgtgtctctcactcggaaggacatatgggagggcaaatcatttaaaacatcagaatgagtatttggtttagagtttggcaacatatgcccatatgctggctgccatgaacaaaggttggctataaagaggtcatcagtatatgaaacagccccctgctgtccattccttattccatagaaaagccttgacttgaggttagattttttttatattttgttttgtgttatttttttctttaacatccctaaaattttccttacatgttttactagccagatttttcctcctctcctgactactcccagtcatagctgtccctcttctcttatggagatccctcgacctgcagcccaagcttggcgtaatcatggtcatagctgtttcctgtgtgaaattgttatccgctcacaattccacacaacatacgagccggaagcataaagtgtaaagcctggggtgcctaatgagtgagctaactcacattaattgcgttgcgctcactgcccgctttccagtcgggaaacctgtcgtgccagcggatcgatccgctgcattaatgaatcggccaacgcgcggggagaggcggtttgcgtattgggcgctcttccgcttcctcgctcactgactcgctgcgctcggtcgttcggctgcggcgagcggtatcagctcactcaaaggcggtaatacggttatccacagaatcaggggataacgcaggaaagaacatgtgagcaaaaggccagcaaaaggccaggaaccgtaaaaaggccgcgttgctggcgtttttccataggctccgcccccctgacgagcatcacaaaaatcgacgctcaagtcagaggtggcgaaacccgacaggactataaagataccaggcgtttccccctggaagctccctcgtgcgctctcctgttccgaccctgccgcttaccggatacctgtccgcctttctcccttcgggaagcgtggcgctttctcatagctcacgctgtaggtatctcagttcggtgtaggtcgttcgctccaagctgggctgtgtgcacgaaccccccgttcagcccgaccgctgcgccttatccggtaactatcgtcttgagtccaacccggtaagacacgacttatcgccactggcagcagccactggtaacaggattagcagagcgaggtatgtaggcggtgctacagagttcttgaagtggtggcctaactacggctacactagaagaacagtatttggtatctgcgctctgctgaagccagttaccttcggaaaaagagttggtagctcttgatccggcaaacaaaccaccgctggtagcggtggtttttttgtttgcaagcagcagattacgcgcagaaaaaaaggatctcaagaagatcctttgatcttttctacggggtctgacgctcagtggaacgaaaactcacgttaagggattttggtcatgagattatcaaaaaggatcttcacctagatccttttaaattaaaaatgaagttttaaatcaatctaaagtatatatgagtaaacttggtctgacagttaccaatgcttaatcagtgaggcacctatctcagcgatctgtctatttcgttcatccatagttgcctgactccccgtcgtgtagataactacgatacgggagggcttaccatctggccccagtgctgcaatgataccgcgagacccacgctcaccggctccagatttatcagcaataaaccagccagccggaagggccgagcgcagaagtggtcctgcaactttatccgcctccatccagtctattaattgttgccgggaagctagagtaagtagttcgccagttaatagtttgcgcaacgttgttgccattgctacaggcatcgtggtgtcacgctcgtcgtttggtatggcttcattcagctccggttcccaacgatcaaggcgagttacatgatcccccatgttgtgcaaaaaagcggttagctccttcggtcctccgatcgttgtcagaagtaagttggccgcagtgttatcactcatggttatggcagcactgcataattctcttactgtcatgccatccgtaagatgcttttctgtgactggtgagtactcaaccaagtcattctgagaatagtgtatgcggcgaccgagttgctcttgcccggcgtcaatacgggataataccgcgccacatagcagaactttaaaagtgctcatcattggaaaacgttcttcggggcgaaaactctcaaggatcttaccgctgttgagatccagttcgatgtaacccactcgtgcacccaactgatcttcagcatcttttactttcaccagcgtttctgggtgagcaaaaacaggaaggcaaaatgccgcaaaaaagggaataagggcgacacggaaatgttgaatactcatactcttcctttttcaatattattgaagcatttatcagggttattgtctcatgagcggatacatatttgaatgtatttagaaaaataaacaaataggggttccgcgcacatttccccgaaaagtgccacctgg

**pBVDV E2.**

gtcgacattgattattgactagttattaatagtaatcaattacggggtcattagttcatagcccatatatggagttccgcgttacataacttacggtaaatggcccgcctggctgaccgcccaacgacccccgcccattgacgtcaataatgacgtatgttcccatagtaacgccaatagggactttccattgacgtcaatgggtggagtatttacggtaaactgcccacttggcagtacatcaagtgtatcatatgccaagtacgccccctattgacgtcaatgacggtaaatggcccgcctggcattatgcccagtacatgaccttatgggactttcctacttggcagtacatctacgtattagtcatcgctattaccatggtcgaggtgagccccacgttctgcttcactctccccatctcccccccctccccacccccaattttgtatttatttattttttaattattttgtgcagcgatgggggcggggggggggggggggcgcgcgccaggcggggcggggcggggcgaggggcggggcggggcgaggcggagaggtgcggcggcagccaatcagagcggcgcgctccgaaagtttccttttatggcgaggcggcggcggcggcggccctataaaaagcgaagcgcgcggcgggcgggagtcgctgcgcgctgccttcgccccgtgccccgctccgccgccgcctcgcgccgcccgccccggctctgactgaccgcgttactcccacaggtgagcgggcgggacggcccttctcctccgggctgtaattagcgcttggtttaatgacggcttgtttcttttctgtggctgcgtgaaagccttgaggggctccgggagggccctttgtgcggggggagcggctcggggggtgcgtgcgtgtgtgtgtgcgtggggagcgccgcgtgcggctccgcgctgcccggcggctgtgagcgctgcgggcgcggcgcggggctttgtgcgctccgcagtgtgcgcgaggggagcgcggccgggggcggtgccccgcggtgcggggggggctgcgaggggaacaaaggctgcgtgcggggtgtgtgcgtgggggggtgagcagggggtgtgggcgcgtcggtcgggctgcaaccccccctgcacccccctccccgagttgctgagcacggcccggcttcgggtgcggggctccgtacggggcgtggcgcggggctcgccgtgccgggcggggggtggcggcaggtgggggtgccgggcggggcggggccgcctcgggccggggagggctcgggggaggggcgcggcggcccccggagcgccggcggctgtcgaggcgcggcgagccgcagccattgccttttatggtaatcgtgcgagagggcgcagggacttcctttgtcccaaatctgtgcggagccgaaatctgggaggcgccgccgcaccccctctagcgggcgcggggcgaagcggtgcggcgccggcaggaaggaaatgggcggggagggccttcgtgcgtcgccgcgccgccgtccccttctccctctccagcctcggggctgtccgcggggggacggctgccttcgggggggacggggcagggcggggttcggcttctggcgtgtgaccggcggctctagagcctctgctaaccatgttcatgccttcttctttttcctacagctcctgggcaacgtgctggttattgtgctgtctcatcattttggcaaagaattgcggccgtctcaggccgaattcaagcttgccaccatggggatccttcccagccctgggatgcctgcgctgctctccctcgtgagccttctctccgtgctgctgatgggttgcgtagctgaa**ACCGGT**cacttggattgcaaacctgaattctcgtatgccatagcaaaggacgaaagaattggtcaactgggggctgaaggccttaccaccacttggaaggaatactcacctggaatgaagctggaagacacaatggtcattgcttggtgcgaagatgggaagttaatgtacctccaaagatgcacgagagaaaccagatatctcgcaatcttgcatacaagagccttgccgaccagtgtggtattcaaaaaactctttgatgggcgaaagcaagaggatgtagtcgaaatgaacgacaactttgaatttggactctgcccatgtgatgccaaacccatagtaagagggaagttcaatacaacgctgctgaacggaccggccttccagatggtatgccccataggatggacagggactgtaagctgtacgtcattcaatatggacaccttagccacaactgtggtacggacatatagaaggtctaaaccattccctcataggcaaggctgtatcacccaaaagaatctgggggaggatctccataactgcatccttggaggaaattggacttgtgtgcctggagaccaactactatacaaagggggctctattgaatcttgcaagtggtgtggctatcaatttaaagagagtgagggactaccacactaccccattggcaagtgtaaattggagaacgagactggttacaggctagtagacagtacctcttgcaatagagaaggtgtggccatagtaccacaagggacattaaagtgcaagataggaaaaacaactgtacaggtcatagctatggataccaaactcggGcctatgccttgcagaccatatgaaatcatatcaagtgaggggcctgtagaaaagacagcgtgtactttcaactacactaagacattaaaaaataagtattttgagcccagagacagctactttcagcaatacatgctaaaaggagagtatcaatactggtttgacctggaggtgactgaccatcaccgg**GGTACC**ggctcctccggactgaatgacatctttgaggcccagaagatcgagtggcacgagtccggcggcggatctcttgtaccgcgaggtagttggtcccaccctcagttcgagaagggcggaggctccggcggcggatctggaggaggctcctggtcccacccccagttcgagaaatgataactcgagactagtatcgcgataattcactcctcaggtgcaggctgcctatcagaaggtggtggctggtgtggccaatgccctggctcacaaataccactgagatctttttccctctgccaaaaattatggggacatcatgaagccccttgagcatctgacttctggctaataaaggaaatttattttcattgcaatagtgtgttggaattttttgtgtctctcactcggaaggacatatgggagggcaaatcatttaaaacatcagaatgagtatttggtttagagtttggcaacatatgcccatatgctggctgccatgaacaaaggttggctataaagaggtcatcagtatatgaaacagccccctgctgtccattccttattccatagaaaagccttgacttgaggttagattttttttatattttgttttgtgttatttttttctttaacatccctaaaattttccttacatgttttactagccagatttttcctcctctcctgactactcccagtcatagctgtccctcttctcttatggagatccctcgacctgcagcccaagcttggcgtaatcatggtcatagctgtttcctgtgtgaaattgttatccgctcacaattccacacaacatacgagccggaagcataaagtgtaaagcctggggtgcctaatgagtgagctaactcacattaattgcgttgcgctcactgcccgctttccagtcgggaaacctgtcgtgccagcggatcgatccgctgcattaatgaatcggccaacgcgcggggagaggcggtttgcgtattgggcgctcttccgcttcctcgctcactgactcgctgcgctcggtcgttcggctgcggcgagcggtatcagctcactcaaaggcggtaatacggttatccacagaatcaggggataacgcaggaaagaacatgtgagcaaaaggccagcaaaaggccaggaaccgtaaaaaggccgcgttgctggcgtttttccataggctccgcccccctgacgagcatcacaaaaatcgacgctcaagtcagaggtggcgaaacccgacaggactataaagataccaggcgtttccccctggaagctccctcgtgcgctctcctgttccgaccctgccgcttaccggatacctgtccgcctttctcccttcgggaagcgtggcgctttctcatagctcacgctgtaggtatctcagttcggtgtaggtcgttcgctccaagctgggctgtgtgcacgaaccccccgttcagcccgaccgctgcgccttatccggtaactatcgtcttgagtccaacccggtaagacacgacttatcgccactggcagcagccactggtaacaggattagcagagcgaggtatgtaggcggtgctacagagttcttgaagtggtggcctaactacggctacactagaagaacagtatttggtatctgcgctctgctgaagccagttaccttcggaaaaagagttggtagctcttgatccggcaaacaaaccaccgctggtagcggtggtttttttgtttgcaagcagcagattacgcgcagaaaaaaaggatctcaagaagatcctttgatcttttctacggggtctgacgctcagtggaacgaaaactcacgttaagggattttggtcatgagattatcaaaaaggatcttcacctagatccttttaaattaaaaatgaagttttaaatcaatctaaagtatatatgagtaaacttggtctgacagttaccaatgcttaatcagtgaggcacctatctcagcgatctgtctatttcgttcatccatagttgcctgactccccgtcgtgtagataactacgatacgggagggcttaccatctggccccagtgctgcaatgataccgcgagacccacgctcaccggctccagatttatcagcaataaaccagccagccggaagggccgagcgcagaagtggtcctgcaactttatccgcctccatccagtctattaattgttgccgggaagctagagtaagtagttcgccagttaatagtttgcgcaacgttgttgccattgctacaggcatcgtggtgtcacgctcgtcgtttggtatggcttcattcagctccggttcccaacgatcaaggcgagttacatgatcccccatgttgtgcaaaaaagcggttagctccttcggtcctccgatcgttgtcagaagtaagttggccgcagtgttatcactcatggttatggcagcactgcataattctcttactgtcatgccatccgtaagatgcttttctgtgactggtgagtactcaaccaagtcattctgagaatagtgtatgcggcgaccgagttgctcttgcccggcgtcaatacgggataataccgcgccacatagcagaactttaaaagtgctcatcattggaaaacgttcttcggggcgaaaactctcaaggatcttaccgctgttgagatccagttcgatgtaacccactcgtgcacccaactgatcttcagcatcttttactttcaccagcgtttctgggtgagcaaaaacaggaaggcaaaatgccgcaaaaaagggaataagggcgacacggaaatgttgaatactcatactcttcctttttcaatattattgaagcatttatcagggttattgtctcatgagcggatacatatttgaatgtatttagaaaaataaacaaataggggttccgcgcacatttccccgaaaagtgccacctgg

**pCSFV E2-*p*BVDV-B-C.**

gtcgacattgattattgactagttattaatagtaatcaattacggggtcattagttcatagcccatatatggagttccgcgttacataacttacggtaaatggcccgcctggctgaccgcccaacgacccccgcccattgacgtcaataatgacgtatgttcccatagtaacgccaatagggactttccattgacgtcaatgggtggagtatttacggtaaactgcccacttggcagtacatcaagtgtatcatatgccaagtacgccccctattgacgtcaatgacggtaaatggcccgcctggcattatgcccagtacatgaccttatgggactttcctacttggcagtacatctacgtattagtcatcgctattaccatggtcgaggtgagccccacgttctgcttcactctccccatctcccccccctccccacccccaattttgtatttatttattttttaattattttgtgcagcgatgggggcggggggggggggggggcgcgcgccaggcggggcggggcggggcgaggggcggggcggggcgaggcggagaggtgcggcggcagccaatcagagcggcgcgctccgaaagtttccttttatggcgaggcggcggcggcggcggccctataaaaagcgaagcgcgcggcgggcgggagtcgctgcgcgctgccttcgccccgtgccccgctccgccgccgcctcgcgccgcccgccccggctctgactgaccgcgttactcccacaggtgagcgggcgggacggcccttctcctccgggctgtaattagcgcttggtttaatgacggcttgtttcttttctgtggctgcgtgaaagccttgaggggctccgggagggccctttgtgcggggggagcggctcggggggtgcgtgcgtgtgtgtgtgcgtggggagcgccgcgtgcggctccgcgctgcccggcggctgtgagcgctgcgggcgcggcgcggggctttgtgcgctccgcagtgtgcgcgaggggagcgcggccgggggcggtgccccgcggtgcggggggggctgcgaggggaacaaaggctgcgtgcggggtgtgtgcgtgggggggtgagcagggggtgtgggcgcgtcggtcgggctgcaaccccccctgcacccccctccccgagttgctgagcacggcccggcttcgggtgcggggctccgtacggggcgtggcgcggggctcgccgtgccgggcggggggtggcggcaggtgggggtgccgggcggggcggggccgcctcgggccggggagggctcgggggaggggcgcggcggcccccggagcgccggcggctgtcgaggcgcggcgagccgcagccattgccttttatggtaatcgtgcgagagggcgcagggacttcctttgtcccaaatctgtgcggagccgaaatctgggaggcgccgccgcaccccctctagcgggcgcggggcgaagcggtgcggcgccggcaggaaggaaatgggcggggagggccttcgtgcgtcgccgcgccgccgtccccttctccctctccagcctcggggctgtccgcggggggacggctgccttcgggggggacggggcagggcggggttcggcttctggcgtgtgaccggcggctctagagcctctgctaaccatgttcatgccttcttctttttcctacagctcctgggcaacgtgctggttattgtgctgtctcatcattttggcaaagaattgcggccgtctcaggccgaattcaagcttgccaccatggggatccttcccagccctgggatgcctgcgctgctctccctcgtgagccttctctccgtgctgctgatgggttgcgtagctgaa**ACCGGT**cagctagcctgcaaggaagattacaggtacgcaatatcatcaaccaatgagatagggctactcggggccggaggtctcaccaccacctggaaagaatacaaccacgatttgcaactgaatgacgggaccgttaaggccatttgcgtggcaggttcctttaaagtcacagcacttaatgtggtcagtaggaggtatttggcatcattgcataaggaggctttacccacttccgtgacattcgagctcctgttcgacgggaccaacccatcaactgaggaaatgggagatgacttcgggttcgggctgtgcccgtttgatacgagtcctgttgtaagagggaagttcaatacaacgctgctgaacggaccggccttccagatggtatgccccataggatggacagggactgtaagctgtacgtcattcaatatggacaccttagccacaactgtggtacggacatatagaaggtctaaaccattccctcataggcaaggctgtatcacccaaaagaatctgggggaagatttattctactgtaagttggggggcaactggacatgtgtgaaaggtgaaccagtggtctacacgggggggctagtaaaacaatgcagatggtgtggctttgacttcaatgagcctgacggactcccacactaccccataggtaagtgcattttggcaaatgagacaggttacagaatagtggattcaacagactgtaacagagacggtgttgtaatcagcacagaggggagtcatgagtgcttgatcggtaacacaactgtcaaggtgcatgcatcagatgaaagactgggccccatgccatgcagacctaaagagatcgtctctagtgcaggacctgtaaggaaaacttcctgtacattcaactacgcaaaaactttgaagaacaagtactatgagcccagggacagctacttccagcaatatatgcttaagggcgagtatcagtactggtttgacctggacgtgactgaccgccactca**GGTACC**ggctcctccggactgaatgacatctttgaggcccagaagatcgagtggcacgagtccggcggcggatctcttgtaccgcgaggtagttggtcccaccctcagttcgagaagggcggaggctccggcggcggatctggaggaggctcctggtcccacccccagttcgagaaatgataactcgagactagtatcgcgataattcactcctcaggtgcaggctgcctatcagaaggtggtggctggtgtggccaatgccctggctcacaaataccactgagatctttttccctctgccaaaaattatggggacatcatgaagccccttgagcatctgacttctggctaataaaggaaatttattttcattgcaatagtgtgttggaattttttgtgtctctcactcggaaggacatatgggagggcaaatcatttaaaacatcagaatgagtatttggtttagagtttggcaacatatgcccatatgctggctgccatgaacaaaggttggctataaagaggtcatcagtatatgaaacagccccctgctgtccattccttattccatagaaaagccttgacttgaggttagattttttttatattttgttttgtgttatttttttctttaacatccctaaaattttccttacatgttttactagccagatttttcctcctctcctgactactcccagtcatagctgtccctcttctcttatggagatccctcgacctgcagcccaagcttggcgtaatcatggtcatagctgtttcctgtgtgaaattgttatccgctcacaattccacacaacatacgagccggaagcataaagtgtaaagcctggggtgcctaatgagtgagctaactcacattaattgcgttgcgctcactgcccgctttccagtcgggaaacctgtcgtgccagcggatcgatccgctgcattaatgaatcggccaacgcgcggggagaggcggtttgcgtattgggcgctcttccgcttcctcgctcactgactcgctgcgctcggtcgttcggctgcggcgagcggtatcagctcactcaaaggcggtaatacggttatccacagaatcaggggataacgcaggaaagaacatgtgagcaaaaggccagcaaaaggccaggaaccgtaaaaaggccgcgttgctggcgtttttccataggctccgcccccctgacgagcatcacaaaaatcgacgctcaagtcagaggtggcgaaacccgacaggactataaagataccaggcgtttccccctggaagctccctcgtgcgctctcctgttccgaccctgccgcttaccggatacctgtccgcctttctcccttcgggaagcgtggcgctttctcatagctcacgctgtaggtatctcagttcggtgtaggtcgttcgctccaagctgggctgtgtgcacgaaccccccgttcagcccgaccgctgcgccttatccggtaactatcgtcttgagtccaacccggtaagacacgacttatcgccactggcagcagccactggtaacaggattagcagagcgaggtatgtaggcggtgctacagagttcttgaagtggtggcctaactacggctacactagaagaacagtatttggtatctgcgctctgctgaagccagttaccttcggaaaaagagttggtagctcttgatccggcaaacaaaccaccgctggtagcggtggtttttttgtttgcaagcagcagattacgcgcagaaaaaaaggatctcaagaagatcctttgatcttttctacggggtctgacgctcagtggaacgaaaactcacgttaagggattttggtcatgagattatcaaaaaggatcttcacctagatccttttaaattaaaaatgaagttttaaatcaatctaaagtatatatgagtaaacttggtctgacagttaccaatgcttaatcagtgaggcacctatctcagcgatctgtctatttcgttcatccatagttgcctgactccccgtcgtgtagataactacgatacgggagggcttaccatctggccccagtgctgcaatgataccgcgagacccacgctcaccggctccagatttatcagcaataaaccagccagccggaagggccgagcgcagaagtggtcctgcaactttatccgcctccatccagtctattaattgttgccgggaagctagagtaagtagttcgccagttaatagtttgcgcaacgttgttgccattgctacaggcatcgtggtgtcacgctcgtcgtttggtatggcttcattcagctccggttcccaacgatcaaggcgagttacatgatcccccatgttgtgcaaaaaagcggttagctccttcggtcctccgatcgttgtcagaagtaagttggccgcagtgttatcactcatggttatggcagcactgcataattctcttactgtcatgccatccgtaagatgcttttctgtgactggtgagtactcaaccaagtcattctgagaatagtgtatgcggcgaccgagttgctcttgcccggcgtcaatacgggataataccgcgccacatagcagaactttaaaagtgctcatcattggaaaacgttcttcggggcgaaaactctcaaggatcttaccgctgttgagatccagttcgatgtaacccactcgtgcacccaactgatcttcagcatcttttactttcaccagcgtttctgggtgagcaaaaacaggaaggcaaaatgccgcaaaaaagggaataagggcgacacggaaatgttgaatactcatactcttcctttttcaatattattgaagcatttatcagggttattgtctcatgagcggatacatatttgaatgtatttagaaaaataaacaaataggggttccgcgcacatttccccgaaaagtgccacctgg

**pCSFV E2-*p*BVDV-B.**

gtcgacattgattattgactagttattaatagtaatcaattacggggtcattagttcatagcccatatatggagttccgcgttacataacttacggtaaatggcccgcctggctgaccgcccaacgacccccgcccattgacgtcaataatgacgtatgttcccatagtaacgccaatagggactttccattgacgtcaatgggtggagtatttacggtaaactgcccacttggcagtacatcaagtgtatcatatgccaagtacgccccctattgacgtcaatgacggtaaatggcccgcctggcattatgcccagtacatgaccttatgggactttcctacttggcagtacatctacgtattagtcatcgctattaccatggtcgaggtgagccccacgttctgcttcactctccccatctcccccccctccccacccccaattttgtatttatttattttttaattattttgtgcagcgatgggggcggggggggggggggggcgcgcgccaggcggggcggggcggggcgaggggcggggcggggcgaggcggagaggtgcggcggcagccaatcagagcggcgcgctccgaaagtttccttttatggcgaggcggcggcggcggcggccctataaaaagcgaagcgcgcggcgggcgggagtcgctgcgcgctgccttcgccccgtgccccgctccgccgccgcctcgcgccgcccgccccggctctgactgaccgcgttactcccacaggtgagcgggcgggacggcccttctcctccgggctgtaattagcgcttggtttaatgacggcttgtttcttttctgtggctgcgtgaaagccttgaggggctccgggagggccctttgtgcggggggagcggctcggggggtgcgtgcgtgtgtgtgtgcgtggggagcgccgcgtgcggctccgcgctgcccggcggctgtgagcgctgcgggcgcggcgcggggctttgtgcgctccgcagtgtgcgcgaggggagcgcggccgggggcggtgccccgcggtgcggggggggctgcgaggggaacaaaggctgcgtgcggggtgtgtgcgtgggggggtgagcagggggtgtgggcgcgtcggtcgggctgcaaccccccctgcacccccctccccgagttgctgagcacggcccggcttcgggtgcggggctccgtacggggcgtggcgcggggctcgccgtgccgggcggggggtggcggcaggtgggggtgccgggcggggcggggccgcctcgggccggggagggctcgggggaggggcgcggcggcccccggagcgccggcggctgtcgaggcgcggcgagccgcagccattgccttttatggtaatcgtgcgagagggcgcagggacttcctttgtcccaaatctgtgcggagccgaaatctgggaggcgccgccgcaccccctctagcgggcgcggggcgaagcggtgcggcgccggcaggaaggaaatgggcggggagggccttcgtgcgtcgccgcgccgccgtccccttctccctctccagcctcggggctgtccgcggggggacggctgccttcgggggggacggggcagggcggggttcggcttctggcgtgtgaccggcggctctagagcctctgctaaccatgttcatgccttcttctttttcctacagctcctgggcaacgtgctggttattgtgctgtctcatcattttggcaaagaattgcggccgtctcaggccgaattcaagcttgccaccatggggatccttcccagccctgggatgcctgcgctgctctccctcgtgagccttctctccgtgctgctgatgggttgcgtagctgaaaccggtcagctagcctgcaaggaagattacaggtacgcaatatcatcaaccaatgagatagggctactcggggccggaggtctcaccaccacctggaaagaatacaaccacgatttgcaactgaatgacgggaccgttaaggccatttgcgtggcaggttcctttaaagtcacagcacttaatgtggtcagtaggaggtatttggcatcattgcataaggaggctttacccacttccgtgacattc**GAGCTC**ctgttcgacgggaccaacccatcaactgaggaaatgggagatgacttcgggttcgggctgtgcccgtttgatacgagtcctgttgtaagagggaagttcaatacaacgctgctgaacggaccggccttccagatggtatgccccataggatggacagggactgtaagctgcacagcagtgagcccaacaactctgagaacagaagtggtaaagaccttcaggagggacaagccctttccgcacagaatggattgtgtgaccacaacagtggaaaatgaagatttattctactgtaagttggggggcaactggacatgtgtgaaaggtgaaccagtggtctacacgggggggctagtaaaacaatgcagatggtgtggctttgacttcaatgagcctgacggactcccacactaccccataggtaagtgcattttggcaaatgagacaggttacagaatagtggattcaacagactgtaacagagacggtgttgtaatcagcacagaggggagtcatgagtgcttgatcggtaacacaactgtcaaggtgcatgcatcagatgaaagactgggccccatgccatgcagacctaaagagatcgtctctagtgcaggacctgtaaggaaaacttcctgtacattcaactacgcaaaaactttgaagaacaagtactatgagcccagggacagctacttccagcaatatatgcttaagggcgagtatcagtactggtttgacctggacgtgactgaccgccactcaggtaccggctcctccggactgaatgacatctttgaggcccagaagatcgagtggcacgagtccggcggcggatctcttgtaccgcgaggtagttggtcccaccctcagttcgagaagggcggaggctccggcggcggatctggaggaggctcctggtcccacccccagttcgagaaatgataactcgagactagtatcgcgataattcactcctcaggtgcaggctgcctatcagaaggtggtggctggtgtggccaatgccctggctcacaaataccactgagatctttttccctctgccaaaaattatggggacatcatgaagccccttgagcatctgacttctggctaataaaggaaatttattttcattgcaatagtgtgttggaattttttgtgtctctcactcggaaggacatatgggagggcaaatcatttaaaacatcagaatgagtatttggtttagagtttggcaacatatgcccatatgctggctgccatgaacaaaggttggctataaagaggtcatcagtatatgaaacagccccctgctgtccattccttattccatagaaaagccttgacttgaggttagattttttttatattttgttttgtgttatttttttctttaacatccctaaaattttccttacatgttttactagccagatttttcctcctctcctgactactcccagtcatagctgtccctcttctcttatggagatccctcgacctgcagcccaagcttggcgtaatcatggtcatagctgtttcctgtgtgaaattgttatccgctcacaattccacacaacatacgagccggaagcataaagtgtaaagcctggggtgcctaatgagtgagctaactcacattaattgcgttgcgctcactgcccgctttccagtcgggaaacctgtcgtgccagcggatcgatccgctgcattaatgaatcggccaacgcgcggggagaggcggtttgcgtattgggcgctcttccgcttcctcgctcactgactcgctgcgctcggtcgttcggctgcggcgagcggtatcagctcactcaaaggcggtaatacggttatccacagaatcaggggataacgcaggaaagaacatgtgagcaaaaggccagcaaaaggccaggaaccgtaaaaaggccgcgttgctggcgtttttccataggctccgcccccctgacgagcatcacaaaaatcgacgctcaagtcagaggtggcgaaacccgacaggactataaagataccaggcgtttccccctggaagctccctcgtgcgctctcctgttccgaccctgccgcttaccggatacctgtccgcctttctcccttcgggaagcgtggcgctttctcatagctcacgctgtaggtatctcagttcggtgtaggtcgttcgctccaagctgggctgtgtgcacgaaccccccgttcagcccgaccgctgcgccttatccggtaactatcgtcttgagtccaacccggtaagacacgacttatcgccactggcagcagccactggtaacaggattagcagagcgaggtatgtaggcggtgctacagagttcttgaagtggtggcctaactacggctacactagaagaacagtatttggtatctgcgctctgctgaagccagttaccttcggaaaaagagttggtagctcttgatccggcaaacaaaccaccgctggtagcggtggtttttttgtttgcaagcagcagattacgcgcagaaaaaaaggatctcaagaagatcctttgatcttttctacggggtctgacgctcagtggaacgaaaactcacgttaagggattttggtcatgagattatcaaaaaggatcttcacctagatccttttaaattaaaaatgaagttttaaatcaatctaaagtatatatgagtaaacttggtctgacagttaccaatgcttaatcagtgaggcacctatctcagcgatctgtctatttcgttcatccatagttgcctgactccccgtcgtgtagataactacgatacgggagggcttaccatctggccccagtgctgcaatgataccgcgagacccacgctcaccggctccagatttatcagcaataaaccagccagccggaagggccgagcgcagaagtggtcctgcaactttatccgcctccatccagtctattaattgttgccgggaagctagagtaagtagttcgccagttaatagtttgcgcaacgttgttgccattgctacaggcatcgtggtgtcacgctcgtcgtttggtatggcttcattcagctccggttcccaacgatcaaggcgagttacatgatcccccatgttgtgcaaaaaagcggttagctccttcggtcctccgatcgttgtcagaagtaagttggccgcagtgttatcactcatggttatggcagcactgcataattctcttactgtcatgccatccgtaagatgcttttctgtgactggtgagtactcaaccaagtcattctgagaatagtgtatgcggcgaccgagttgctcttgcccggcgtcaatacgggataataccgcgccacatagcagaactttaaaagtgctcatcattggaaaacgttcttcggggcgaaaactctcaaggatcttaccgctgttgagatccagttcgatgtaacccactcgtgcacccaactgatcttcagcatcttttactttcaccagcgtttctgggtgagcaaaaacaggaaggcaaaatgccgcaaaaaagggaataagggcgacacggaaatgttgaatactcatactcttcctttttcaatattattgaagcatttatcagggttattgtctcatgagcggatacatatttgaatgtatttagaaaaataaacaaataggggttccgcgcacatttccccgaaaagtgccacctgg

**pBVDV E2-*p*CSFV-B-C.**

gtcgacattgattattgactagttattaatagtaatcaattacggggtcattagttcatagcccatatatggagttccgcgttacataacttacggtaaatggcccgcctggctgaccgcccaacgacccccgcccattgacgtcaataatgacgtatgttcccatagtaacgccaatagggactttccattgacgtcaatgggtggagtatttacggtaaactgcccacttggcagtacatcaagtgtatcatatgccaagtacgccccctattgacgtcaatgacggtaaatggcccgcctggcattatgcccagtacatgaccttatgggactttcctacttggcagtacatctacgtattagtcatcgctattaccatggtcgaggtgagccccacgttctgcttcactctccccatctcccccccctccccacccccaattttgtatttatttattttttaattattttgtgcagcgatgggggcggggggggggggggggcgcgcgccaggcggggcggggcggggcgaggggcggggcggggcgaggcggagaggtgcggcggcagccaatcagagcggcgcgctccgaaagtttccttttatggcgaggcggcggcggcggcggccctataaaaagcgaagcgcgcggcgggcgggagtcgctgcgcgctgccttcgccccgtgccccgctccgccgccgcctcgcgccgcccgccccggctctgactgaccgcgttactcccacaggtgagcgggcgggacggcccttctcctccgggctgtaattagcgcttggtttaatgacggcttgtttcttttctgtggctgcgtgaaagccttgaggggctccgggagggccctttgtgcggggggagcggctcggggggtgcgtgcgtgtgtgtgtgcgtggggagcgccgcgtgcggctccgcgctgcccggcggctgtgagcgctgcgggcgcggcgcggggctttgtgcgctccgcagtgtgcgcgaggggagcgcggccgggggcggtgccccgcggtgcggggggggctgcgaggggaacaaaggctgcgtgcggggtgtgtgcgtgggggggtgagcagggggtgtgggcgcgtcggtcgggctgcaaccccccctgcacccccctccccgagttgctgagcacggcccggcttcgggtgcggggctccgtacggggcgtggcgcggggctcgccgtgccgggcggggggtggcggcaggtgggggtgccgggcggggcggggccgcctcgggccggggagggctcgggggaggggcgcggcggcccccggagcgccggcggctgtcgaggcgcggcgagccgcagccattgccttttatggtaatcgtgcgagagggcgcagggacttcctttgtcccaaatctgtgcggagccgaaatctgggaggcgccgccgcaccccctctagcgggcgcggggcgaagcggtgcggcgccggcaggaaggaaatgggcggggagggccttcgtgcgtcgccgcgccgccgtccccttctccctctccagcctcggggctgtccgcggggggacggctgccttcgggggggacggggcagggcggggttcggcttctggcgtgtgaccggcggctctagagcctctgctaaccatgttcatgccttcttctttttcctacagctcctgggcaacgtgctggttattgtgctgtctcatcattttggcaaagaattgcggccgtctcaggccgaattcaagcttgccaccatggggatccttcccagccctgggatgcctgcgctgctctccctcgtgagccttctctccgtgctgctgatgggttgcgtagctgaa**ACCGGT**cacttggattgcaaacctgaattctcgtatgccatagcaaaggacgaaagaattggtcaactgggggctgaaggccttaccaccacttggaaggaatactcacctggaatgaagctggaagacacaatggtcattgcttggtgcgaagatgggaagttaatgtacctccaaagatgcacgagagaaaccagatatctcgcaatcttgcatacaagagccttgccgaccagtgtggtattcaaaaaactctttgatgGGCGAAAGCAAGAGGATGTAGTCGAAATGAACGACAACTTTGAATTTGGACTCTGCCCATGTGATGCCAAACCCATAGtcaagggaaagtacaatacaaccttgttgaacggtagtgctttctatcttgtctgtccaatagggtggacgggtgttatagagtgcacagcagtgagcccaacaactctgagaacagaagtggtaaagaccttcaggagggacaagccctttccgcacagaatggattgtgtgaccacaacagtggaaaatgaggatctccataactgcatccttggaggaaattggacttgtgtgcctggagaccaactactatacaaagggggctctattgaatcttgcaagtggtgtggctatcaatttaaagagagtgagggactaccacactaccccattggcaagtgtaaattggagaacgagactggttacaggctagtagacagtacctcttgcaatagagaaggtgtggccatagtaccacaagggacattaaagtgcaagataggaaaaacaactgtacaggtcatagctatggataccaaactcggacctatgccttgcagaccatatgaaatcatatcaagtgaggggcctgtagaaaagacagcgtgtactttcaactacactaagacattaaaaaataagtattttgagcccagagacagctactttcagcaatacatgctaaaaggagagtatcaatactggtttgacctggaggtgactgaccatcaccgg**GGTACC**ggctcctccggactgaatgacatctttgaggcccagaagatcgagtggcacgagtccggcggcggatctcttgtaccgcgaggtagttggtcccaccctcagttcgagaagggcggaggctccggcggcggatctggaggaggctcctggtcccacccccagttcgagaaatgataactcgagactagtatcgcgataattcactcctcaggtgcaggctgcctatcagaaggtggtggctggtgtggccaatgccctggctcacaaataccactgagatctttttccctctgccaaaaattatggggacatcatgaagccccttgagcatctgacttctggctaataaaggaaatttattttcattgcaatagtgtgttggaattttttgtgtctctcactcggaaggacatatgggagggcaaatcatttaaaacatcagaatgagtatttggtttagagtttggcaacatatgcccatatgctggctgccatgaacaaaggttggctataaagaggtcatcagtatatgaaacagccccctgctgtccattccttattccatagaaaagccttgacttgaggttagattttttttatattttgttttgtgttatttttttctttaacatccctaaaattttccttacatgttttactagccagatttttcctcctctcctgactactcccagtcatagctgtccctcttctcttatggagatccctcgacctgcagcccaagcttggcgtaatcatggtcatagctgtttcctgtgtgaaattgttatccgctcacaattccacacaacatacgagccggaagcataaagtgtaaagcctggggtgcctaatgagtgagctaactcacattaattgcgttgcgctcactgcccgctttccagtcgggaaacctgtcgtgccagcggatcgatccgctgcattaatgaatcggccaacgcgcggggagaggcggtttgcgtattgggcgctcttccgcttcctcgctcactgactcgctgcgctcggtcgttcggctgcggcgagcggtatcagctcactcaaaggcggtaatacggttatccacagaatcaggggataacgcaggaaagaacatgtgagcaaaaggccagcaaaaggccaggaaccgtaaaaaggccgcgttgctggcgtttttccataggctccgcccccctgacgagcatcacaaaaatcgacgctcaagtcagaggtggcgaaacccgacaggactataaagataccaggcgtttccccctggaagctccctcgtgcgctctcctgttccgaccctgccgcttaccggatacctgtccgcctttctcccttcgggaagcgtggcgctttctcatagctcacgctgtaggtatctcagttcggtgtaggtcgttcgctccaagctgggctgtgtgcacgaaccccccgttcagcccgaccgctgcgccttatccggtaactatcgtcttgagtccaacccggtaagacacgacttatcgccactggcagcagccactggtaacaggattagcagagcgaggtatgtaggcggtgctacagagttcttgaagtggtggcctaactacggctacactagaagaacagtatttggtatctgcgctctgctgaagccagttaccttcggaaaaagagttggtagctcttgatccggcaaacaaaccaccgctggtagcggtggtttttttgtttgcaagcagcagattacgcgcagaaaaaaaggatctcaagaagatcctttgatcttttctacggggtctgacgctcagtggaacgaaaactcacgttaagggattttggtcatgagattatcaaaaaggatcttcacctagatccttttaaattaaaaatgaagttttaaatcaatctaaagtatatatgagtaaacttggtctgacagttaccaatgcttaatcagtgaggcacctatctcagcgatctgtctatttcgttcatccatagttgcctgactccccgtcgtgtagataactacgatacgggagggcttaccatctggccccagtgctgcaatgataccgcgagacccacgctcaccggctccagatttatcagcaataaaccagccagccggaagggccgagcgcagaagtggtcctgcaactttatccgcctccatccagtctattaattgttgccgggaagctagagtaagtagttcgccagttaatagtttgcgcaacgttgttgccattgctacaggcatcgtggtgtcacgctcgtcgtttggtatggcttcattcagctccggttcccaacgatcaaggcgagttacatgatcccccatgttgtgcaaaaaagcggttagctccttcggtcctccgatcgttgtcagaagtaagttggccgcagtgttatcactcatggttatggcagcactgcataattctcttactgtcatgccatccgtaagatgcttttctgtgactggtgagtactcaaccaagtcattctgagaatagtgtatgcggcgaccgagttgctcttgcccggcgtcaatacgggataataccgcgccacatagcagaactttaaaagtgctcatcattggaaaacgttcttcggggcgaaaactctcaaggatcttaccgctgttgagatccagttcgatgtaacccactcgtgcacccaactgatcttcagcatcttttactttcaccagcgtttctgggtgagcaaaaacaggaaggcaaaatgccgcaaaaaagggaataagggcgacacggaaatgttgaatactcatactcttcctttttcaatattattgaagcatttatcagggttattgtctcatgagcggatacatatttgaatgtatttagaaaaataaacaaataggggttccgcgcacatttccccgaaaagtgccacctgg

**pCSFV E2-pBVDV-A-B.**

gtcgacattgattattgactagttattaatagtaatcaattacggggtcattagttcatagcccatatatggagttccgcgttacataacttacggtaaatggcccgcctggctgaccgcccaacgacccccgcccattgacgtcaataatgacgtatgttcccatagtaacgccaatagggactttccattgacgtcaatgggtggagtatttacggtaaactgcccacttggcagtacatcaagtgtatcatatgccaagtacgccccctattgacgtcaatgacggtaaatggcccgcctggcattatgcccagtacatgaccttatgggactttcctacttggcagtacatctacgtattagtcatcgctattaccatggtcgaggtgagccccacgttctgcttcactctccccatctcccccccctccccacccccaattttgtatttatttattttttaattattttgtgcagcgatgggggcggggggggggggggggcgcgcgccaggcggggcggggcggggcgaggggcggggcggggcgaggcggagaggtgcggcggcagccaatcagagcggcgcgctccgaaagtttccttttatggcgaggcggcggcggcggcggccctataaaaagcgaagcgcgcggcgggcgggagtcgctgcgcgctgccttcgccccgtgccccgctccgccgccgcctcgcgccgcccgccccggctctgactgaccgcgttactcccacaggtgagcgggcgggacggcccttctcctccgggctgtaattagcgcttggtttaatgacggcttgtttcttttctgtggctgcgtgaaagccttgaggggctccgggagggccctttgtgcggggggagcggctcggggggtgcgtgcgtgtgtgtgtgcgtggggagcgccgcgtgcggctccgcgctgcccggcggctgtgagcgctgcgggcgcggcgcggggctttgtgcgctccgcagtgtgcgcgaggggagcgcggccgggggcggtgccccgcggtgcggggggggctgcgaggggaacaaaggctgcgtgcggggtgtgtgcgtgggggggtgagcagggggtgtgggcgcgtcggtcgggctgcaaccccccctgcacccccctccccgagttgctgagcacggcccggcttcgggtgcggggctccgtacggggcgtggcgcggggctcgccgtgccgggcggggggtggcggcaggtgggggtgccgggcggggcggggccgcctcgggccggggagggctcgggggaggggcgcggcggcccccggagcgccggcggctgtcgaggcgcggcgagccgcagccattgccttttatggtaatcgtgcgagagggcgcagggacttcctttgtcccaaatctgtgcggagccgaaatctgggaggcgccgccgcaccccctctagcgggcgcggggcgaagcggtgcggcgccggcaggaaggaaatgggcggggagggccttcgtgcgtcgccgcgccgccgtccccttctccctctccagcctcggggctgtccgcggggggacggctgccttcgggggggacggggcagggcggggttcggcttctggcgtgtgaccggcggctctagagcctctgctaaccatgttcatgccttcttctttttcctacagctcctgggcaacgtgctggttattgtgctgtctcatcattttggcaaagaattgcggccgtctcaggccgaattcaagcttgccaccatggggatccttcccagccctgggatgcctgcgctgctctccctcgtgagccttctctccgtgctgctgatgggttgcgtagctgaa**ACCGGT**cagctagcctgcaaggaagattacaggtacgcaatatcatcaaccaatgagatagggctactcggggccggaggtctcaccaccacctggaaagaatacaaccacgatttgcaactgaatgacgggaccgttaaggccatttgcgtggcaggttcctttaaagtcacagcaagatgcacgagagaaaccagatatctcgcaatcttgcatacaagagccttgccgaccagtgtggtattcaaaaaactctttgatgggcgaaagcaagaggatgtagtcgaaatgaacgacaactttgaatttggactctgcccatgtgatgccaaacccatagtcaagggaaagtacaatacaaccttgttgaacggtagtgctttctatcttgtctgtccaatagggtggacgggtgttatagagtgcacagcagtgagcccaacaactctgagaacagaagtggtaaagaccttcaggagggacaagccctttccgcacagaatggattgtgtgaccacaacagtggaaaatgaagatttattctactgtaagttggggggcaactggacatgtgtgaaaggtgaaccagtggtctacacgggggggctagtaaaacaatgcagatggtgtggctttgacttcaatgagcctgacggactcccacactaccccataggtaagtgcattttggcaaatgagacaggttacagaatagtggattcaacagactgtaacagagacggtgttgtaatcagcacagaggggagtcatgagtgcttgatcggtaacacaactgtcaaggtgcatgcatcagatgaaagactgggccccatgccatgcagacctaaagagatcgtctctagtgcaggacctgtaaggaaaacttcctgtacattcaactacgcaaaaactttgaagaacaagtactatgagcccagggacagctacttccagcaatatatgcttaagggcgagtatcagtactggtttgacctggacgtgactgaccgccactca**GGTACC**ggctcctccggactgaatgacatctttgaggcccagaagatcgagtggcacgagtccggcggcggatctcttgtaccgcgaggtagttggtcccaccctcagttcgagaagggcggaggctccggcggcggatctggaggaggctcctggtcccacccccagttcgagaaatgataactcgagactagtatcgcgataattcactcctcaggtgcaggctgcctatcagaaggtggtggctggtgtggccaatgccctggctcacaaataccactgagatctttttccctctgccaaaaattatggggacatcatgaagccccttgagcatctgacttctggctaataaaggaaatttattttcattgcaatagtgtgttggaattttttgtgtctctcactcggaaggacatatgggagggcaaatcatttaaaacatcagaatgagtatttggtttagagtttggcaacatatgcccatatgctggctgccatgaacaaaggttggctataaagaggtcatcagtatatgaaacagccccctgctgtccattccttattccatagaaaagccttgacttgaggttagattttttttatattttgttttgtgttatttttttctttaacatccctaaaattttccttacatgttttactagccagatttttcctcctctcctgactactcccagtcatagctgtccctcttctcttatggagatccctcgacctgcagcccaagcttggcgtaatcatggtcatagctgtttcctgtgtgaaattgttatccgctcacaattccacacaacatacgagccggaagcataaagtgtaaagcctggggtgcctaatgagtgagctaactcacattaattgcgttgcgctcactgcccgctttccagtcgggaaacctgtcgtgccagcggatcgatccgctgcattaatgaatcggccaacgcgcggggagaggcggtttgcgtattgggcgctcttccgcttcctcgctcactgactcgctgcgctcggtcgttcggctgcggcgagcggtatcagctcactcaaaggcggtaatacggttatccacagaatcaggggataacgcaggaaagaacatgtgagcaaaaggccagcaaaaggccaggaaccgtaaaaaggccgcgttgctggcgtttttccataggctccgcccccctgacgagcatcacaaaaatcgacgctcaagtcagaggtggcgaaacccgacaggactataaagataccaggcgtttccccctggaagctccctcgtgcgctctcctgttccgaccctgccgcttaccggatacctgtccgcctttctcccttcgggaagcgtggcgctttctcatagctcacgctgtaggtatctcagttcggtgtaggtcgttcgctccaagctgggctgtgtgcacgaaccccccgttcagcccgaccgctgcgccttatccggtaactatcgtcttgagtccaacccggtaagacacgacttatcgccactggcagcagccactggtaacaggattagcagagcgaggtatgtaggcggtgctacagagttcttgaagtggtggcctaactacggctacactagaagaacagtatttggtatctgcgctctgctgaagccagttaccttcggaaaaagagttggtagctcttgatccggcaaacaaaccaccgctggtagcggtggtttttttgtttgcaagcagcagattacgcgcagaaaaaaaggatctcaagaagatcctttgatcttttctacggggtctgacgctcagtggaacgaaaactcacgttaagggattttggtcatgagattatcaaaaaggatcttcacctagatccttttaaattaaaaatgaagttttaaatcaatctaaagtatatatgagtaaacttggtctgacagttaccaatgcttaatcagtgaggcacctatctcagcgatctgtctatttcgttcatccatagttgcctgactccccgtcgtgtagataactacgatacgggagggcttaccatctggccccagtgctgcaatgataccgcgagacccacgctcaccggctccagatttatcagcaataaaccagccagccggaagggccgagcgcagaagtggtcctgcaactttatccgcctccatccagtctattaattgttgccgggaagctagagtaagtagttcgccagttaatagtttgcgcaacgttgttgccattgctacaggcatcgtggtgtcacgctcgtcgtttggtatggcttcattcagctccggttcccaacgatcaaggcgagttacatgatcccccatgttgtgcaaaaaagcggttagctccttcggtcctccgatcgttgtcagaagtaagttggccgcagtgttatcactcatggttatggcagcactgcataattctcttactgtcatgccatccgtaagatgcttttctgtgactggtgagtactcaaccaagtcattctgagaatagtgtatgcggcgaccgagttgctcttgcccggcgtcaatacgggataataccgcgccacatagcagaactttaaaagtgctcatcattggaaaacgttcttcggggcgaaaactctcaaggatcttaccgctgttgagatccagttcgatgtaacccactcgtgcacccaactgatcttcagcatcttttactttcaccagcgtttctgggtgagcaaaaacaggaaggcaaaatgccgcaaaaaagggaataagggcgacacggaaatgttgaatactcatactcttcctttttcaatattattgaagcatttatcagggttattgtctcatgagcggatacatatttgaatgtatttagaaaaataaacaaataggggttccgcgcacatttccccgaaaagtgccacctgg

**pCSFV E2-pBVDV-A.**

gtcgacattgattattgactagttattaatagtaatcaattacggggtcattagttcatagcccatatatggagttccgcgttacataacttacggtaaatggcccgcctggctgaccgcccaacgacccccgcccattgacgtcaataatgacgtatgttcccatagtaacgccaatagggactttccattgacgtcaatgggtggagtatttacggtaaactgcccacttggcagtacatcaagtgtatcatatgccaagtacgccccctattgacgtcaatgacggtaaatggcccgcctggcattatgcccagtacatgaccttatgggactttcctacttggcagtacatctacgtattagtcatcgctattaccatggtcgaggtgagccccacgttctgcttcactctccccatctcccccccctccccacccccaattttgtatttatttattttttaattattttgtgcagcgatgggggcggggggggggggggggcgcgcgccaggcggggcggggcggggcgaggggcggggcggggcgaggcggagaggtgcggcggcagccaatcagagcggcgcgctccgaaagtttccttttatggcgaggcggcggcggcggcggccctataaaaagcgaagcgcgcggcgggcgggagtcgctgcgcgctgccttcgccccgtgccccgctccgccgccgcctcgcgccgcccgccccggctctgactgaccgcgttactcccacaggtgagcgggcgggacggcccttctcctccgggctgtaattagcgcttggtttaatgacggcttgtttcttttctgtggctgcgtgaaagccttgaggggctccgggagggccctttgtgcggggggagcggctcggggggtgcgtgcgtgtgtgtgtgcgtggggagcgccgcgtgcggctccgcgctgcccggcggctgtgagcgctgcgggcgcggcgcggggctttgtgcgctccgcagtgtgcgcgaggggagcgcggccgggggcggtgccccgcggtgcggggggggctgcgaggggaacaaaggctgcgtgcggggtgtgtgcgtgggggggtgagcagggggtgtgggcgcgtcggtcgggctgcaaccccccctgcacccccctccccgagttgctgagcacggcccggcttcgggtgcggggctccgtacggggcgtggcgcggggctcgccgtgccgggcggggggtggcggcaggtgggggtgccgggcggggcggggccgcctcgggccggggagggctcgggggaggggcgcggcggcccccggagcgccggcggctgtcgaggcgcggcgagccgcagccattgccttttatggtaatcgtgcgagagggcgcagggacttcctttgtcccaaatctgtgcggagccgaaatctgggaggcgccgccgcaccccctctagcgggcgcggggcgaagcggtgcggcgccggcaggaaggaaatgggcggggagggccttcgtgcgtcgccgcgccgccgtccccttctccctctccagcctcggggctgtccgcggggggacggctgccttcgggggggacggggcagggcggggttcggcttctggcgtgtgaccggcggctctagagcctctgctaaccatgttcatgccttcttctttttcctacagctcctgggcaacgtgctggttattgtgctgtctcatcattttggcaaagaattgcggccgtctcaggccgaattcaagcttgccaccatggggatccttcccagccctgggatgcctgcgctgctctccctcgtgagccttctctccgtgctgctgatgggttgcgtagctgaa**ACCGGT**cagctagcctgcaaggaagattacaggtacgcaatatcatcaaccaatgagatagggctactcggggccggaggtctcaccaccacctggaaagaatacaaccacgatttgcaactgaatgacgggaccgttaaggccatttgcgtggcaggttcctttaaagtcacagcacttaatgtgagagaaaccagatatctcgcaatcttgcatacaagagccttgccgaccagtgtggtattcaaaaaactctttgatgggcgaaagcaatcaactgaggaaatgggagatgacttcgggttcgggctgtgcccgtttgatacgagtcctgttgtcaagggaaagtacaatacaaccttgttgaacggtagtgctttctatcttgtctgtccaatagggtggacgggtgttatagagtgcacagcagtgagcccaacaactctgagaacagaagtggtaaaga**CCTTCAGGAGG**gacaagccctttccgcacagaatggattgtgtgaccacaacagtggaaaatgaagatttattctactgtaagttggggggcaactggacatgtgtgaaaggtgaaccagtggtctacacgggggggctagtaaaacaatgcagatggtgtggctttgacttcaatgagcctgacggactcccacactaccccataggtaagtgcattttggcaaatgagacaggttacagaatagtggattcaacagactgtaacagagacggtgttgtaatcagcacagaggggagtcatgagtgcttgatcggtaacacaactgtcaaggtgcatgcatcagatgaaagactgggccccatgccatgcagacctaaagagatcgtctctagtgcaggacctgtaaggaaaacttcctgtacattcaactacgcaaaaactttgaagaacaagtactatgagcccagggacagctacttccagcaatatatgcttaagggcgagtatcagtactggtttgacctggacgtgactgaccgccactcaggtaccggctcctccggactgaatgacatctttgaggcccagaagatcgagtggcacgagtccggcggcggatctcttgtaccgcgaggtagttggtcccaccctcagttcgagaagggcggaggctccggcggcggatctggaggaggctcctggtcccacccccagttcgagaaatgataactcgagactagtatcgcgataattcactcctcaggtgcaggctgcctatcagaaggtggtggctggtgtggccaatgccctggctcacaaataccactgagatctttttccctctgccaaaaattatggggacatcatgaagccccttgagcatctgacttctggctaataaaggaaatttattttcattgcaatagtgtgttggaattttttgtgtctctcactcggaaggacatatgggagggcaaatcatttaaaacatcagaatgagtatttggtttagagtttggcaacatatgcccatatgctggctgccatgaacaaaggttggctataaagaggtcatcagtatatgaaacagccccctgctgtccattccttattccatagaaaagccttgacttgaggttagattttttttatattttgttttgtgttatttttttctttaacatccctaaaattttccttacatgttttactagccagatttttcctcctctcctgactactcccagtcatagctgtccctcttctcttatggagatccctcgacctgcagcccaagcttggcgtaatcatggtcatagctgtttcctgtgtgaaattgttatccgctcacaattccacacaacatacgagccggaagcataaagtgtaaagcctggggtgcctaatgagtgagctaactcacattaattgcgttgcgctcactgcccgctttccagtcgggaaacctgtcgtgccagcggatcgatccgctgcattaatgaatcggccaacgcgcggggagaggcggtttgcgtattgggcgctcttccgcttcctcgctcactgactcgctgcgctcggtcgttcggctgcggcgagcggtatcagctcactcaaaggcggtaatacggttatccacagaatcaggggataacgcaggaaagaacatgtgagcaaaaggccagcaaaaggccaggaaccgtaaaaaggccgcgttgctggcgtttttccataggctccgcccccctgacgagcatcacaaaaatcgacgctcaagtcagaggtggcgaaacccgacaggactataaagataccaggcgtttccccctggaagctccctcgtgcgctctcctgttccgaccctgccgcttaccggatacctgtccgcctttctcccttcgggaagcgtggcgctttctcatagctcacgctgtaggtatctcagttcggtgtaggtcgttcgctccaagctgggctgtgtgcacgaaccccccgttcagcccgaccgctgcgccttatccggtaactatcgtcttgagtccaacccggtaagacacgacttatcgccactggcagcagccactggtaacaggattagcagagcgaggtatgtaggcggtgctacagagttcttgaagtggtggcctaactacggctacactagaagaacagtatttggtatctgcgctctgctgaagccagttaccttcggaaaaagagttggtagctcttgatccggcaaacaaaccaccgctggtagcggtggtttttttgtttgcaagcagcagattacgcgcagaaaaaaaggatctcaagaagatcctttgatcttttctacggggtctgacgctcagtggaacgaaaactcacgttaagggattttggtcatgagattatcaaaaaggatcttcacctagatccttttaaattaaaaatgaagttttaaatcaatctaaagtatatatgagtaaacttggtctgacagttaccaatgcttaatcagtgaggcacctatctcagcgatctgtctatttcgttcatccatagttgcctgactccccgtcgtgtagataactacgatacgggagggcttaccatctggccccagtgctgcaatgataccgcgagacccacgctcaccggctccagatttatcagcaataaaccagccagccggaagggccgagcgcagaagtggtcctgcaactttatccgcctccatccagtctattaattgttgccgggaagctagagtaagtagttcgccagttaatagtttgcgcaacgttgttgccattgctacaggcatcgtggtgtcacgctcgtcgtttggtatggcttcattcagctccggttcccaacgatcaaggcgagttacatgatcccccatgttgtgcaaaaaagcggttagctccttcggtcctccgatcgttgtcagaagtaagttggccgcagtgttatcactcatggttatggcagcactgcataattctcttactgtcatgccatccgtaagatgcttttctgtgactggtgagtactcaaccaagtcattctgagaatagtgtatgcggcgaccgagttgctcttgcccggcgtcaatacgggataataccgcgccacatagcagaactttaaaagtgctcatcattggaaaacgttcttcggggcgaaaactctcaaggatcttaccgctgttgagatccagttcgatgtaacccactcgtgcacccaactgatcttcagcatcttttactttcaccagcgtttctgggtgagcaaaaacaggaaggcaaaatgccgcaaaaaagggaataagggcgacacggaaatgttgaatactcatactcttcctttttcaatattattgaagcatttatcagggttattgtctcatgagcggatacatatttgaatgtatttagaaaaataaacaaataggggttccgcgcacatttccccgaaaagtgccacctgg

**pCSFV E2-pBVDV-B-1.**

gtcgacattgattattgactagttattaatagtaatcaattacggggtcattagttcatagcccatatatggagttccgcgttacataacttacggtaaatggcccgcctggctgaccgcccaacgacccccgcccattgacgtcaataatgacgtatgttcccatagtaacgccaatagggactttccattgacgtcaatgggtggagtatttacggtaaactgcccacttggcagtacatcaagtgtatcatatgccaagtacgccccctattgacgtcaatgacggtaaatggcccgcctggcattatgcccagtacatgaccttatgggactttcctacttggcagtacatctacgtattagtcatcgctattaccatggtcgaggtgagccccacgttctgcttcactctccccatctcccccccctccccacccccaattttgtatttatttattttttaattattttgtgcagcgatgggggcggggggggggggggggcgcgcgccaggcggggcggggcggggcgaggggcggggcggggcgaggcggagaggtgcggcggcagccaatcagagcggcgcgctccgaaagtttccttttatggcgaggcggcggcggcggcggccctataaaaagcgaagcgcgcggcgggcgggagtcgctgcgcgctgccttcgccccgtgccccgctccgccgccgcctcgcgccgcccgccccggctctgactgaccgcgttactcccacaggtgagcgggcgggacggcccttctcctccgggctgtaattagcgcttggtttaatgacggcttgtttcttttctgtggctgcgtgaaagccttgaggggctccgggagggccctttgtgcggggggagcggctcggggggtgcgtgcgtgtgtgtgtgcgtggggagcgccgcgtgcggctccgcgctgcccggcggctgtgagcgctgcgggcgcggcgcggggctttgtgcgctccgcagtgtgcgcgaggggagcgcggccgggggcggtgccccgcggtgcggggggggctgcgaggggaacaaaggctgcgtgcggggtgtgtgcgtgggggggtgagcagggggtgtgggcgcgtcggtcgggctgcaaccccccctgcacccccctccccgagttgctgagcacggcccggcttcgggtgcggggctccgtacggggcgtggcgcggggctcgccgtgccgggcggggggtggcggcaggtgggggtgccgggcggggcggggccgcctcgggccggggagggctcgggggaggggcgcggcggcccccggagcgccggcggctgtcgaggcgcggcgagccgcagccattgccttttatggtaatcgtgcgagagggcgcagggacttcctttgtcccaaatctgtgcggagccgaaatctgggaggcgccgccgcaccccctctagcgggcgcggggcgaagcggtgcggcgccggcaggaaggaaatgggcggggagggccttcgtgcgtcgccgcgccgccgtccccttctccctctccagcctcggggctgtccgcggggggacggctgccttcgggggggacggggcagggcggggttcggcttctggcgtgtgaccggcggctctagagcctctgctaaccatgttcatgccttcttctttttcctacagctcctgggcaacgtgctggttattgtgctgtctcatcattttggcaaagaattgcggccgtctcaggccgaattcaagcttgccaccatggggatccttcccagccctgggatgcctgcgctgctctccctcgtgagccttctctccgtgctgctgatgggttgcgtagctgaaaccggtcagctagcctgcaaggaagattacaggtacgcaatatcatcaaccaatgagatagggctactcggggccggaggtctcaccaccacctggaaagaatacaaccacgatttgcaactgaatgacgggaccgttaaggccatttgcgtggcaggttcctttaaagtcacagcacttaatgtggtcagtaggaggtatttggcatcattgcataaggaggctttacccacttccgtgacattc**GAGCTC**ctgttcgacgggaccaacccagatgtagtcgaaatgaacgacaactttgaatttggactctgcccatgtgatgccaaacccatagtcaagggaaagtacaatacaaccttgttgaacggtagtgctttctatcttgtctgtccaatagggtggacgggtgttatagagtgcacagcagtgagcccaacaactctgagaacagaagtggtaaaga**CCTTCAGGAGG**gacaagccctttccgcacagaatggattgtgtgaccacaacagtggaaaatgaagatttattctactgtaagttggggggcaactggacatgtgtgaaaggtgaaccagtggtctacacgggggggctagtaaaacaatgcagatggtgtggctttgacttcaatgagcctgacggactcccacactaccccataggtaagtgcattttggcaaatgagacaggttacagaatagtggattcaacagactgtaacagagacggtgttgtaatcagcacagaggggagtcatgagtgcttgatcggtaacacaactgtcaaggtgcatgcatcagatgaaagactgggccccatgccatgcagacctaaagagatcgtctctagtgcaggacctgtaaggaaaacttcctgtacattcaactacgcaaaaactttgaagaacaagtactatgagcccagggacagctacttccagcaatatatgcttaagggcgagtatcagtactggtttgacctggacgtgactgaccgccactcaggtaccggctcctccggactgaatgacatctttgaggcccagaagatcgagtggcacgagtccggcggcggatctcttgtaccgcgaggtagttggtcccaccctcagttcgagaagggcggaggctccggcggcggatctggaggaggctcctggtcccacccccagttcgagaaatgataactcgagactagtatcgcgataattcactcctcaggtgcaggctgcctatcagaaggtggtggctggtgtggccaatgccctggctcacaaataccactgagatctttttccctctgccaaaaattatggggacatcatgaagccccttgagcatctgacttctggctaataaaggaaatttattttcattgcaatagtgtgttggaattttttgtgtctctcactcggaaggacatatgggagggcaaatcatttaaaacatcagaatgagtatttggtttagagtttggcaacatatgcccatatgctggctgccatgaacaaaggttggctataaagaggtcatcagtatatgaaacagccccctgctgtccattccttattccatagaaaagccttgacttgaggttagattttttttatattttgttttgtgttatttttttctttaacatccctaaaattttccttacatgttttactagccagatttttcctcctctcctgactactcccagtcatagctgtccctcttctcttatggagatccctcgacctgcagcccaagcttggcgtaatcatggtcatagctgtttcctgtgtgaaattgttatccgctcacaattccacacaacatacgagccggaagcataaagtgtaaagcctggggtgcctaatgagtgagctaactcacattaattgcgttgcgctcactgcccgctttccagtcgggaaacctgtcgtgccagcggatcgatccgctgcattaatgaatcggccaacgcgcggggagaggcggtttgcgtattgggcgctcttccgcttcctcgctcactgactcgctgcgctcggtcgttcggctgcggcgagcggtatcagctcactcaaaggcggtaatacggttatccacagaatcaggggataacgcaggaaagaacatgtgagcaaaaggccagcaaaaggccaggaaccgtaaaaaggccgcgttgctggcgtttttccataggctccgcccccctgacgagcatcacaaaaatcgacgctcaagtcagaggtggcgaaacccgacaggactataaagataccaggcgtttccccctggaagctccctcgtgcgctctcctgttccgaccctgccgcttaccggatacctgtccgcctttctcccttcgggaagcgtggcgctttctcatagctcacgctgtaggtatctcagttcggtgtaggtcgttcgctccaagctgggctgtgtgcacgaaccccccgttcagcccgaccgctgcgccttatccggtaactatcgtcttgagtccaacccggtaagacacgacttatcgccactggcagcagccactggtaacaggattagcagagcgaggtatgtaggcggtgctacagagttcttgaagtggtggcctaactacggctacactagaagaacagtatttggtatctgcgctctgctgaagccagttaccttcggaaaaagagttggtagctcttgatccggcaaacaaaccaccgctggtagcggtggtttttttgtttgcaagcagcagattacgcgcagaaaaaaaggatctcaagaagatcctttgatcttttctacggggtctgacgctcagtggaacgaaaactcacgttaagggattttggtcatgagattatcaaaaaggatcttcacctagatccttttaaattaaaaatgaagttttaaatcaatctaaagtatatatgagtaaacttggtctgacagttaccaatgcttaatcagtgaggcacctatctcagcgatctgtctatttcgttcatccatagttgcctgactccccgtcgtgtagataactacgatacgggagggcttaccatctggccccagtgctgcaatgataccgcgagacccacgctcaccggctccagatttatcagcaataaaccagccagccggaagggccgagcgcagaagtggtcctgcaactttatccgcctccatccagtctattaattgttgccgggaagctagagtaagtagttcgccagttaatagtttgcgcaacgttgttgccattgctacaggcatcgtggtgtcacgctcgtcgtttggtatggcttcattcagctccggttcccaacgatcaaggcgagttacatgatcccccatgttgtgcaaaaaagcggttagctccttcggtcctccgatcgttgtcagaagtaagttggccgcagtgttatcactcatggttatggcagcactgcataattctcttactgtcatgccatccgtaagatgcttttctgtgactggtgagtactcaaccaagtcattctgagaatagtgtatgcggcgaccgagttgctcttgcccggcgtcaatacgggataataccgcgccacatagcagaactttaaaagtgctcatcattggaaaacgttcttcggggcgaaaactctcaaggatcttaccgctgttgagatccagttcgatgtaacccactcgtgcacccaactgatcttcagcatcttttactttcaccagcgtttctgggtgagcaaaaacaggaaggcaaaatgccgcaaaaaagggaataagggcgacacggaaatgttgaatactcatactcttcctttttcaatattattgaagcatttatcagggttattgtctcatgagcggatacatatttgaatgtatttagaaaaataaacaaataggggttccgcgcacatttccccgaaaagtgccacctgg

**pCSFV-A.**

gtcgacattgattattgactagttattaatagtaatcaattacggggtcattagttcatagcccatatatggagttccgcgttacataacttacggtaaatggcccgcctggctgaccgcccaacgacccccgcccattgacgtcaataatgacgtatgttcccatagtaacgccaatagggactttccattgacgtcaatgggtggagtatttacggtaaactgcccacttggcagtacatcaagtgtatcatatgccaagtacgccccctattgacgtcaatgacggtaaatggcccgcctggcattatgcccagtacatgaccttatgggactttcctacttggcagtacatctacgtattagtcatcgctattaccatggtcgaggtgagccccacgttctgcttcactctccccatctcccccccctccccacccccaattttgtatttatttattttttaattattttgtgcagcgatgggggcggggggggggggggggcgcgcgccaggcggggcggggcggggcgaggggcggggcggggcgaggcggagaggtgcggcggcagccaatcagagcggcgcgctccgaaagtttccttttatggcgaggcggcggcggcggcggccctataaaaagcgaagcgcgcggcgggcgggagtcgctgcgcgctgccttcgccccgtgccccgctccgccgccgcctcgcgccgcccgccccggctctgactgaccgcgttactcccacaggtgagcgggcgggacggcccttctcctccgggctgtaattagcgcttggtttaatgacggcttgtttcttttctgtggctgcgtgaaagccttgaggggctccgggagggccctttgtgcggggggagcggctcggggggtgcgtgcgtgtgtgtgtgcgtggggagcgccgcgtgcggctccgcgctgcccggcggctgtgagcgctgcgggcgcggcgcggggctttgtgcgctccgcagtgtgcgcgaggggagcgcggccgggggcggtgccccgcggtgcggggggggctgcgaggggaacaaaggctgcgtgcggggtgtgtgcgtgggggggtgagcagggggtgtgggcgcgtcggtcgggctgcaaccccccctgcacccccctccccgagttgctgagcacggcccggcttcgggtgcggggctccgtacggggcgtggcgcggggctcgccgtgccgggcggggggtggcggcaggtgggggtgccgggcggggcggggccgcctcgggccggggagggctcgggggaggggcgcggcggcccccggagcgccggcggctgtcgaggcgcggcgagccgcagccattgccttttatggtaatcgtgcgagagggcgcagggacttcctttgtcccaaatctgtgcggagccgaaatctgggaggcgccgccgcaccccctctagcgggcgcggggcgaagcggtgcggcgccggcaggaaggaaatgggcggggagggccttcgtgcgtcgccgcgccgccgtccccttctccctctccagcctcggggctgtccgcggggggacggctgccttcgggggggacggggcagggcggggttcggcttctggcgtgtgaccggcggctctagagcctctgctaaccatgttcatgccttcttctttttcctacagctcctgggcaacgtgctggttattgtgctgtctcatcattttggcaaagaattgcggccgtctcaggccgaattcaagcttgccaccatggggatccttcccagccctgggatgcctgcgctgctctccctcgtgagccttctctccgtgctgctgatgggttgcgtagctgaa**ACCGGT**cagctagcctgcaaggaagattacaggtacgcaatatcatcaaccaatgagatagggctactcggggccggaggtctcaccaccacctggaaagaatacaaccacgatttgcaactgaatgacgggaccgttaaggccatttgcgtggcaggttcctttaaagtcacagcacttaatgtggtcagtaggaggtatttggcatcattgcataaggaggctttacccacttccgtgacattcgagctcctgttcgacgggacT**GGTACC**ggctcctccggactgaatgacatctttgaggcccagaagatcgagtggcacgagtccggcggcggatctcttgtaccgcgaggtagttggtcccaccctcagttcgagaagggcggaggctccggcggcggatctggaggaggctcctggtcccacccccagttcgagaaatgataactcgagactagtatcgcgataattcactcctcaggtgcaggctgcctatcagaaggtggtggctggtgtggccaatgccctggctcacaaataccactgagatctttttccctctgccaaaaattatggggacatcatgaagccccttgagcatctgacttctggctaataaaggaaatttattttcattgcaatagtgtgttggaattttttgtgtctctcactcggaaggacatatgggagggcaaatcatttaaaacatcagaatgagtatttggtttagagtttggcaacatatgcccatatgctggctgccatgaacaaaggttggctataaagaggtcatcagtatatgaaacagccccctgctgtccattccttattccatagaaaagccttgacttgaggttagattttttttatattttgttttgtgttatttttttctttaacatccctaaaattttccttacatgttttactagccagatttttcctcctctcctgactactcccagtcatagctgtccctcttctcttatggagatccctcgacctgcagcccaagcttggcgtaatcatggtcatagctgtttcctgtgtgaaattgttatccgctcacaattccacacaacatacgagccggaagcataaagtgtaaagcctggggtgcctaatgagtgagctaactcacattaattgcgttgcgctcactgcccgctttccagtcgggaaacctgtcgtgccagcggatcgatccgctgcattaatgaatcggccaacgcgcggggagaggcggtttgcgtattgggcgctcttccgcttcctcgctcactgactcgctgcgctcggtcgttcggctgcggcgagcggtatcagctcactcaaaggcggtaatacggttatccacagaatcaggggataacgcaggaaagaacatgtgagcaaaaggccagcaaaaggccaggaaccgtaaaaaggccgcgttgctggcgtttttccataggctccgcccccctgacgagcatcacaaaaatcgacgctcaagtcagaggtggcgaaacccgacaggactataaagataccaggcgtttccccctggaagctccctcgtgcgctctcctgttccgaccctgccgcttaccggatacctgtccgcctttctcccttcgggaagcgtggcgctttctcatagctcacgctgtaggtatctcagttcggtgtaggtcgttcgctccaagctgggctgtgtgcacgaaccccccgttcagcccgaccgctgcgccttatccggtaactatcgtcttgagtccaacccggtaagacacgacttatcgccactggcagcagccactggtaacaggattagcagagcgaggtatgtaggcggtgctacagagttcttgaagtggtggcctaactacggctacactagaagaacagtatttggtatctgcgctctgctgaagccagttaccttcggaaaaagagttggtagctcttgatccggcaaacaaaccaccgctggtagcggtggtttttttgtttgcaagcagcagattacgcgcagaaaaaaaggatctcaagaagatcctttgatcttttctacggggtctgacgctcagtggaacgaaaactcacgttaagggattttggtcatgagattatcaaaaaggatcttcacctagatccttttaaattaaaaatgaagttttaaatcaatctaaagtatatatgagtaaacttggtctgacagttaccaatgcttaatcagtgaggcacctatctcagcgatctgtctatttcgttcatccatagttgcctgactccccgtcgtgtagataactacgatacgggagggcttaccatctggccccagtgctgcaatgataccgcgagacccacgctcaccggctccagatttatcagcaataaaccagccagccggaagggccgagcgcagaagtggtcctgcaactttatccgcctccatccagtctattaattgttgccgggaagctagagtaagtagttcgccagttaatagtttgcgcaacgttgttgccattgctacaggcatcgtggtgtcacgctcgtcgtttggtatggcttcattcagctccggttcccaacgatcaaggcgagttacatgatcccccatgttgtgcaaaaaagcggttagctccttcggtcctccgatcgttgtcagaagtaagttggccgcagtgttatcactcatggttatggcagcactgcataattctcttactgtcatgccatccgtaagatgcttttctgtgactggtgagtactcaaccaagtcattctgagaatagtgtatgcggcgaccgagttgctcttgcccggcgtcaatacgggataataccgcgccacatagcagaactttaaaagtgctcatcattggaaaacgttcttcggggcgaaaactctcaaggatcttaccgctgttgagatccagttcgatgtaacccactcgtgcacccaactgatcttcagcatcttttactttcaccagcgtttctgggtgagcaaaaacaggaaggcaaaatgccgcaaaaaagggaataagggcgacacggaaatgttgaatactcatactcttcctttttcaatattattgaagcatttatcagggttattgtctcatgagcggatacatatttgaatgtatttagaaaaataaacaaataggggttccgcgcacatttccccgaaaagtgccacctgg

**pBVDV A.**

gtcgacattgattattgactagttattaatagtaatcaattacggggtcattagttcatagcccatatatggagttccgcgttacataacttacggtaaatggcccgcctggctgaccgcccaacgacccccgcccattgacgtcaataatgacgtatgttcccatagtaacgccaatagggactttccattgacgtcaatgggtggagtatttacggtaaactgcccacttggcagtacatcaagtgtatcatatgccaagtacgccccctattgacgtcaatgacggtaaatggcccgcctggcattatgcccagtacatgaccttatgggactttcctacttggcagtacatctacgtattagtcatcgctattaccatggtcgaggtgagccccacgttctgcttcactctccccatctcccccccctccccacccccaattttgtatttatttattttttaattattttgtgcagcgatgggggcggggggggggggggggcgcgcgccaggcggggcggggcggggcgaggggcggggcggggcgaggcggagaggtgcggcggcagccaatcagagcggcgcgctccgaaagtttccttttatggcgaggcggcggcggcggcggccctataaaaagcgaagcgcgcggcgggcgggagtcgctgcgcgctgccttcgccccgtgccccgctccgccgccgcctcgcgccgcccgccccggctctgactgaccgcgttactcccacaggtgagcgggcgggacggcccttctcctccgggctgtaattagcgcttggtttaatgacggcttgtttcttttctgtggctgcgtgaaagccttgaggggctccgggagggccctttgtgcggggggagcggctcggggggtgcgtgcgtgtgtgtgtgcgtggggagcgccgcgtgcggctccgcgctgcccggcggctgtgagcgctgcgggcgcggcgcggggctttgtgcgctccgcagtgtgcgcgaggggagcgcggccgggggcggtgccccgcggtgcggggggggctgcgaggggaacaaaggctgcgtgcggggtgtgtgcgtgggggggtgagcagggggtgtgggcgcgtcggtcgggctgcaaccccccctgcacccccctccccgagttgctgagcacggcccggcttcgggtgcggggctccgtacggggcgtggcgcggggctcgccgtgccgggcggggggtggcggcaggtgggggtgccgggcggggcggggccgcctcgggccggggagggctcgggggaggggcgcggcggcccccggagcgccggcggctgtcgaggcgcggcgagccgcagccattgccttttatggtaatcgtgcgagagggcgcagggacttcctttgtcccaaatctgtgcggagccgaaatctgggaggcgccgccgcaccccctctagcgggcgcggggcgaagcggtgcggcgccggcaggaaggaaatgggcggggagggccttcgtgcgtcgccgcgccgccgtccccttctccctctccagcctcggggctgtccgcggggggacggctgccttcgggggggacggggcagggcggggttcggcttctggcgtgtgaccggcggctctagagcctctgctaaccatgttcatgccttcttctttttcctacagctcctgggcaacgtgctggttattgtgctgtctcatcattttggcaaagaattgcggccgtctcaggccgaattcaagcttgccaccatggggatccttcccagccctgggatgcctgcgctgctctccctcgtgagccttctctccgtgctgctgatgggttgcgtagctgaa**ACCGGT**cacttggattgcaaacctgaattctcgtatgccatagcaaaggacgaaagaattggtcaactgggggctgaaggccttaccaccacttggaaggaatactcacctggaatgaagctggaagacacaatggtcattgcttggtgcgaagatgggaagttaatgtacctccaaagatgcacgagagaaaccagatatctcgcaatcttgcatacaagagccttgccgaccagtgtggtattcaaaaaactctttgatgggcga**GGTACC**ggctcctccggactgaatgacatctttgaggcccagaagatcgagtggcacgagtccggcggcggatctcttgtaccgcgaggtagttggtcccaccctcagttcgagaagggcggaggctccggcggcggatctggaggaggctcctggtcccacccccagttcgagaaatgataactcgagactagtatcgcgataattcactcctcaggtgcaggctgcctatcagaaggtggtggctggtgtggccaatgccctggctcacaaataccactgagatctttttccctctgccaaaaattatggggacatcatgaagccccttgagcatctgacttctggctaataaaggaaatttattttcattgcaatagtgtgttggaattttttgtgtctctcactcggaaggacatatgggagggcaaatcatttaaaacatcagaatgagtatttggtttagagtttggcaacatatgcccatatgctggctgccatgaacaaaggttggctataaagaggtcatcagtatatgaaacagccccctgctgtccattccttattccatagaaaagccttgacttgaggttagattttttttatattttgttttgtgttatttttttctttaacatccctaaaattttccttacatgttttactagccagatttttcctcctctcctgactactcccagtcatagctgtccctcttctcttatggagatccctcgacctgcagcccaagcttggcgtaatcatggtcatagctgtttcctgtgtgaaattgttatccgctcacaattccacacaacatacgagccggaagcataaagtgtaaagcctggggtgcctaatgagtgagctaactcacattaattgcgttgcgctcactgcccgctttccagtcgggaaacctgtcgtgccagcggatcgatccgctgcattaatgaatcggccaacgcgcggggagaggcggtttgcgtattgggcgctcttccgcttcctcgctcactgactcgctgcgctcggtcgttcggctgcggcgagcggtatcagctcactcaaaggcggtaatacggttatccacagaatcaggggataacgcaggaaagaacatgtgagcaaaaggccagcaaaaggccaggaaccgtaaaaaggccgcgttgctggcgtttttccataggctccgcccccctgacgagcatcacaaaaatcgacgctcaagtcagaggtggcgaaacccgacaggactataaagataccaggcgtttccccctggaagctccctcgtgcgctctcctgttccgaccctgccgcttaccggatacctgtccgcctttctcccttcgggaagcgtggcgctttctcatagctcacgctgtaggtatctcagttcggtgtaggtcgttcgctccaagctgggctgtgtgcacgaaccccccgttcagcccgaccgctgcgccttatccggtaactatcgtcttgagtccaacccggtaagacacgacttatcgccactggcagcagccactggtaacaggattagcagagcgaggtatgtaggcggtgctacagagttcttgaagtggtggcctaactacggctacactagaagaacagtatttggtatctgcgctctgctgaagccagttaccttcggaaaaagagttggtagctcttgatccggcaaacaaaccaccgctggtagcggtggtttttttgtttgcaagcagcagattacgcgcagaaaaaaaggatctcaagaagatcctttgatcttttctacggggtctgacgctcagtggaacgaaaactcacgttaagggattttggtcatgagattatcaaaaaggatcttcacctagatccttttaaattaaaaatgaagttttaaatcaatctaaagtatatatgagtaaacttggtctgacagttaccaatgcttaatcagtgaggcacctatctcagcgatctgtctatttcgttcatccatagttgcctgactccccgtcgtgtagataactacgatacgggagggcttaccatctggccccagtgctgcaatgataccgcgagacccacgctcaccggctccagatttatcagcaataaaccagccagccggaagggccgagcgcagaagtggtcctgcaactttatccgcctccatccagtctattaattgttgccgggaagctagagtaagtagttcgccagttaatagtttgcgcaacgttgttgccattgctacaggcatcgtggtgtcacgctcgtcgtttggtatggcttcattcagctccggttcccaacgatcaaggcgagttacatgatcccccatgttgtgcaaaaaagcggttagctccttcggtcctccgatcgttgtcagaagtaagttggccgcagtgttatcactcatggttatggcagcactgcataattctcttactgtcatgccatccgtaagatgcttttctgtgactggtgagtactcaaccaagtcattctgagaatagtgtatgcggcgaccgagttgctcttgcccggcgtcaatacgggataataccgcgccacatagcagaactttaaaagtgctcatcattggaaaacgttcttcggggcgaaaactctcaaggatcttaccgctgttgagatccagttcgatgtaacccactcgtgcacccaactgatcttcagcatcttttactttcaccagcgtttctgggtgagcaaaaacaggaaggcaaaatgccgcaaaaaagggaataagggcgacacggaaatgttgaatactcatactcttcctttttcaatattattgaagcatttatcagggttattgtctcatgagcggatacatatttgaatgtatttagaaaaataaacaaataggggttccgcgcacatttccccgaaaagtgccacctgg

**pCSFV-A-*p*BVDV-A.**

gtcgacattgattattgactagttattaatagtaatcaattacggggtcattagttcatagcccatatatggagttccgcgttacataacttacggtaaatggcccgcctggctgaccgcccaacgacccccgcccattgacgtcaataatgacgtatgttcccatagtaacgccaatagggactttccattgacgtcaatgggtggagtatttacggtaaactgcccacttggcagtacatcaagtgtatcatatgccaagtacgccccctattgacgtcaatgacggtaaatggcccgcctggcattatgcccagtacatgaccttatgggactttcctacttggcagtacatctacgtattagtcatcgctattaccatggtcgaggtgagccccacgttctgcttcactctccccatctcccccccctccccacccccaattttgtatttatttattttttaattattttgtgcagcgatgggggcggggggggggggggggcgcgcgccaggcggggcggggcggggcgaggggcggggcggggcgaggcggagaggtgcggcggcagccaatcagagcggcgcgctccgaaagtttccttttatggcgaggcggcggcggcggcggccctataaaaagcgaagcgcgcggcgggcgggagtcgctgcgcgctgccttcgccccgtgccccgctccgccgccgcctcgcgccgcccgccccggctctgactgaccgcgttactcccacaggtgagcgggcgggacggcccttctcctccgggctgtaattagcgcttggtttaatgacggcttgtttcttttctgtggctgcgtgaaagccttgaggggctccgggagggccctttgtgcggggggagcggctcggggggtgcgtgcgtgtgtgtgtgcgtggggagcgccgcgtgcggctccgcgctgcccggcggctgtgagcgctgcgggcgcggcgcggggctttgtgcgctccgcagtgtgcgcgaggggagcgcggccgggggcggtgccccgcggtgcggggggggctgcgaggggaacaaaggctgcgtgcggggtgtgtgcgtgggggggtgagcagggggtgtgggcgcgtcggtcgggctgcaaccccccctgcacccccctccccgagttgctgagcacggcccggcttcgggtgcggggctccgtacggggcgtggcgcggggctcgccgtgccgggcggggggtggcggcaggtgggggtgccgggcggggcggggccgcctcgggccggggagggctcgggggaggggcgcggcggcccccggagcgccggcggctgtcgaggcgcggcgagccgcagccattgccttttatggtaatcgtgcgagagggcgcagggacttcctttgtcccaaatctgtgcggagccgaaatctgggaggcgccgccgcaccccctctagcgggcgcggggcgaagcggtgcggcgccggcaggaaggaaatgggcggggagggccttcgtgcgtcgccgcgccgccgtccccttctccctctccagcctcggggctgtccgcggggggacggctgccttcgggggggacggggcagggcggggttcggcttctggcgtgtgaccggcggctctagagcctctgctaaccatgttcatgccttcttctttttcctacagctcctgggcaacgtgctggttattgtgctgtctcatcattttggcaaagaattgcggccgtctcaggccgaattcaagcttgccaccatggggatccttcccagccctgggatgcctgcgctgctctccctcgtgagccttctctccgtgctgctgatgggttgcgtagctgaa**ACCGGT**cacttggattgcaaacctgaattctcgtatgccatagcaaaggacgaaagaattggtcaactgggggctgaaggtctcaccaccacctggaaagaatacaaccacgatttgcaactgaatgacgggaccgttaaggccatttgcgtggcaggttcctttaaagtcacagcacttaatgtggtcagtaggaggtatttggcatcattgcataaggaggctttacccacttccgtgacattcgagctcctgttcgacgggacT**GGTACC**ggctcctccggactgaatgacatctttgaggcccagaagatcgagtggcacgagtccggcggcggatctcttgtaccgcgaggtagttggtcccaccctcagttcgagaagggcggaggctccggcggcggatctggaggaggctcctggtcccacccccagttcgagaaatgataactcgagactagtatcgcgataattcactcctcaggtgcaggctgcctatcagaaggtggtggctggtgtggccaatgccctggctcacaaataccactgagatctttttccctctgccaaaaattatggggacatcatgaagccccttgagcatctgacttctggctaataaaggaaatttattttcattgcaatagtgtgttggaattttttgtgtctctcactcggaaggacatatgggagggcaaatcatttaaaacatcagaatgagtatttggtttagagtttggcaacatatgcccatatgctggctgccatgaacaaaggttggctataaagaggtcatcagtatatgaaacagccccctgctgtccattccttattccatagaaaagccttgacttgaggttagattttttttatattttgttttgtgttatttttttctttaacatccctaaaattttccttacatgttttactagccagatttttcctcctctcctgactactcccagtcatagctgtccctcttctcttatggagatccctcgacctgcagcccaagcttggcgtaatcatggtcatagctgtttcctgtgtgaaattgttatccgctcacaattccacacaacatacgagccggaagcataaagtgtaaagcctggggtgcctaatgagtgagctaactcacattaattgcgttgcgctcactgcccgctttccagtcgggaaacctgtcgtgccagcggatcgatccgctgcattaatgaatcggccaacgcgcggggagaggcggtttgcgtattgggcgctcttccgcttcctcgctcactgactcgctgcgctcggtcgttcggctgcggcgagcggtatcagctcactcaaaggcggtaatacggttatccacagaatcaggggataacgcaggaaagaacatgtgagcaaaaggccagcaaaaggccaggaaccgtaaaaaggccgcgttgctggcgtttttccataggctccgcccccctgacgagcatcacaaaaatcgacgctcaagtcagaggtggcgaaacccgacaggactataaagataccaggcgtttccccctggaagctccctcgtgcgctctcctgttccgaccctgccgcttaccggatacctgtccgcctttctcccttcgggaagcgtggcgctttctcatagctcacgctgtaggtatctcagttcggtgtaggtcgttcgctccaagctgggctgtgtgcacgaaccccccgttcagcccgaccgctgcgccttatccggtaactatcgtcttgagtccaacccggtaagacacgacttatcgccactggcagcagccactggtaacaggattagcagagcgaggtatgtaggcggtgctacagagttcttgaagtggtggcctaactacggctacactagaagaacagtatttggtatctgcgctctgctgaagccagttaccttcggaaaaagagttggtagctcttgatccggcaaacaaaccaccgctggtagcggtggtttttttgtttgcaagcagcagattacgcgcagaaaaaaaggatctcaagaagatcctttgatcttttctacggggtctgacgctcagtggaacgaaaactcacgttaagggattttggtcatgagattatcaaaaaggatcttcacctagatccttttaaattaaaaatgaagttttaaatcaatctaaagtatatatgagtaaacttggtctgacagttaccaatgcttaatcagtgaggcacctatctcagcgatctgtctatttcgttcatccatagttgcctgactccccgtcgtgtagataactacgatacgggagggcttaccatctggccccagtgctgcaatgataccgcgagacccacgctcaccggctccagatttatcagcaataaaccagccagccggaagggccgagcgcagaagtggtcctgcaactttatccgcctccatccagtctattaattgttgccgggaagctagagtaagtagttcgccagttaatagtttgcgcaacgttgttgccattgctacaggcatcgtggtgtcacgctcgtcgtttggtatggcttcattcagctccggttcccaacgatcaaggcgagttacatgatcccccatgttgtgcaaaaaagcggttagctccttcggtcctccgatcgttgtcagaagtaagttggccgcagtgttatcactcatggttatggcagcactgcataattctcttactgtcatgccatccgtaagatgcttttctgtgactggtgagtactcaaccaagtcattctgagaatagtgtatgcggcgaccgagttgctcttgcccggcgtcaatacgggataataccgcgccacatagcagaactttaaaagtgctcatcattggaaaacgttcttcggggcgaaaactctcaaggatcttaccgctgttgagatccagttcgatgtaacccactcgtgcacccaactgatcttcagcatcttttactttcaccagcgtttctgggtgagcaaaaacaggaaggcaaaatgccgcaaaaaagggaataagggcgacacggaaatgttgaatactcatactcttcctttttcaatattattgaagcatttatcagggttattgtctcatgagcggatacatatttgaatgtatttagaaaaataaacaaataggggttccgcgcacatttccccgaaaagtgccacctgg
